# The ENDORSE Feasibility Study: Exploring the Use of M-Health, Artificial Intelligence and Serious Games for the Management of Childhood Obesity

**DOI:** 10.3390/nu15061451

**Published:** 2023-03-17

**Authors:** Konstantia Zarkogianni, Evi Chatzidaki, Nektaria Polychronaki, Eleftherios Kalafatis, Nicolas C. Nicolaides, Antonis Voutetakis, Vassiliki Chioti, Rosa-Anna Kitani, Kostas Mitsis, Κonstantinos Perakis, Maria Athanasiou, Danae Antonopoulou, Panagiota Pervanidou, Christina Kanaka-Gantenbein, Konstantina Nikita

**Affiliations:** 1 School of Electrical and Computer Engineering, National Technical University of Athens, 15780 Athens, Greeceknikita@ece.ntua.gr (K.N.); 2 First Department of Pediatrics, Medical School, National and Kapodistrian University of Athens, Aghia Sophia Children’s Hospital, 11527 Athens, Greece; evichatzidaki@yahoo.gr (E.C.); ppervanid@med.uoa.gr (P.P.); ckanaka@med.uoa.gr (C.K.-G.); 3 Department of Pediatrics, School of Medicine, Democritus University of Thrace, 68100 Alexandroupolis, Greece; 4 UBITECH, Big Data Science & Analytics Unit, 15231 Athens, Greece; 5 Inspiring Earth, Pegneon, 11521 Athens, Greece

**Keywords:** machine learning, genetic algorithm, BMI, multicomponent, self-monitoring, technology, wearables, family-based intervention, childhood obesity

## Abstract

Childhood obesity constitutes a major risk factor for future adverse health conditions. Multicomponent parent–child interventions are considered effective in controlling weight. Τhe ENDORSE platform utilizes m-health technologies, Artificial Intelligence (AI), and serious games (SG) toward the creation of an innovative software ecosystem connecting healthcare professionals, children, and their parents in order to deliver coordinated services to combat childhood obesity. It consists of activity trackers, a mobile SG for children, and mobile apps for parents and healthcare professionals. The heterogeneous dataset gathered through the interaction of the end-users with the platform composes the unique user profile. Part of it feeds an AI-based model that enables personalized messages. A feasibility pilot trial was conducted involving 50 overweight and obese children (mean age 10.5 years, 52% girls, 58% pubertal, median baseline BMI z-score 2.85) in a 3-month intervention. Adherence was measured by means of frequency of usage based on the data records. Overall, a clinically and statistically significant BMI z-score reduction was achieved (mean BMI z-score reduction −0.21 ± 0.26, *p*-value < 0.001). A statistically significant correlation was revealed between the level of activity tracker usage and the improvement of BMI z-score (−0.355, *p* = 0.017), highlighting the potential of the ENDORSE platform.

## 1. Introduction

Childhood obesity is a major public health challenge worldwide. If left untreated, it is linked to numerous health complications, both physical (insulin resistance, dyslipidemia, non-alcoholic fatty liver disease, and arterial hypertension) and psychological (low self-esteem and depression) [1,2]. According to the World Health Organization (WHO), 340 million children and adolescents aged 5–19 were overweight or obese in 2016, thus highlighting the need to develop efficient interventions for the management of childhood obesity [3]. However, conventional interventions are not always accessible due to high costs, commute inconveniences, and lack of facilities. Additionally, 80% of overweight and obese children hesitate to engage in physical activities, therefore requiring the implementation of creative interventions [4].

Multicomponent interventions (dietary, physical activity, educational, and behavioral) constitute first-line treatments for childhood overweight and obesity [1,5,6]. According to the US Preventive Services Task Force, clinically significant weight reduction is considered a drop in BMI z-score by 0.2 units. To achieve this reduction, intensive, multicomponent interventions are required with a minimum of 26 contact hours and a duration of 3–6 months [5]. Smart digital health interventions are considered an effective means for accommodating certain issues raised by the child’s socioeconomic, environmental, and health status through tracking and monitoring physiological, psychological, behavioral, and lifestyle parameters that help healthcare professionals in adapting the treatment plan. For this reason, many studies have explored the use of technological components, such as mobile applications, text messages, websites, and wearables, in weight management programs for adults and children and have produced promising results [7,8,9,10,11]. Especially during the COVID-19 era, e-health programs have become more important in the fight against childhood obesity [12]. Ambulatory care via mobile devices can also contribute to optimizing healthcare processes by saving considerable staff and patient time while endorsing individuals in self-health management [13]. Parental involvement is considered important for the successful weight management of children with obesity, not only in traditional interventions [5,6] but also in digital health interventions [14].

Despite the recognized benefits and advantages of Computerized Decision Support Systems (CDSS) incorporating Machine Learning (ML) techniques [15,16,17], there is a limited number of interventions that have been based on these technologies [18]. The type of interventions have been varying across (i) utilizing Electronic Health Records (EHRs) to produce alerts based on the BMI, (ii) using a table app for educational purposes, goal setting, and videoconferences between family and health coaches, (iii) delivering to the families and health providers a web site for comparing measurements and lifestyle information with the established clinical guidelines, (iv) applying exergames and video-chat in a gaming console with the aim to support self-health management. Some of these interventions have also incorporated wearable activity trackers. ML has been applied for exploring the classification of physical activity, investigating correlations between proximity to a supermarket and weight control and predicting weight. Most of these ML approaches have been based on decision trees, random forests, artificial neural networks, and linear and logistic regressions.

The development of a smart digital multicomponent intervention engaging all the primary agents (e.g., parents and healthcare professionals with different expertise) in childhood obesity management remains challenging. The ENDORSE project faces this challenge through the use of advanced Information and Computing Technologies (ICT), including mobile health (m-health), sensing, gamification, and ML technologies capable of delivering tools and services facilitating weight management while engaging the active involvement of children, parents, pediatricians, nutritionists, psychologists, and endocrinologists. Self-monitoring, goal setting, and positive feedback constitute the core components of the ENDORSE behavioral obesity intervention that are based on the self-determination theory (SDT) [19]. This feasibility study has been conducted to elucidate the potential of technically implementing the ENDORSE innovative software ecosystem into clinical practice while investigating its usability and acceptability. Secondary objectives have been to examine whether participating in this e-health weight management program had an impact on children’s BMI z-score, diet quality, physical activity, screen time, and sleep duration.

## 2. Materials and Methods

### 2.1. The ENDORSE Platform as a Means for the Management of Childhood Obesity

The ENDORSE platform consisted of several modules [20]: (i) a mobile serious game (SG) to promote effective behavioral lifestyle changes in overweight/obese children; (ii) a physical activity tracker facilitating physical activity monitoring and sleep tracking; and (iii) mobile applications for parents and healthcare professionals, enabling remote health monitoring. Apart from the end-user modules, the platform also featured the ENDORSE recommendation system that was responsible for producing personalized content such as reports, messages, and in-game missions. To guarantee data privacy and data security, sensitive information stored on the ENDORSE platform was encrypted, while access to the platform was mediated through the deployment of a dedicated identity and access controller that was also responsible for the registration of the participants.

The ENDORSE SG included a variety of mini-games in the form of educational and action missions. Through the action missions, the user collected in-game currency and food ingredients. The collected food ingredients were used as primary materials in an educational mini-game focusing on preparing a lunch meal. The users were rewarded with regard to their ability to prepare a balanced meal. The in-game currency was spent on avatar customization. The ENDORSE SG also featured space for presenting daily educational personalized messages generated by the ENDORSE recommendation system.

The ENDORSE parental mobile apps provided various functionalities to support self-monitoring, such as logging goal-related achievements and body weight, while exchanging messages with healthcare professionals. The parental mobile app also hosted specific sections for displaying messages generated by the ENDORSE Recommendation System on a daily basis, the dietary plan as drafted by healthcare professionals, and educational material. At their initial log-in to the mobile app, the parents were required to fill in several forms for medical, nutritional, behavioral, and psychological assessment. The ENDORSE mobile app that was available to healthcare professionals had the same template as the parental mobile app that featured further functionalities, such as: (i) editing the medical assessment form; (ii) editing, formatting, sending, and changing the dietary plan; (iii) entering and changing weekly goals; and (iv) sending feedback messages to the parents.

To guarantee data security in the frame of the ENDORSE mobile apps, several security measures were employed, including (1) the establishment of a secure connection between the ENDORSE mobile application, the ENDORSE server, and the ENDORSE Firebase platform instance; (2) the local encryption of the stored data; and (3) enabling the appearance of the name of the participant in the healthcare professionals’ screen only after the healthcare professional proceeded with scanning the unique QR code produced from the participant’s ENDORSE parental mobile app, otherwise an arbitrary alphanumeric code was visible on the screen after having registered the participant in the ENDORSE platform and having associated the participant with specific healthcare professionals.

Physical activity trackers: The Fitbit ACE 2 (for kids) was utilized, which had the ability to track steps, calories, and sleep characteristics (duration and quality of sleep). Fitbit Ace 2 featured Bluetooth connectivity and incorporated memory for up to 7 days of movement data per minute and up to 30 days of total daily activity data. It was waterproof and had a five-day battery autonomy. Physical activity tracking (steps per day) and sleep duration were available, through the cloud, to the ENDORSE platform for further processing from the ENDORSE Recommendation System. Children were advised to wear the activity tracker daily. For younger children (<8 years) and those who did not feel comfortable wearing the activity tracker on a daily basis, a recommendation for a minimum of 2 weekdays and 1 rest day was given.

The ENDORSE Recommendation System leveraged data collected from various sources, including the physical activity tracker, the ENDORSE mobile apps, and the ENDORSE SG. It incorporated an engine able to produce personalized weekly reports providing information regarding the child’s achievements in the ENDORSE SG, physical activity as recorded by means of the physical activity tracker, and weight logs. Personalization was further supported through the integration of an AI-based engine able to select at a weekly basis suitable game missions and messages to be presented to the end users [20]. To this end, a pool of messages was created, including tips and advice on a healthy lifestyle.

### 2.2. Study Design

This ENDORSE study was a 12-week pilot study with a pre- and post-intervention design since data collection occurred at baseline and immediately post-intervention. The main objective was to investigate the potential of utilizing the ENDORSE platform as a supportive means to standardize weight management care of overweight and obese children. This study was driven by Self Determination Theory with an emphasis on providing appropriate plans, meaningful rational, and positive/informational feedback to all participants in a respectful manner [19,21]. The clinical protocol was approved by the bioethics committee of the “Aghia Sophia” Children’s Hospital (protocol number: 4760, date of approval 10 March 2021), and all mothers provided written informed parental consent to participate in this study.

**Figure 1:**
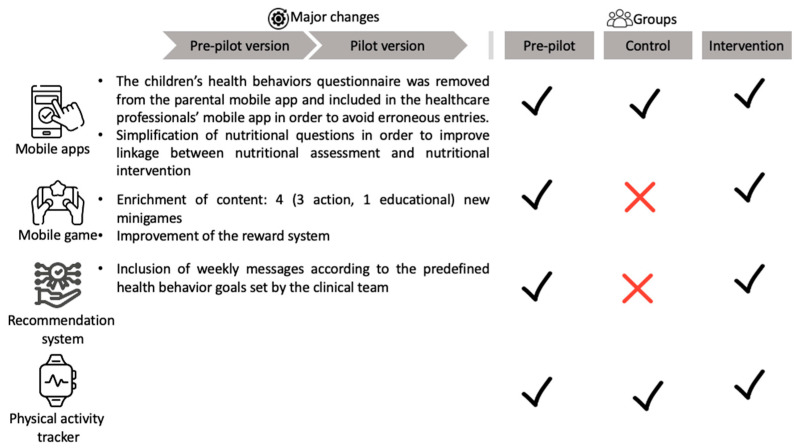
Major changes between pre-pilot and pilot intervention. The ENDORSE modules were utilized by each group for 12 weeks. Images: Flaticon.com (accessed on 10 February 2023).

This study included two phases (pre-pilot and pilot) and was executed from March 2021 to May 2022. The pre-pilot phase aimed at the clinical implementation of the ENDORSE platform in order to recognize and resolve technical issues while receiving valuable feedback from the participants that led to improvements (Figure 1) toward releasing the platform’s pilot version. The recruiting and monitoring procedure was conducted at the Obesity outpatient Clinic of the First Department of Pediatrics of the National and Kapodistrian University of Athens at the “Aghia Sophia” Children’s Hospital in Athens, Greece. All participants were recruited from the Obesity outpatient Clinic (treatment-seeking population). The majority of the participants resided in urban settings, i.e., most were residents of Athens (90%, *n* = 40).

The USSF sample size calculator for pre- and post-intervention (paired *t*-test) was used [22] in order to estimate the sample size that should be recruited for producing statistically significant evidence on BMI z-score changes. The threshold probability for rejecting the null hypothesis was 5%, while the probability of failing to reject the null hypothesis under the alternative hypothesis was equal to 20%. A medium value (e.g., 0.5) of effect size was applied, and the SD of the BMI z-score reduction was set to 1. The estimated sample size was equal to 34, yet the total recruited sample consisted of more participants (e.g., 50 chidren and their mothers) in order to account for potential dropouts. Nonrandomized recruitment was used to enroll participants into 3 consecutive groups that applied different versions of the ENDORSE platform for a time period of 12 weeks (Figure 1). In this pilot study, the differences between the active control group and the intervention group were the absence of the ENDORSE SG and the de-activation of the ENDORSE Recommendation System. In the active control group, personalized messages were sent at the beginning of each week to the participating mothers by a member of the clinical team (pediatrician or nutritionist). In the intervention group, the weekly messages were designed by the clinical team (with relevant content to the pre-specified weekly behavioral goal) and were sent automatically to all participants by the ENDORSE Recommendation System at the beginning of each week. In both groups, mothers were able to exchange messages with the clinical team via the application.

Children aged 6–14 years with a BMI > 85th centile were eligible to participate in this study. Overweight and obese were classified according to the age- and sex-specific definition of the International Obesity Task Force (IOTF) [23]. The exclusion criteria included secondary causes of obesity such as endocrine causes (hypothyroidism, Cushing syndrome, growth hormone deficiency), known genetic syndromes linked to obesity (Down syndrome, Prader Willi syndrome), and serious developmental disorders (severe Autism Spectrum Disorders (ASD) or severe Attention Deficit Hyperactivity Disorder (ADHD)).

### 2.3. Measures

#### 2.3.1. Medical Assessment

At baseline and at the end of this study, a full clinical assessment was performed by a pediatric endocrinologist, including the assessment of the pubertal stage. Children were grouped as prepubertal (Tanner stage 1) and as pubertal (including Tanner stages 2, 3, 4, and 5). Girls were staged according to breast development and pubic hair growth, and boys were staged according to pubic hair growth and male genital stages [24].

Anthropometrics: Height was measured to the nearest 1 mm by means of a stadiometer (Holtain Limited) while children were standing without shoes, with their backs to the wall, and with their bodies up straight. Body weight was measured to the nearest 0.1 kg in light clothing using a mobile digital scale (Tefal Bodysignal). Each BMI was standardized by conversion to a z-score (BMI z-score) in groups defined by age and sex according to the Centers for Disease Control and Prevention (CDC) growth charts 2000 [25]. Because BMI z-scores are known to be inaccurate at values greater than the 97th centile, adjusted z-scores were used for these children (*n* = 46, 92% of the whole sample) [26]. Waist circumference was measured with an elastic tape at the point halfway between the last palpable rib and the tip of the iliac crest with an accuracy of 0.1 cm. The waist-to-height ratio (WHR) above 0.5 was considered a measure of central adiposity [27]. Blood pressure (mmHg) was measured with an electronic blood pressure meter (Microlife Gentle). Two measurements were taken at each visit, and the mean value was documented.

#### 2.3.2. Nutritional Assessment and Dietary Intervention

At baseline, a thorough nutritional assessment was made by a nutritionist. A diet history interview was used to collect information on dietary habits, eating behavior, and parental feeding practices in order to design a nutritional intervention and an individualized meal plan. The Mediterranean dietary pattern, as described in the Greek National Dietary Guidelines for Infants, Children, and Adolescents, formed the basis of the individualized meal plan with macronutrient balance (15–20% protein, 30–35% fat, 50–55% carbohydrates) [28]. Energy requirements were calculated using the equations of the Institute of Medicine for use in the pediatric population with excess weight [29] and adjusted according to weight maintenance or weight loss goals [30]. The dietary plan included a wide variety of foods relevant to each child’s preferences to encourage dietary adhesion.

The nutritional intervention was designed according to the wishes, abilities, and goals set for each child/adolescent. In addition, it aimed to reinforce feeding practices that promote parental involvement, self-regulation, and autonomy of children and adolescents against the implementation of restrictive feeding practices [31,32].

#### 2.3.3. Children’s Health Behaviors Questionnaire

In order to evaluate food habits and other health behaviors of the children (exercise, sedentary life, sleep), a clinical questionnaire was used, specifically designed for the needs of this study, which was completed by a member of the clinical team together with the mothers at the beginning and the end of the intervention. Food habits were assessed by questions based on the National Dietary Guidelines of Greece for Children and Adolescents and indicative serving suggestions by age group as reported in the Guidelines [28,33]. It included 22 questions regarding food groups, food items of interest, and frequency of consumption with the aim to picture the National Dietary Recommendations described briefly in the “Ten steps to healthy eating for children and adolescents” [28]. Weekly consumption of fruits, vegetables, whole grains, fast food, sugar-sweetened beverages (SSB), and energy-dense but low-nutrient packaged products (highly processed snacks) were used as indicators of the children’s diet quality [1]. The same questionnaire included questions about the children’s weekly frequency of organized physical activity, as well as the daily hours that children spent in front of screens for entertainment purposes, the children’s daily sleep duration, and the quality of their sleep (see Supplementary File S1, Health behaviors questionnaire).

#### 2.3.4. Psychological Assessment

At baseline, a thorough psychological assessment was performed by a health psychologist, who assessed psychologically the eligibility of mothers and their children for their participation in this study based on a clinical interview with structured open-ended questions. By the time the mothers were interviewed by the psychologist, they had completed several psychometric tests via the application for themselves: PHQ-9: Greek version of the Patient Health Questionnaire −9 by Kroenke et al., which is a screening tool for detecting depressive symptoms in adults [34,35]; EAT-26: Eating Attitudes Test-26 by Garner et al. [36], a Greek version by Simos [37], which is a screening tool for detecting eating disorders in adults; the Comprehensive Feeding Practices Questionnaire by Musher-Eizenman et Holub [38], a Greek version by Michou et al. [39], that is a questionnaire evaluating 6 feeding practices (monitoring, control of feeding by the child, pressure to eat, restriction in food intake, use of food as a reward and/or for emotional regulation and guidance for healthy eating); and one questionnaire for their child—SDQ: Parent’s version of the Strengths and Difficulties Questionnaire by Goodman [40], a Greek version by Bibou-Nakou et al. [41], which is a screening tool for detecting behavioral and emotional problems in children and adolescents. Mothers also completed a questionnaire via the application specially designed for the needs of the research, which included questions about sex, age, weight, height, nationality, marital status, level of education, and type of work. The body mass index of the parents was calculated based on self-reported weight and height: BMI = weight (kg)/height^2^ (m^2^). Their categorization into normal weight (BMI: 18.5 kg/m^2^ to 24.9 kg/m^2^), overweight (BMI: 25 kg/m^2^ to 29.9 kg/m^2^), and obese (BMI ≥ 30 kg/m^2^) was done according to the definition of overweight and obesity of the World Health Organization [3]. The results were discussed with the psychologist in person or over the telephone. At the end of the intervention, a second psychological assessment was performed by the same psychologist, and mothers were asked again to complete these psychometric questionnaires via the application. During the interview, emphasis was given to known psychosocial factors associated with childhood obesity (bullying, weight stigma, and depressive symptoms) [42,43,44].

Although the risk of developing an eating disorder following family-based obesity interventions is extremely low [45,46], there are no data from digital interventions addressing this issue [7,9]. During the initial psychological and medical/nutritional assessment, emphasis was given to parents that weight reduction should be gradual even for children with severe obesity (no more than 1 kg/week). Parents were advised to weigh their children once weekly. If weight loss was more than 1 kg/week, they were advised to contact a member of the clinical team for a thorough evaluation for excessive energy restrictions (meal skipping, purging, fasting, excessive exercise, etc.), as recommended by the American Academy of Pediatrics [47].

### 2.4. Study Implementation

At the time this ENDORSE study was conducted, “Aghia Sophia” Children’s Hospital was a reference center for children with COVID-19 infection. The applied health protection measures to minimize contamination with the SARS-CoV2 virus hampered the recruitment and monitoring procedures making it difficult to enroll the intervention and control groups simultaneously. For this reason, the participants of the two pilot groups were recruited and monitored consecutively. At the beginning of this study, the mother participants had one 90-min face-to-face session with the clinical team (pediatrician and nutritionist) in order to be trained in utilizing the ENDORSE platform (activity tracker, serious game, app). Moreover, during these sessions, the weight goals were set together with the mothers and in accordance with the American Academy of Pediatrics guidelines [30]. Health behavior goals (Table 1) were also explicitly discussed, and the importance of monitoring the goals and weight was thoroughly explained to participants.

#### 2.4.1. Self-Monitoring of Behavior and Outcome

The mother participants were responsible for entering the behavioral goals in the ENDORSE parental mobile application on a daily basis and for monitoring their child’s weight on a weekly basis. Children were asked to monitor their physical activity (steps/day) via the physical activity tracker.

In the pre-pilot phase, the weekly goals were jointly defined by the mothers and a member of the clinical team. Mothers were encouraged to choose no more than 2 goals each week for monitoring. The clinical team had the opportunity to change weekly goals via the app at any given time during the 12 weeks of intervention at the mother’s request. In the pilot phase, all weekly goals for monitoring were set by the clinical team at the beginning of the intervention in a prespecified order (each goal was monitored by the mothers for 2 consecutive weeks): physical activity plus screen time goal; breakfast goal; mid-morning snack goal; lunch goal; afternoon snack goal; and dinner goal. Mothers were still given the opportunity to change weekly goals via the application at any given time during this study.

Positive and informational feedback constituted an important part of the self-monitoring procedure. The weekly reports that were sent to the mothers via the endorsed recommendation system contained informational feedback, while the messages sent by the clinical team were both positive and informational.

#### 2.4.2. Educational Material

At the time of the face-to-face sessions, an educational booklet was given to mothers as a guide for optimal weight management (also available in pdf version via the mobile application). The educational material was developed by the clinical team based on National Dietary Guidelines [28] and aimed to educate mothers and children/adolescents to improve family dietary habits. Emphasis was given on diet quality, food group education (e.g., servings of fruit, vegetables, whole grains, nuts, etc.), portion size education, energy-dense nutrient-poor snacks and sugar-sweetened beverage reduction, and label reading. Recipes with easy-to-prepare healthy choices for main meals and snacks, together with a shopping list, were also provided to all participants. The educational material included a brief description of parental feeding practices according to the recent classification by Di Pasquale et Rivolta following SDT principles [31]: Relatedness—enhancing food parenting practices (family meals, child’s involvement in preparing meals); Competence—enhancing food parenting practices (clear and consistent rules related to food, availability and accessibility of healthy food, nutrition education, parental modeling); Autonomy—enhancing food parenting practices (guided choices, discussing and negotiating with the child food choices). The educational material also included advice to parents about eating behaviors that have a genetic basis and affect appetite in different ways (low satiety responsiveness, food responsiveness, and food fussiness) [49] (see Supplementary File S1, Educational material).

### 2.5. Data Analysis

#### 2.5.1. Descriptive Statistics and Pre-Post Intervention

Descriptive statistics were used to assess baseline participant characteristics (demographical, clinical, and behavioral), separated according to group (means ± SD, median with 25th and 75th centile or as absolute values with percentages). For this pilot study, the Chi-square test was used for categorical variables, while the Independent sample *t*-test was used for normally distributed variables and the Mann–Whitney U test for non-normally distributed variables. The Shapiro–Wilk test was applied to check for normality.

Paired sample *t*-tests were deployed to assess pre- and post-intervention changes within each of the three groups for normally distributed variables and the Wilcoxon test for not normally distributed variables. All *p*-values were two-sided, and the level of significance was set at 0.05.

Statistically significant correlations between the degree of adherence of all participants (*n* = 45) and pre- vs. post-intervention changes in the BMI z-score and health behaviors (intake of fruits, vegetables, fast food, highly processed snacks, and SSBs, changes in physical activity, screen time, sleep duration) were explored via the Spearman’s Rank Correlation test.

For the statistical analysis of health behaviors (diet, physical activity, screen use, and sleep), categorical variables were transformed into ordinal variables according to the following assumptions: 1–2 times/day: 1.5; 3–4 times/day: 3.5; 5–6 times/day: 5.5; 7–8 times/day: 7.5; >9 times/day: 9.5; 1–2 times/week: 1.5; 3–4 times/week: 3.5; 5–7 times/week: 6; never/rarely: 0; 60–120 min/day: 90; 30–60 min/day: 45; <30 min/day: 15; >4 h/day: 4.5; 3–4 h/day: 3.5; 2–3 h/day: 2.5; 1–2 h/day: 1.5; <1 h/day: 0.5; <7 h/day: 6.5, 7–8 h/day: 7.5; 9–11 h/day: 10.

#### 2.5.2. Feasibility and Acceptability

The feasibility of the ENDORSE platform was measured by means of adherence to this study’s protocol, attrition rate, and perceived helpfulness. The criteria for the acceptability were based on Davis’ theory of the Technology Acceptance Model focused on the ease of use and perceived usefulness as rated by the participants [50].

The adherence to the ENDORSE intervention was measured by means of frequency of usage. To this end, objective usage metrics were identified and estimated based on the data records for each module. Specific usage metrics of the parental mobile app included the number of days of usage, the number of days containing self-monitoring data (weight, goals), and the number of messages that were exchanged between the clinical team and the participants. Regarding the physical activity tracker, the number of nights with sleep recordings, the average time of sleep per day (min), the average time of usage per day (hours), the number of days with step recordings, and the average steps per day were calculated. The usage metrics for the ENDORSE SG were identified as the days of usage and the number of mini-games (action, educational) that were completed by the children.

Based on the above usage metrics, an adherence score was estimated that reflected the number of days of usage for each module. Specifically, the days of usage of the ENDORSE parental mobile app (1) were calculated as the maximum between the total days containing self-monitoring goal-related records (goal monitoring) and the weekly weight records (weight monitoring):

score_mobile_app = max (7 × weight_monitoring, goal_monitoring)(1)

Regarding the adherence score to the physical activity tracker (3), the product between the average daily usage (h) and the total days of usage was divided by a predefined minimum daily hour of active usage (10 h):

score_activity_tracker = average_daily_usage × total_days_of_usage/10(2)

The adherence levels were estimated by applying specific thresholds on the obtained individual adherence scores: low (<25 days); medium (between 25 and 50 days); high (>50 days). These thresholds were common across all modules except for the case of the physical activity tracker usage in the pre-pilot phase, as it was applied by the participants for a longer period of up to 120 days since the duration of the pre-pilot phase was extended due to technical issues that hampered the functionality of the ENDORSE platform. Therefore, the corresponding thresholds were set as 40 and 80 days. The individual adherence levels were encoded in 1, 2, and 3 for low, medium, and high, respectively, in order to estimate the overall adherence score that was calculated by adding the corresponding encoded levels. Low, medium, or high adherence levels regarding the combined use of the physical activity tracker and the parental ENDORSE mobile apps were obtained by applying thresholds 2 and 4. Taking into consideration the low adherence to the ENDORSE SG that was observed over almost all children, one child had more than 25 days of usage and was considered to be an outlier, and it was excluded from the estimation of the overall adherence score and level that were used to explore statistically significant correlations.

Perceived usefulness, ease of use, and perceived helpfulness were obtained using a customized self-report 5-point Likert scale questionnaire post-intervention (see Supplementary File S1, Postintervention feasibility questionnaire). The statistical analysis was performed in Python and Excel.

## 3. Results

### 3.1. Baseline Characteristics

The baseline characteristics of the recruited participants are depicted in Table 2 for both pilot phases. The pre-pilot phase included 20 children (60% boys) with a mean age of 11 years. The mean BMI z-score was 2.85, while all children had a waist-to-height ratio greater than 0.5. According to the IOTF criteria [23], 55% of the children were obese and 45% severely obese (>120% of the 95th centile for height and age). Children’s mothers had a mean age of 44 years and BMI of 30.7, while 95% of them were Greek and 65% were married. Most mothers (60%) were secondary school graduates, and 70% of them were employed. A total of 30 children (40% boys) with a mean age of 10 years participated in the pilot phase. The mean BMI z-score in the control group was 2.71, while in the intervention group was 2.89. All children had a waist-to-height ratio greater than 0.5. According to the IOTF criteria [23], 33.3% and 46.7% of children were severely obese in the control and intervention groups, respectively. There were statistically significant differences between the groups in terms of age and height (*p* < 0.05). The participating mothers of this pilot study (*n* = 30) had a mean age of 43.6 years and a mean BMI of 29.6, while 96.7% were Greek, 86.7% married, and 46.7% were university education graduates.

**Figure 2:**
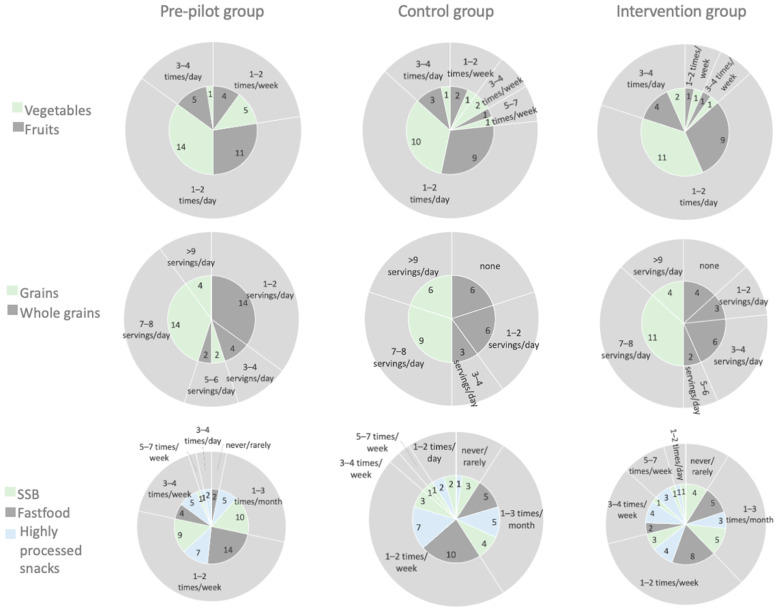
Baseline dietary habits. The actual numbers of participants in each category are depicted in the inner pie chart.

Figure 2 illustrates the baseline dietary habits of the recruited participants. Prior to the pre-pilot phase of this study, 55% and 60% of the children consumed one–two times/day a serving of fruit and vegetables, respectively, which was lower than the target intake of two–three servings/day for their age according to the National Dietary Guidelines [28]. In addition, 90% of children reported an intake of more than seven servings of grains/day, while only one–two servings/day were reported as whole grains for 70% of children. For 90% of the children, the intake of fast-food products was more than once a week, while the intake of a serving of processed snacks for 40% of the children was more than three times a week. Additionally, 50% of the children reported consuming a portion of SSB more than once a week. Prior to the pilot phase of this study, 80% of the children in the control group and 73.4% of the children in the intervention group consumed a portion of fruit at a frequency equal to or less than one–two times a day without a statistically significant difference between the groups. Moreover, the largest percentage of children for both pilot groups reported consuming vegetables at a frequency equal to or less than one–two times a day. All the children for both pilot groups reported an intake of more than seven servings of grains per day, while only 80% of children in the control group and 46.7% in the intervention group reported one–two servings/day of whole grains. Regarding the intake of fast food and highly processed snacks, the majority of children consumed a portion at a frequency equal to or greater than one–two times a week, while the intake of a portion of SSB was of similar frequency for the majority of children. There was no statistically significant difference between the control and intervention groups for all food groups.

**Figure 3:**
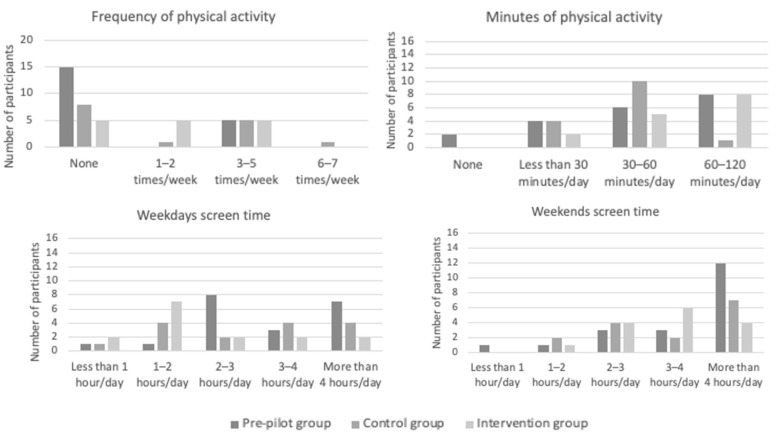
Baseline health behaviors in terms of physical activity and screen time.

Other health behaviors in terms of physical activity, screen time, and sleep duration (Figure 3) were also assessed at baseline. Focusing on the pre-pilot group, 60% of children exercised less than 1 h per day, and 75% of them did not perform systematic physical activity. In addition, 90% of children spent more than 2 h a day in front of screens, both on weekdays and on the weekends. A total of 65% of children slept less than recommended for their age; however, most children (75%) had no sleep difficulties. Regarding the pilot groups, 93.3% of children in the control group and 46.7% of children in the intervention group exercised less than 1 h a day. The number of participants performing physical activity less than 30 min per day was statistically significantly higher in the control group than in the intervention group. Additionally, the majority of children reported more than 2 h of screen time per day on both weekdays and weekends (66.7% and 86.7% for the control group versus 40% and 93.3% for the intervention group, respectively). Regarding sleep time, 46.7% of children for both groups slept less than recommended for their age; however, the majority of children (93.3% vs. 86.7%) had no sleep difficulties, with no statistically significant differences between groups.

### 3.2. Adherence Results

This ENDORSE pilot study had a low attrition rate corresponding to 10%, 13.33%, and 6.66% in the pre-pilot, control, and intervention group, respectively. The degree of adherence was estimated by means of calculating particular metrics indicative of the frequency of usage of each ENDORSE module (e.g., parental mobile app, activity tracker, game).

#### 3.2.1. Usage Metrics

Table 3 presents the usage metrics relevant to the parental mobile app. Within the frame of this pre-pilot study, a small percentage of participants had zero usage, contrary to this pilot study, where all participants interacted with the mobile app. An increased variability was observed among participants in terms of frequency of use since a high standard deviation was estimated regarding days of usage, weight registration, and monitoring of weekly goals. A comparison between the pre-pilot and pilot groups was conducted in order to investigate whether the pilot version of the ENDORSE parental mobile app promoted higher adherence than the corresponding pre-pilot version. Although no statistically significant differences were revealed, all the usage metrics were improved, demonstrating that substantial enhancements were applied toward releasing the pilot version of the ENDORSE platform.

**Figure 4:**
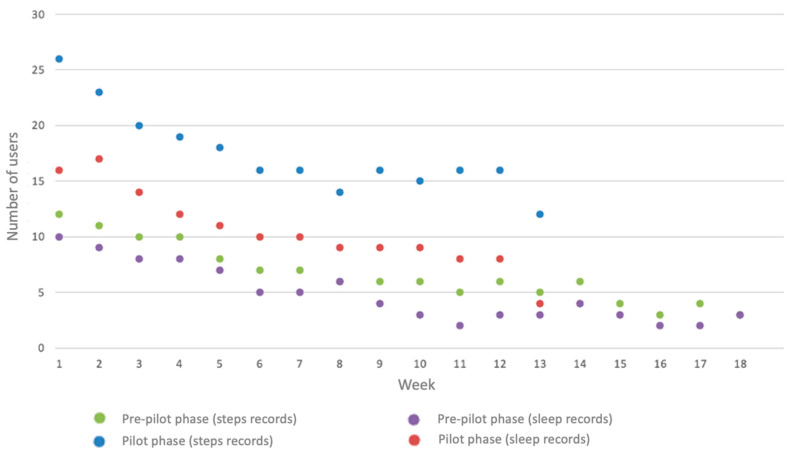
Number of participants applying the physical activity tracker per intervention week.

Several usage metrics were extracted based on the sleep minutes and the number of steps as recorded by means of the physical activity tracker (Table 4). A percentage of the participants (20%) did not apply the physical activity tracker during the day, while 24.44% of them didn’t have sleep records. The number of days with step records varied among the participants, yet the average time of usage per day was adequately high for most of them. The average time of sleep per day (7 to 8 h) was marginally insufficient, and the average steps per day were mainly lower than the target value of 10,000 steps. Figure 4 depicts the number of participants applying the physical activity tracker per intervention week. An overall reduction in the users was observed as the intervention weeks advanced, reflecting the users’ initial excitement and its progressive decrease.

The control group demonstrated the highest, yet not statistically important, usage metrics against those achieved by the pre-pilot and intervention groups. This was justified through the participants’ intensive communication with the medical team (number of communication messages: 4.23 ± 5.64) that promoted their adherence.

The usage metrics relevant to the ENDORSE game were extracted based on the days of interaction with this module and the number of completing the action and educational mini-games (Table 5). Low adherence was observed in the pre-pilot group due to the limited content of the game. A statistically significant difference was observed between the pre-pilot and the intervention group in terms of days of usage (3.86 ± 3.96 vs. 14.57 ± 8.93), highlighting that the enriched game content improved adherence. However, sustainability in participants’ engagement was not achieved, and for this reason, the usage metrics were low.

#### 3.2.2. Level and Score of Adherence

**Figure 5:**
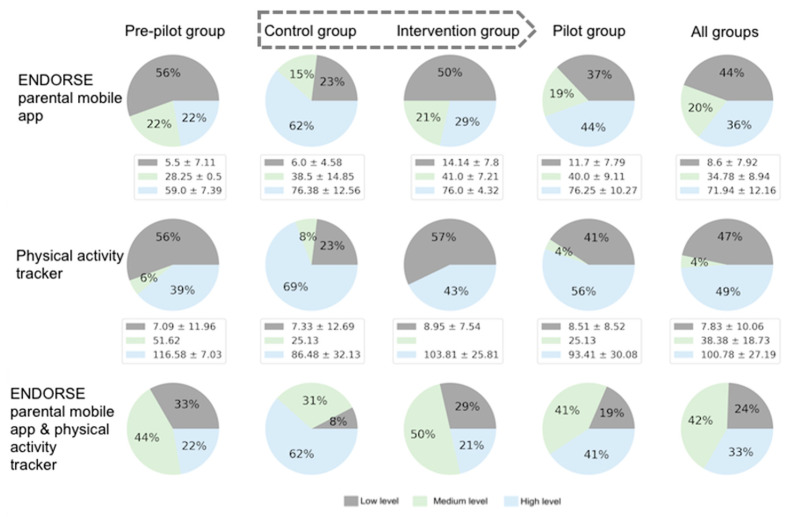
Level and score of adherence. Each pie chart depicts the percentage of participants in each corresponding level, while the respective scores of adherence are presented as legends in terms of mean and standard deviation. Some pie charts don’t add up to 100% due to periodic numbers rounding.

The level and score of adherence for each module (ENDORSE mobile app, physical activity tracker) and group are presented in Figure 5. The overall adherence level across both modules is also depicted. A high percentage of participants (75%) belonged to medium to high overall adherence levels. Most of the participants (62%) in the control group presented a high adherence in terms of utilizing the physical activity tracker and the ENDORSE parental mobile app. Improvement in adherence levels was observed between the pre-pilot and pilot groups since the percentage of high adherence increased from 22% to 41%, and the percentage of low adherence decreased from 33% to 19%.

Statistically significant correlations between the score of adherence and pre- vs. post-intervention changes in the BMI z-score and health behaviors (intake of fruits, vegetables, fast food, highly processed snacks, and SSBs, changes in physical activity, screen time, and sleep duration) were explored. In this direction, Spearman’s Rank Correlation test was applied to the obtained outcomes over all participants. A statistically significant correlation between the score of adherence and the BMI z-score change was revealed (−0.299, *p* = 0.046), yet there were no statistically significant correlations in terms of changes in health behaviors. The changes in BMI z-score were not statistically correlated with changes in health behaviors. A statistically significant correlation was revealed between the physical activity tracker’s average usage and changes in BMI z-score (−0.355, *p* = 0.017).

#### 3.2.3. Acceptability

Table 6 presents a summary of the participant responses to the postintervention questionnaire for the pre-pilot and intervention groups, respectively. Although this summary provides a subjective estimation of the level of acceptance, it could be inferred that the participants found the ENDORSE modules sufficiently useful, helpful, and easy to use. In addition, statistically significant differences were revealed between the pre-pilot and pilot versions of the ENDORSE game and parental mobile app, demonstrating that substantial improvements were applied.

### 3.3. Changes in Anthropometrics and Health Behaviors

#### 3.3.1. Pre-Pilot Study

Following the intervention, the mean BMI z-score of children decreased significantly (−0.24, *p* = 0.001), while no other significant changes were observed in the anthropometric variables. With regard to dietary habits, fruits increased by 0.6 portions/day (*p* < 0.05) and vegetables by 0.62 portions/day (*p* < 0.05), while intake of fast-food products decreased by 0.5 portions per week (*p* < 0.05). In addition, children in this pre-pilot study increased their minutes of physical activity (mean 26.67 min/day) and sleep time (mean 0.81 h/day) while decreasing the time spent in front of screens (*p* < 0.05), both on the weekdays (mean decrease 0.61 h/day) and on the weekends (mean decrease 0.78 h/day).

#### 3.3.2. Pilot Study

The change in BMI z-score of children in the intervention group was −0.16 (*p*-value = 0.002), while in the control group, BMI z-score decreased by −0.21 (borderline significant, *p*-value = 0.068), but between groups, there was no statistically significant difference. No statistically significant differences were observed regarding the other anthropometric characteristics. Regarding the dietary intakes, mean fruit and vegetable intake increased (mean increase of 0.61 and 0.79 servings/day for the control group vs. 0.64 and 1.03 servings/day for the intervention group, respectively) with a statistically significant increase (*p* < 0.05) within both groups. However, there was no statistically significant difference in fruit and vegetable intake between the control and intervention groups. A borderline significant difference between the groups was found in highly processed snacks, where a greater mean reduction was noted in the intervention group (−0.79 vs. −0.5 servings/week, *p* = 0.068) and in SSB, where a greater reduction was noted in the control group (−2.81 vs. −0.32 servings/week, *p* = 0.068). Physical activity increased for both groups (mean increase in physical activity 34.62 vs. 11.79 min/day for the control and intervention groups, respectively) with a statistically significant increase within groups (*p* < 0.05). Additionally, screen time decreased over the week with a mean decrease of 0.69 versus 0.07 h per day for the control and intervention groups, with a statistically significant decrease within groups (*p* < 0.05). Regarding sleep time, there was a mean increase of 0.38 h/day for the control group and 0.36 h/day for the intervention group (*p* > 0.05). A borderline significant difference existed between the groups in terms of minutes of physical activity (*p* = 0.061) and screen time during the week (*p* = 0.085); however, the largest difference was noted in the control group.

#### 3.3.3. Overall Changes

Considering the whole population of participants (n = 45) in both phases, there was a clinically significant reduction in BMI z-score (mean BMI z-score reduction: −0.21 ± 0.26, *p*-value < 0.001), an increase in fruit intake (mean increase in daily servings 0.62, *p* < 0.001), in vegetable intake (mean increase in daily servings 0.80, *p* < 0.001), and a decrease in fast food intake (mean decrease in weekly consumption −0.22, *p* = 0.042). Additionally, there was an increase in physical activity (mean increase in minutes of physical activity 24.33, *p* < 0.001), a reduction in hours of television viewing on weekdays (mean reduction in daily hours of television viewing −0.47, *p* = 0.005), and an increase in sleep hours (mean increase in sleep hours 0.54, *p* = 0.005). Furthermore, at the time of the second psychological assessment (in-person or over the telephone) of the mothers who completed the program (n = 45), no adverse psychological events were mentioned to the teams’ psychologists, and all mothers were satisfied with their participation in the program.

## 4. Discussion

### 4.1. Main Findings

The outcomes of this ENDORSE feasibility study revealed important findings that can help elucidate the advantages and challenges regarding the use of technological solutions in the management of childhood obesity. Engagement and adherence were demonstrated to play crucial roles in BMI reduction, a finding that is in accordance with the reported results obtained by metadata analysis [7]. However, the authors in this meta-analysis highlight the fact that most studies do not consistently measure and report adherence data, and when reported, adherence rates are often suboptimal and decline over time [7]. In order to provide reliable measures of adherence, several objective indicators were obtained reflecting the frequency of usage for each module and for the overall ENDORSE platform.

The level of adherence varied among the participants, while in some modules (e.g., physical activity tracker), a reduction over time was observed. Comparing the adherence to the ENDORSE protocol with the protocol that applied other CDSS-based interventions for the management of childhood obesity, it can be implied that a low attrition rate (10%) was achieved while most of the studies (62.5%) were labeled as weak to moderate in terms of dropouts [18]. Additionally, the ENDORSE intervention plan was characterized as helpful, useful, and easy to use by the participants. This is particularly important, taking into consideration the high prevalence of severe obesity in the recruited samples.

The benefit of applying wearables for physical activity monitoring toward weight controlling in childhood obesity was also demonstrated within the ENDORSE feasibility trial since a statistically significant correlation was revealed between the activity tracker average usage and the reduction of the BMI z-score (−0.355, *p* = 0.017). This finding is in accordance with the outcomes of a recent systematic review and meta-analysis investigating the effectiveness of interventions supported by activity trackers in preventing and treating childhood obesity [51]. Wearable device interventions had statistically significant beneficial effects on BMI, BMI z-score, body weight, and body fat.

Another important finding concerns the high adherence observed in most participants in the control group (62%). Τhe active control group included personalized messages sent by the clinical team on a weekly basis to all participating mothers via the application. This has probably generated a more frequent exchange of messages between the mothers and the clinical team compared to the intervention group, where the weekly messages were fully automated. Taking into consideration that our sample consisted mainly of treatment-seeking obese (50%) and severely obese (42%) participants with special needs, having a personal coach can contribute significantly to increased adherence. Similar findings were observed in a CDSS-based intervention clinical trial incorporating a health coach who performed four telephone calls with the participants’ parents within one year of intervention, and this led to better weight outcomes [52].

The low engagement with the ENDORSE’s SG highlights the great challenge of designing an SG featuring a balance between user attractiveness and scientific background in accordance with the existing literature [53,54,55]. However, the significant enhancement that was observed in terms of engagement in the pilot version compared to the pre-pilot version supports the effectiveness of the SG’s gameplay flow and conceptual framework. Potential improvements could refer to the implementation of more mini-games (missions) and the improvement of the artwork.

Regarding the secondary objectives of this ENDORSE feasibility study, the e-health intervention managed to achieve clinically and statistically significant BMI z-score reduction (mean BMI z-score reduction: −0.21, *p*-value < 0.001) and significant change in health behaviors in all participants. This is in accordance with results from meta-analytic studies, showing that digital health interventions can be effective in treating childhood obesity [7,11,14]. A recent meta-analysis of 32 randomized controlled trials of technology-based interventions for childhood obesity treatment found a small but significant effect on weight outcomes (d = −0.13, *p* = 0.001), although 27 of 33 treatment studies (79%) did not find significant differences between treatment and comparators [7]. Another meta-analysis of nine clinical studies using self-monitoring via mobile health technologies in pediatric weight management also found a small but significant overall effect size (d = 0.42) of the interventions on weight status [56].

Regarding health behaviors, overall (n = 45), there was an increase in fruit intake (mean increase in daily servings 0.62, *p* < 0.001), an increase in vegetable intake (mean increase in daily servings 0.80, *p* < 0.001), a decrease in fast food intake (mean decrease in weekly consumption −0.22, *p* = 0.042), an increase in physical activity (24.33 min/day, *p* < 0.001), a reduction of screen exposure on weekdays (−0.47 h/day, *p* = 0.005) and an increase in sleep time of 0.54 h/day (*p* = 0.005). A recent review of the impact of family-based digital interventions for obesity prevention and treatment on obesity-related outcomes in primary school-aged children [11] reported significant improvements in physical activity in two intervention studies [57,58] and in diet quality in terms of increasing energy from healthy core foods and decreasing energy from energy-dense nutrient-poor foods in one intervention study [59], while no significant effects were reported for screen time in one intervention study [60]. Specifically regarding fruits and vegetable consumption, a recent systematic review with meta-analysis of in-site and digital nutrition interventions in children and adolescents with overweight or obesity, including the intervention groups of 34 randomized control trials (RCT), reported an increased fruit and vegetable intakes from 0.6 to 1.5 servings/day at time periods up to 12 months from baseline [61].

### 4.2. Limitations

This ENDORSE feasibility study was conducted under highly challenging and difficult conditions imposed by the application of public measures to control the COVID-19 pandemic making it thus impossible to consider a control group following the standard of care due to the restricted clinic visitation policies. The size of the recruited sample was also affected and limited to a feasible number of participants. Furthermore, the participant’s lifestyle (e.g., diet, physical activity) was strongly influenced by the containment measures causing major changes in physical activity and diet with respect to those under normal conditions.

The categorization based on the BMI could also be considered as a limitation since it was not indicative of body fat levels and fat-free mass. Children with normal weight might have low muscle levels, while active overweight children might have lower body fat and higher fat-free mass levels.

Positive changes in fruit and vegetable intake and reduction in energy-dense nutrient-poor foods are of high importance as they have the potential to positively influence child weight status [1]. However, the food intakes of the children were assessed by an interview-delivered semi-quantitative questionnaire formed specifically for the needs of our study. The dietary outcomes were derived from the frequencies of consumption of food items or food group sections of the questionnaire converted to continuous variables for the need of the analysis, as mentioned in the methodology. This conversion must be kept in consideration with the interpretation of the results. Despite the limits of this dietary assessment method, the dietary intervention successfully focused on food-based guidance rather than nutrient-focused advice, as dietary interventions targeting intakes of specific food groups are more likely to result in the intended change in the targeted food(s) [61].

## 5. Conclusions

The feasibility of implementing the ENDORSE integrated platform in clinical practice was investigated in terms of adherence and impact on effective weight and behavioral changes. The obtained results demonstrate the potential of utilizing advanced Artificial Intelligence, m-health, and gamification technologies toward the creation of an innovative software ecosystem with the capacity to support healthcare professionals in monitoring and decision-making while empowering self-health management in childhood obesity. Future work is mandatory in means of conducting large-scale clinical trials in order to explore the effectiveness and sustainability of the ENDORSE platform.

## Figures and Tables

**Table 1:** The ENDORSE health behavior goals.

Health Behavior Goal	Brief Description of Goals
Physical activity	Children were advised to engage in medium to high intensity physical activities at least 60 min per day (or to approach the 10,000 steps/day goal) [1,48].
Screen time	Mothers and children were advised to limit screen time for recreational reasons to less than 1–2 h daily) [1].
Breakfast	Children were advised to make daily healthy breakfast choices according to their personalized dietary plan. Mothers were advised not to pressure children who did not want to consume breakfast, but to provide these children with a healthy, nutrient- and energy-dense mid-morning snack.
Mid-morning snack	Children were advised to make healthy snack choices and mothers were encouraged to give children homemade snacks to take with them to school according to their personalized dietary plan.
Lunch	Mothers were advised to provide homemade mediterranean-based meals to their children according to their personalized dietary plan and children were advised to eat until full.
Afternoon snack	Mothers were advised to limit access to energy-dense, nutrient-poor packaged snacks, set clear rules about snacking, but at the same time take into consideration the child’s likes and dislikes and offer them choices according to their personalized dietary plans. Children were advised to avoid excessive snacking.
Dinner	Mothers were advised to prepare healthy, homemade meals for dinner according to the child’s personalized dietary plan. Children were advised to participate as often as possible in the preparation of homemade easy-to-prepare meals and to avoid systematic consumption of fast foods.

**Table 2:** Baseline characteristics.

Characteristics	Pre-Pilot Study (*n* = 20)	Pilot Study Control Group (*n* = 15)	Pilot Study Intervention Group (*n* = 15)	* *p* Value (Between Pilot Groups)
Mean Follow-up Duration (Baseline to the last visit, months)	5.19 (0.66)	4.74 (1.41)	4.04 (0.71)	0.402
Age (years)	10.94 (1.85)	11.11 (1.98)	9.27 (1.73)	0.012
Sex (Female)	8 (40.0)	8 (53.3)	10 (66.7)	0.709
Pubertal Stage (Prepubertal)	8 (40.0)	4 (26.7)	9 (60.0)	0.141
Weight (kg)	76.94 (22.48)	72.19 (23.11)	57.97 (18.11)	0.074
Height (m)	1.51 (0.13)	1.53 (0.14)	1.42 (0.12)	0.029
BMI (kg/m^2^)	33.02 (6.51)	30.11 (5.77)	28.02 (5.37)	0.325
BMI z-score	2.85 (2.57, 4.38)	2.71 (1.97, 3.23)	2.89 (1.91, 4.18)	0.806
Weight Status				0.597
Overweight	-	2 (13.3)	2 (13.3)	
Simple Obesity	11 (55.0)	8 (53.3)	6 (40.0)	
Severe Obesity	9 (45.0)	5 (33.3)	7 (46.7)	
Waist-to-Height Ratio	0.64 (0.08)	0.64 (0.09)	0.60 (0.07)	0.250
Systolic BP (mm Hg)	111.40 (12.75)	116.93 (5.48)	113.33 (10.60)	0.253
Diastolic BP (mm Hg)	73.25 (8.45)	75.10 (8.20)	73.40 (7.68)	0.570
Maternal Age (years)	44.35 (5.08)	44.80 (6.84)	42.40 (3.81)	0.245
Maternal BMI (kg/m^2^)	30.71 (6.35)	29.45 (5.87)	29.65 (6.11)	0.838
Greek Mothers	19 (95.0)	14 (93.3)	15 (100.0)	1.000
Married Mothers	13 (65.0)	13 (86.7)	13 (86.7)	1.000
Maternal Education				0.335
Primary	3 (15.0)	1 (6.7)	-	
Secondary	12 (60.0)	8 (53.3)	7 (46.7)	
Tertiary	5 (25.0)	6 (40.0)	8 (53.3)	
Tertiary				
Employed Mothers	14 (70.0)	12 (80)	13 (86.7)	1.000

Values are expressed as mean (SD) or median (25th and 75th percentiles) for continuous variables and as absolute numbers (n) and frequencies (%) for categorical variables. * Pearson chi square test, Independent *T* test or Mann Whitney-U test between pilot groups.

**Table 3:** Usage metrics of the ENDORSE parental mobile app.

Metric	Pre-Pilot Group (*n* = 18)	Control Group (*n* = 13)	Intervention Group (*n* = 14)
Number of participants with zero usage	3 (16.66%)	0 (0.00%)	0 (0.00%)
Days of usage	19.13 ± 20.66	41.08 ± 36.12	27.64 ± 30.11
Days of weight monitoring	3.47 ± 3.36	7.31 ± 4.53	4.64 ± 3.67
Days of monitoring of goals	20.4 ± 20.42	41.54 ± 35.84	28.64 ± 29.8
Number of communication messages with the clinical team	1.4 ± 2.03	4.23 ± 5.64	0.79 ± 1.42

**Table 4:** Usage metrics of the physical activity tracker.

Metric	Pre-Pilot Group (*n* = 18)	Control Group (*n* = 13)	Intervention Group (*n* = 14)
Number of participants with zero usage during sleep	5 (27.77%)	2 (14.28%)	4 (30.76%)
Number of nights with sleep recordings	29.31 ± 30.43	33.09 ± 28.52	23.00 ± 22.34
Average time of sleep per day (min)	465.02 ± 68.03	477.48 ± 49.22	482.62 ± 67.33
Number of participants with zero usage during day	4 (22.22%)	2 (15.38%)	3 (21.42%)
Average time of usage per day (h)	13.64 ± 5.37	17.86 ± 3.45	14.25 ± 4.37
Number of days with steps recordings	55.64 ± 44.59	51.45 ± 30.73	46.64 ± 33.28
Average steps per day	7446.45 ± 3939.25	9090.63 ± 1197.41	7102.24 ± 2960.17

**Table 5:** Usage metrics of the ENDORSE game.

Metric	Pre-Pilot Group *n* = 18	Intervention Group *n* = 14
Number of participants with zero usage	4 (22.22%)	0 (0.00%)
Days of usage	3.86 ± 3.96	14.57 ± 8.93
Number of action mini-games	4.71 ± 6.65	16.43 ± 9.87
Number of educational mini-games	8.21 ± 7.35	9.79 ± 7.09

**Table 6:** Participants’ responses to the self-report 5-point Likert scale postintervention questionnaire.

Module		Pre-Pilot Group *n* = 15	Intervention Group *n* = 14	*p*-Value
ENDORSE parental mobile app	Helpfulness	3.60 ± 1.02	3.96 ± 0.82	**0.039**
	Usefulness	4.01 ± 1.10	4.11 ± 0.85	0.470
	Ease of Use	3.98 ± 1.16	4.45 ± 0.80	**0.015**
Physical activity tracker	Helpfulness	2.51 ± 1.25	3.73 ± 0.81	**0.000**
	Usefulness	3.09 ± 1.26	3.87 ± 0.99	**0.000**
	Ease of Use	2.85 ± 1.37	3.99 ± 0.92	**0.000**
ENDORSE mobile game	Helpfulness	4.13 ± 0.81	4.29 ± 0.96	0.658
	Usefulness	4.13 ± 0.81	4.14 ± 0.91	0.977
	Ease of Use	4.20 ± 0.83	4.29 ± 0.70	0.775

## Data Availability

The data presented in this study are available on request from the corresponding author after approval of the clinical team. This data are not publicly available due to privacy restrictions.

## Supplementary Materials

The following supporting information can be downloaded at: https://www.mdpi.com/article/10.3390/nu15061451/s1, Post-intervention feasibility questionnaire; Health behaviors questionnaire; Educational material (in Greek).
