# Supplementary material for: The ENDORSE Feasibility Study: Exploring the Use of M-Health, Artificial Intelligence and Serious Games for the Management of Childhood Obesity
Source: Nutrients. 2023 Mar 17;15(6):1451. doi: 10.3390/nu15061451 (PMC10057317; doi:10.3390/nu15061451)
Supplement: Supplementary file 1 [file nutrients-15-01451-s001.zip › Educational material.pdf]

Α' Παιδιατρική Κλινική
Πανεπιστημίου Αθηνών

ΕΙΔΙΚΗ ΜΟΝΑΔΑ ΠΑΙΔΙΚΗΣ ΠΑΧΥΣΑΡΚΙΑΣ

Εκπαιδευτικό Υλικό Για Τη Ρύθμιση Βάρους Παιδιών Και Εφήβων

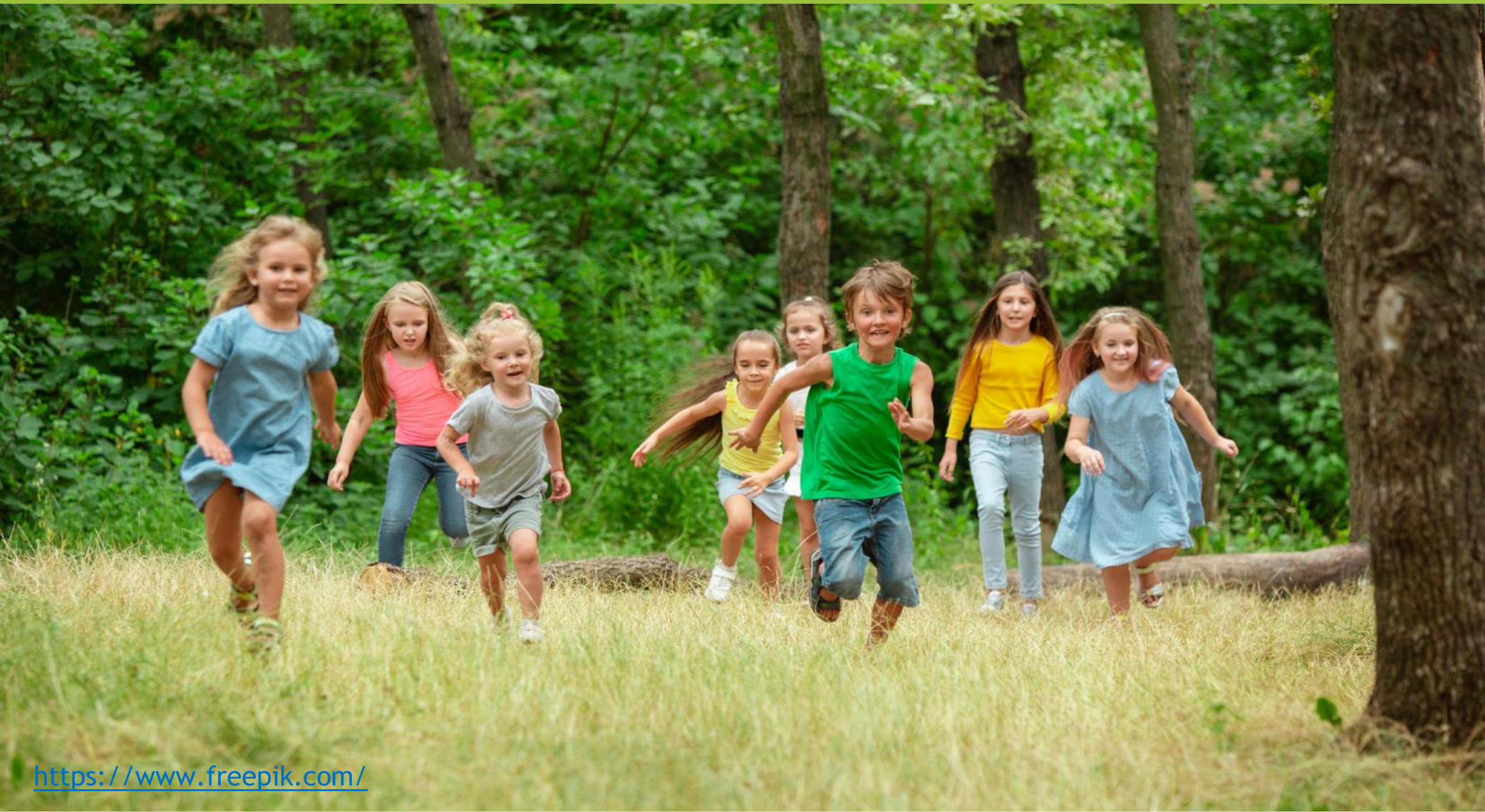

https://www.freepik.com/

Α’ ΠΑΙΔΙΑΤΡΙΚΗ ΚΛΙΝΙΚΗ ΠΑΝΕΠΙΣΤΗΜΙΟΥ ΑΘΗΝΩΝ

ΕΙΔΙΚΗ ΜΟΝΑΔΑ ΠΑΙΔΙΚΗΣ ΠΑΧΥΣΑΡΚΙΑΣ

ΕΚΠΑΙΔΕΥΤΙΚΟ ΥΛΙΚΟ ΓΙΑ ΤΗ ΡΥΘΜΙΣΗ ΒΑΡΟΥΣ ΠΑΙΔΙΩΝ ΚΑΙ ΕΦΗΒΩΝ

Επιστημονική ομάδα

Κανακά - Gantenbein Χριστίνα, Καθηγήτρια Παιδιατρικής - Παιδιατρικής Ενδοκρινολογίας, Διευθύντρια της Α’ Παιδιατρικής Πανεπιστημιακής Κλινικής Ιατρικής Σχολής Εθνικού και Καποδιστριακού Πανεπιστημίου Αθηνών, Νοσοκομείο Παίδων «Η Αγία Σοφία»

Περβανίδου Νένη, Αναπληρώτρια Καθηγήτρια Αναπτυξιακής και Συμπεριφορικής Παιδιατρικής, Υπεύθυνη της Μονάδας Αναπτυξιακής και Συμπεριφορικής Παιδιατρικής της Α’ Παιδιατρικής Πανεπιστημιακής Κλινικής Ιατρικής Σχολής Εθνικού και Καποδιστριακού Πανεπιστημίου Αθηνών, Νοσοκομείο Παίδων «Η Αγία Σοφία»

Πολυχρονάκη Νεκταρία, Διαιτολόγος-Διατροφολόγος, Διδάκτωρ Κλινικής Διατροφής Ιατρικής Σχολής Πανεπιστημίου Κουόπιο Φινλανδίας, Επιστημονική Συνεργάτης της Ειδικής Μονάδας Παιδικής Παχυσαρκίας της Α’ Παιδιατρικής Πανεπιστημιακής Κλινικής της Ιατρικής Σχολής Εθνι- κού και Καποδιστριακού Πανεπιστημίου Αθηνών, Νοσοκομείο Παίδων «Η Αγία Σοφία»

Χατζηδάκη Εύη, Παιδίατρος, PhD, Msc στην Κλινική Διατροφή (Τμήμα Διατροφής -Χαροκόπειο Πανεπιστήμιο), Επιστημονική Συνεργάτης της Ειδικής Μονάδας Παιδικής Παχυσαρκίας της Α’ Παιδιατρικής Πανεπιστημιακής Κλινικής της Ιατρικής Σχολής Εθνικού και Καποδιστριακού Πανεπιστημίου Αθηνών, Νοσοκομείο Παίδων «Η Αγία Σοφία»

Το παρόν εκπαιδευτικό υλικό δημιουργήθηκε στα πλαίσια συμμετοχής της Ειδικής Μονάδας Παιδικής Παχυσαρκίας της Α’ Παιδιατρικής Πανεπιστημιακής Κλινικής της Ιατρικής Σχολής Εθνι- κού και Καποδιστριακού Πανεπιστημίου Αθηνών (ΦΕΚ 1856/τεύχος 2/29 Δεκεμβρίου 2005) στο πρόγραμμα ENDORSE: «Ολοκληρωμένη προσέγγιση προαγωγής αυτοδιαχείρισης της υγείας σε παιδιά και εφήβους με Σακχαρώδη Διαβήτη Τύπου 1 ή / και με παχυσαρκία βασισμένη σε μηχανισμούς παιχνιδοποίησης και ανάδρασης», του Ερευνητικού Πανεπιστημιακού Ινστιτούτου Συστημάτων Επικοινωνιών και Υπολογιστών (ΕΠΙΣΕΥ), το οποίο χρηματοδοτείται στο πλαίσιο του Επιχειρησιακού Προγράμματος «Ανταγωνιστικότητα, Επιχειρηματικότητα και Καινοτομία (ΕΠΑνΕΚ)», ΕΣΠΑ 2014-2020.

Εισαγωγη

Το παρόν εκπαιδευτικό υλικό απευθύνεται στους γονείς και κηδεμόνες των παιδιών και των εφήβων που προσέρχονται στο ιατρείο με σκοπό την επιστημονικά τεκμηριωμένη ενημέρωσή τους σε θέματα που αφορούν στη ρύθμιση του βάρους στην παιδική και εφηβική ηλικία. Οι γονείς με τη σειρά τους μπορούν να χρησιμοποιήσουν το παρόν υλικό ως οδηγό για να εκπαιδεύσουν και να συζητήσουν με τα παιδιά τους τις αρχές της υγιεινής διατροφής με βάση το πρότυπο της Ελληνικής Μεσογειακής Διατροφής, αλλά και τις αρχές του ενεργειακού ισοζυγίου με σκοπό την αυτορρύθμιση και αυτονομία των παιδιών και των εφήβων σε θέματα διατροφής και σωματικής δραστηριότητας. Βασική βιβλιογραφική πηγή αποτελεί ο Εθνικός Διατροφικός Οδηγός Βρεφών, Παιδιών και Εφήβων του Υπουργείου Υγείας και Πρόνοιας, αλλά και μελέτες από τη διεθνή βιβλιογραφία που αφορούν στο ενεργειακό ισοζύγιο, στην υγιεινή διατροφή, στη διαμόρφωση της διατροφικής συμπεριφοράς ανάλογα με το αναπτυξιακό στάδιο των παιδιών και τις πρακτικές σίτισης που χρησιμοποιούν οι γονείς και σχετίζονται με καλύτερη ρύθμιση του βάρους των παιδιών.

Στόχος είναι να αποτελέσει το παρόν εκπαιδευτικό υλικό ένα χρήσιμο συμπληρωματικό εργαλείο, το οποίο μαζί με το εξατομικευμένο διατροφικό πλάνο που λαμβάνει κάθε παιδί που επισκέπτεται το ιατρείο, να βοηθήσει στην επίτευξη των στόχων που έχουν τεθεί για κάθε παιδί όσον αφορά το βάρος του.

Ενεργειακο Ισοζυγιο

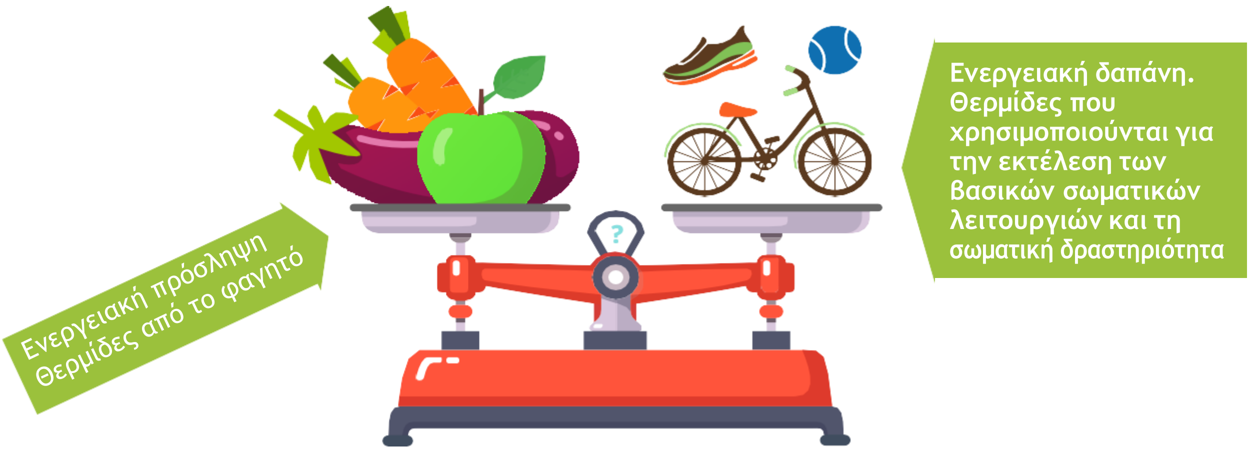

https://www.freepik.com/

Το βάρος μας αυξάνεται όταν η ενεργειακή μας πρόσληψη (κατανάλωση τροφής) ξεπερνά την ενεργειακή μας δαπάνη (ενέργεια που καταναλώνεται για την εκτέλεση βασικών λειτουργιών του σώματος και τη σωματική δραστηριότητα). Στα παιδιά και στους εφήβους περιλαμβάνεται και η ενέργεια που απαιτείται για την αύξηση του σώματος. Οι περισσότεροι ερευνητές που ασχολούνται με την ρύθμιση του βάρους καταλήγουν στο συμπέρασμα ότι > 90% των περιπτώσεων παχυσαρκίας είναι αποτέλεσμα διαταραχής στο ενεργειακό ισοζύγιο (μειωμένη σωματική δραστηριότητα σε συνδυασμό με υπερβολική λήψη τροφής) σε γενετικά προδιατεθειμένα άτομα. Ως τώρα έχουν αναγνωριστεί πάνω από 200 γονίδια που σχετίζονται με αυξημένο βάρος.

Τα συνηθέστερα γονίδια που ενοχοποιούνται για την προδιάθεση αύξησης βάρους εμπλέκονται είτε στο βαθμό ανταπόκρισης στο αίσθημα του κορεσμού (satiety responsiveness - δηλαδή οι άνθρωποι αυτοί δε χορταίνουν εύκολα), είτε σχετίζονται με υψηλό βαθμό ανταπόκρισης στην έκθεση συγκεκριμένων εύγευστων τροφίμων (food responsiveness - π.χ. όταν βλέπω το αγαπημένο μου σνακ αισθάνομαι ότι δεν μπορώ να αντισταθώ και θέλω να το καταναλώσω άμεσα). Στο σύγχρονο τρόπο ζωής μας (καθιστική ζωή, νόστιμα, φθηνά, γεμάτα ενέργεια τρόφι- μα) αρκετοί άνθρωποι (ενήλικες και παιδιά) δυσκολεύονται να ρυθμίσουν το βάρος τους εντός φυσιολογικών ορίων. Για τα παιδιά η καθημερινή σωματική δραστηριότητα (π.χ. περπάτημα, ποδήλατο, οργανωμένη άσκηση κ.ά.) σε συνδυασμό με τη μεσογειακή διατροφή αποτελεί τον καλύτερο τρόπο να ρυθμίσουν το βάρος τους εντός φυσιολογικών ορίων.

Οι περισσότεροι διεθνείς οργανισμοί που εκδίδουν κατευθυντήριες οδηγίες για την παιδική υπερβαρότητα και παχυσαρκία μεταξύ αυτών και ο Παγκόσμιος Οργανισμός Υγείας και η Αμερικάνικη Ενδοκρινολογική Εταιρεία (Endocrine Society) τονίζουν την ιδιαίτερη σημασία της υγιεινής διατροφής, της άσκησης, της μείωσης της καθιστικής ζωής και του επαρκούς ύπνου τόσο στην πρόληψη όσο και στην αντιμετώπιση της παιδικής παχυσαρκίας.

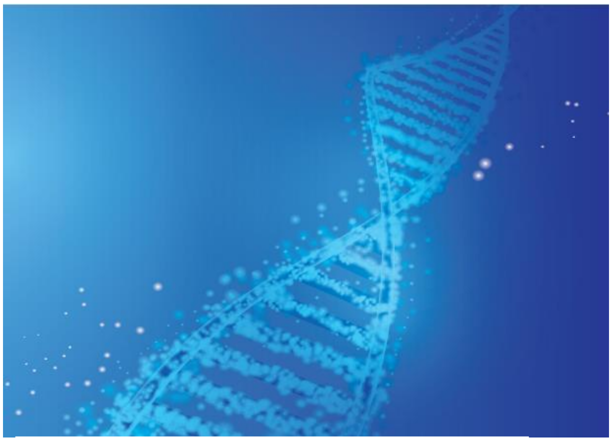

https://www.freepik.com/

Ενεργειακη Προσληψη

Η ενεργειακή μας πρόσληψη γίνεται με την κατανάλωση τροφής. Ενέργεια μας δίνουν τα λεγόμενα μακροθρεπτικά συστατικά της τροφής, δηλαδή οι υδατάνθρακες (π.χ. δημητριακά, όσπρια και λιγότερο τα φρούτα και τα λαχανικά), τα λίπη και τα έλαια (π.χ. ελαιόλαδο και τα άλλα φυτικά έλαια, ξηροί καρποί, βούτυρο, ζωϊκό λίπος) και οι πρωτεΐνες (ζωϊκής προέλευσης όπως το κοτόπουλο, το μοσχάρι, τα ψάρια, τα αυγά αλλά και φυτικής προέλευσης όπως οι πρωτεΐνες των οσπρίων). Για τις ενεργειακές του ανάγκες ο οργανισμός μας χρησιμοποιεί κυρίως τους υδατάνθρακες και τα λίπη, ενώ οι πρωτεΐνες έχουν κυρίως δομικό ρόλο. Η μεσογειακή διατροφή αποτελεί ένα παγκόσμιο πρότυπο υγιεινής διατροφής με κύρια χαρακτηριστικά την χρήση του εξαιρετικού παρθένου ελαιόλαδου ως κύριο προστιθέμενο έλαιο στη μαγειρική, την υψηλή κατανάλωση προϊόντων φυτικής προέλευσης, όπως φρούτα, λαχανικά, αδρά επεξεργασμένα δημητριακά και όσπρια, τη μέτρια προς υψηλή κατανάλωση ψαριού και θαλασσινών, τη μέτρια κατανάλωση γαλακτοκομικών, πουλερικών και αυγών και τη χαμηλή κατανάλωση κόκκινου κρέατος (π.χ. μοσχάρι, χοιρινό) και προϊόντων του (π.χ. αλλαντικών).

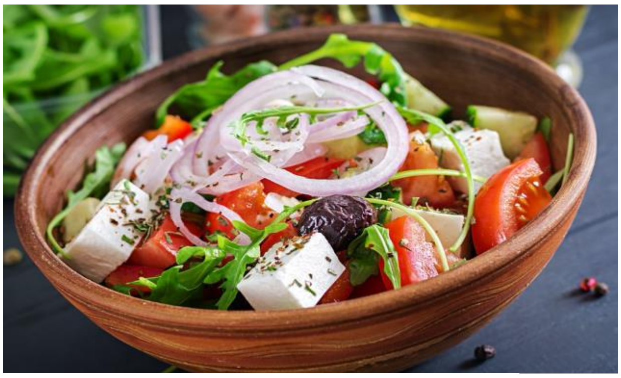

https://www.freepik.com/

Όσον αφορά τις διατροφικές συνήθειες των παιδιών που επηρεάζουν το βάρος τους, έμφαση δίνεται από την Endocrine Society στην αποφυγή των πλούσιων σε θερμίδες, αλλά χαμηλών σε θρεπτικά συστατικά τροφίμων (calorie-dense, nutrient poor foods) όπως τα αναψυκτικά με απλά σάκχαρα (sugar sweetened beverages - SSB), χυμοί εμπορίου, φαγητά ταχυφαγείου (fast foods), επεξεργασμένα προϊόντα πλούσια σε απλά σάκχαρα, αλάτι και πρόσθετα λίπη και στην ενθάρρυνση κατανάλωσης ολόκληρων φρούτων αντί για χυμούς. Η μεσογειακή διατροφή περιλαμβάνει πολλές νόστιμες και υγιεινές επιλογές (π.χ. σπιτικό κέικ με ελαιόλαδο, σπιτική τυρόπιτα κ.ά.), οι οποίες μπορούν να περιορίσουν την κατανάλωση τυποποιημένων προϊόντων, προσφέροντας γεύση, ενέργεια και τα απαραίτητα θρεπτικά συστατικά που χρειάζονται τα παιδιά για τη σωματική και πνευματική τους ανάπτυξη.

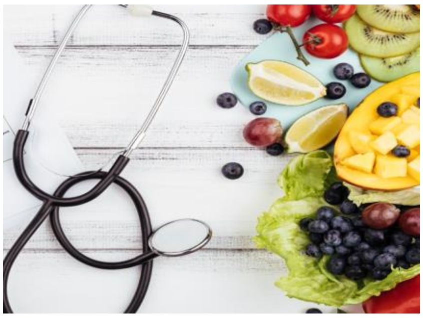

https://www.freepik.com/

Σημαντικό για τη ρύθμιση του βάρους των παιδιών είναι, όπως θα δούμε και παρακάτω, όχι μόνο το τι τρώνε τα παιδιά (ποιότητα του συνολικού τους διατροφικού προτύπου), αλλά και το πόσο τρώνε (π.χ. κατάλληλες μερίδες φαγητού ανάλογα με την ηλικία, το βάρος και τη σωματική δραστηριότητα του παιδιού) και το πώς τρώνε (π.χ. γρήγορα ή αργά, σταθερά γεύματα ή ακατάστατα κ.ά.)

Ενεργειακη Δαπανη

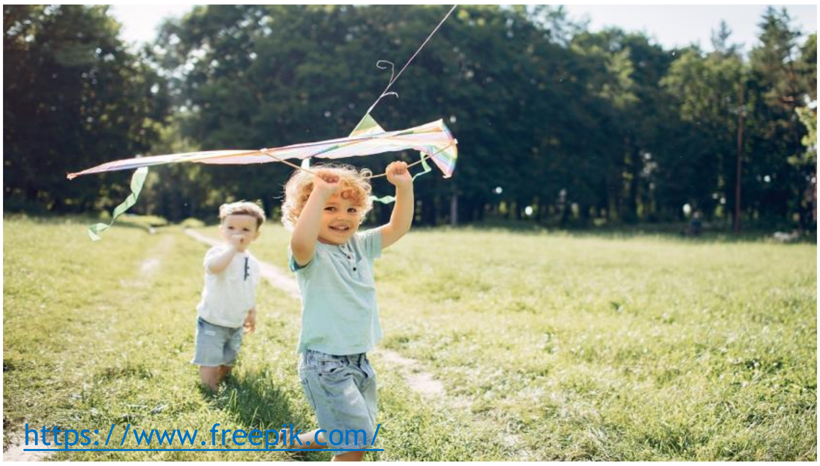

Σωματική δραστηριότητα αποτελεί κάθε κίνηση του σώματος για την οποία απαιτείται κατανάλωση ενέργειας (π.χ. περπάτημα, ανέβασμα σκάλας, οικιακές εργασίες, άσκηση). Αποτελεί σημαντικό παράγοντα για τη διατήρηση φυσιολογικού σωματικού βάρους. Σε αντίθεση, η άσκηση αποτελεί ειδική μορφή σωματικής δραστηριότητας, η οποία είναι προγραμματισμένη, δομημένη, επαναλαμβανόμενη, και αποσκοπεί στη βελτίωση ή διατήρηση της φυσικής κατάστασης. Περιλαμβάνει όλα τα αθλήματα, τη γυμναστική, τον χορό κ.ά. Τα οφέλη της τακτικής σωματικής δραστηριότητας είναι πολλαπλά: καλύτερη ρύθμιση του σωματικού βάρους, συμβολή στη σωστή ανάπτυξη του σώματος (γερά κόκαλα, δυνατοί μύες), πρόληψη καρδιομεταβολικών νοσημάτων ενηλίκων (π.χ. υπέρταση, διαβήτης), ενίσχυση της αυτοπεποίθησης, της κοινωνικής αλληλεπίδρασης και της ομαδικότητας και βελτίωση των γνωστικών λειτουργιών.

Η σύσταση των παγκόσμιων οργανισμών Υγείας (WHO, Endocrine Society, Αμερικάνικη Παιδιατρική Εταιρεία) για τα παιδιά και τους εφήβους είναι καθημερινή σωματική δραστηριότητα μέτριας ή υψηλής έντασης διάρκειας τουλάχιστον 1 ώρας (συνεχόμενη ή μπορεί να μοιράζεται σε μικρότερα διαστήματα τουλάχιστον 10 λεπτών).

Μέτριας έντασης θεωρούνται οι δραστηριότητες που κάνουν την καρδιά, τους πνεύμονες και τους μυς να λειτουργήσουν πιο έντονα από ότι συνήθως, προκαλώντας εμφανή αύξηση των καρδιακών παλμών και του ρυθμού αναπνοής, π.χ. έντονο περπάτημα, ανέβασμα σκάλας, χορός, κολύμπι, δουλειές σπιτιού. Κατά τη διάρκεια δραστηριότητας μέτριας έντασης το παιδί μπορεί να μιλήσει αλλά όχι να τραγουδήσει!

Υψηλής έντασης θεωρούνται οι δραστηριότητες που κάνουν την καρδιά, τους πνεύμονες και

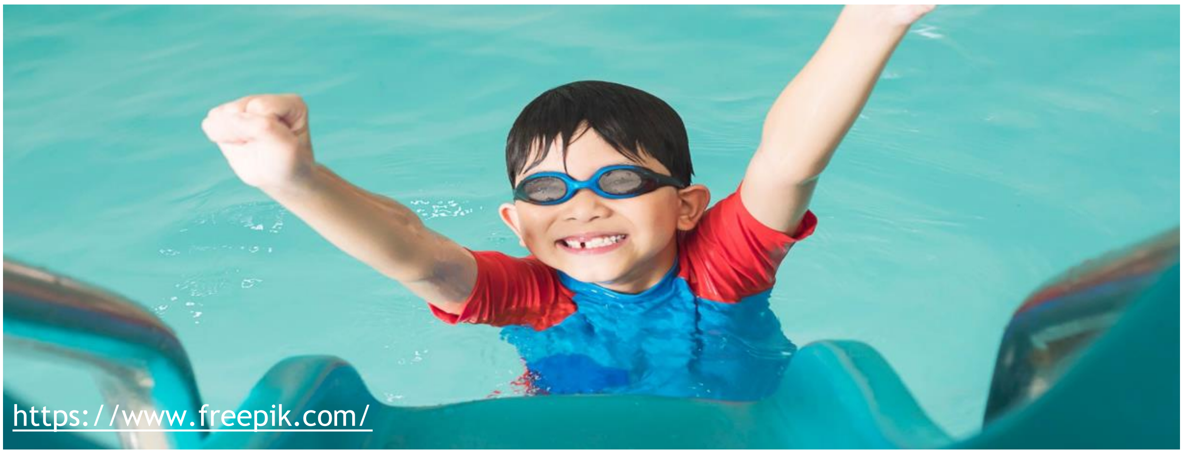

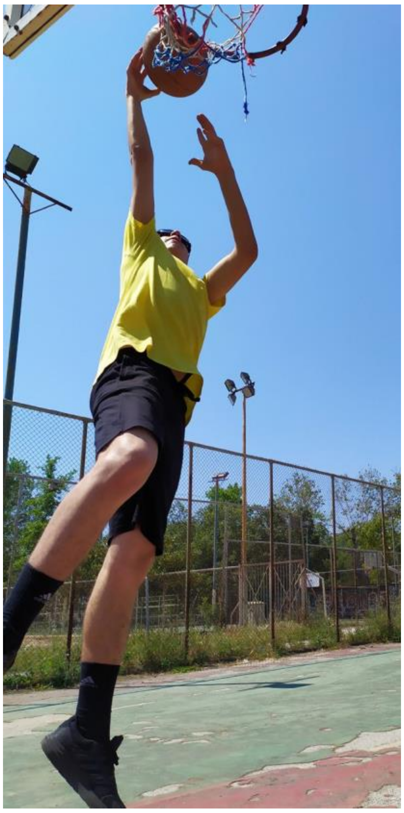

τους μυς να λειτουργήσουν ακόμη πιο έντονα σε σχέση με τις δραστηριότητες μέτριας έντασης, αυξάνοντας κατά πολύ τους καρδιακούς παλμούς και τον ρυθμό αναπνοής, π.χ. τρέξιμο, ποδηλασία με γρήγορους ρυθμούς, γρήγορο κολύμπι, μπάσκετ, ποδόσφαιρο. Κατά τη διάρκεια δραστηριότητας υψηλής έντασης το παιδί δεν μπορεί να πει παρά λίγες λέξεις πριν σταματήσει για να πάρει ανάσα!

Εκτός από τη διατροφή και τη σωματική δραστηριότητα, η καθιστική ζωή και ο ύπνος έχουν συσχετιστεί στατιστικά με την εμφάνιση παιδικής παχυσαρκίας σε πολλές μελέτες από τη διεθνή βιβλιογραφία και αποτελούν στόχους παρέμβασης για την αντιμετώπιση του υπερβάλλοντος βάρους σε παιδιά και εφήβους.

Σύμφωνα με τους περισσότερους διεθνείς Οργανισμούς, μεταξύ αυτών και η Αμερικάνικη Ενδοκρινολογική Εταιρεία (Endocrine Society) συστήνεται για τα παιδιά και τους εφήβους ο χρόνος που δαπανάται μπροστά σε οθόνες (π.χ. τηλεόραση, τάμπλετ, κινητά κ.ά.) για λόγους ψυχαγωγίας να μην ξεπερνά τις μια με δύο ώρες ημερησίως, ενώ σύμφωνα με το Εθνικό Ίδρυμα Ύπνου των ΗΠΑ (National Sleep Foundation) η συνολική ημερήσια διάρκεια ύπνου των παιδιών συστήνεται να είναι 9 με 11 ώρες (παιδιά ηλικίας 6 έως 13 ετών) και 8 με 10 ώρες (έφηβοι ηλικίας 14 - 17 ετών).

Στοχοι Για Την Απωλεια Βαρους Σε Υπερβαρα Παιδια Και Εφηβους

Η μεγάλη αύξηση της παιδικής και εφηβικής παχυσαρκίας τις τελευταίες δεκαετίες έχει οδηγήσει πολλούς Οργανισμούς Υγείας όπως η Αμερικάνικη Ενδοκρινολογική Εταιρεία (Endocrine Society) και η Αμερικάνικη Παιδιατρική Εταιρία (American Academy of Pediatrics - AAP) να εκδώσουν κατευθυντήριες οδηγίες για την καλύτερη διαχείριση αυτού του σημαντικού δημόσιου προβλήματος υγείας.

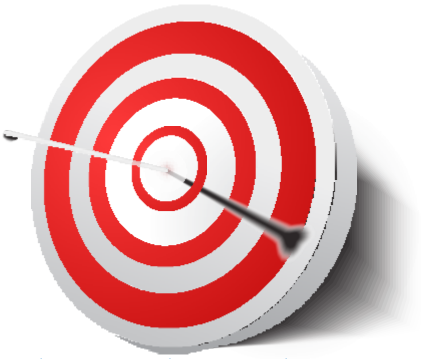

https://pixabay.com/el/images/search/

Σύμφωνα με τη Διεθνή Ομάδα για την αντιμετώπιση της παχυσαρκίας (International Obesity Task Force - IOTF) ως υπέρβαρα χαρακτηρίζονται τα παιδιά των οποίων ο Δείκτης Μάζας Σώματος (ΔΜΣ) είναι μεγαλύτερος από την 85η ΕΘ για το φύλο και την ηλικία τους, ενώ ως παχύσαρκα χαρακτηρίζονται τα παιδιά των οποίων ο ΔΜΣ είναι μεγαλύτερος από την 95η ΕΘ για το φύλο και την ηλικία. Στην πρώτη γραμμή αντιμετώπισης των υπέρβαρων παιδιών βρίσκεται η αλλαγή στον τρόπο ζωής, με πιο αποτελεσματικές τις παρεμβάσεις που γίνονται σε πολλαπλά επίπεδα (multicomponent interventions) και απευθύνονται σε όλη την οικογένεια, οι οποίες περιλαμβάνουν διατροφική παρέμβαση, άσκηση, συμπεριφορικές τεχνικές και εκπαίδευση των παιδιών και των γονέων σε θέματα συμπεριφορών που σχετίζονται με την υγεία.

Η Αμερικάνικη Παιδιατρική Εταιρεία το 2007 εξέδωσε κατευθυντήριες οδηγίες που απευθύνονται σε παιδιάτρους για την αντιμετώπιση της παιδικής υπερβαρότητας και παχυσαρκίας, οι οποίες συστάσεις παραμένουν ίδιες έως και σήμερα. Για όλα τα παιδιά των οποίων ο ΔΜΣ είναι μεγαλύτερος από την 85η ΕΘ ή την 95η ΕΘ συστήνεται για διάστημα 3 έως 6 μηνών να δοθεί έμφαση σε στρατηγικές πρόληψης (στάδιο prevention plus, βλέπε πίνακα 1), έτσι ώστε να υπάρχει μια σταδιακή μείωση του ΔΜΣ. Λόγω της αύξησης του ύψους των παιδιών ο ΔΜΣ μπορεί να μειωθεί μέσα σε 3 με 6 μήνες ακόμη και με διατήρηση του βάρους σταθερού ή με μικρή μείωση του βάρους έως 0,5 – 1 κιλό το μήνα. Για παιδιά με ΔΜΣ μεγαλύτερο από την 95η ΕΘ και εφόσον παρέλθει το διάστημα αυτό και δεν έχει επιτευχθεί η μείωση του ΔΜΣ, συστήνεται από την Αμερικάνικη Παιδιατρική Εταιρεία μια πιο εντατική παρέμβαση κατά προτίμηση από ομάδα ειδικών (π.χ. παιδοδιατροφολόγος, εξειδικευμένος παιδίατρος), όπου, εκτός από την έμφαση στις

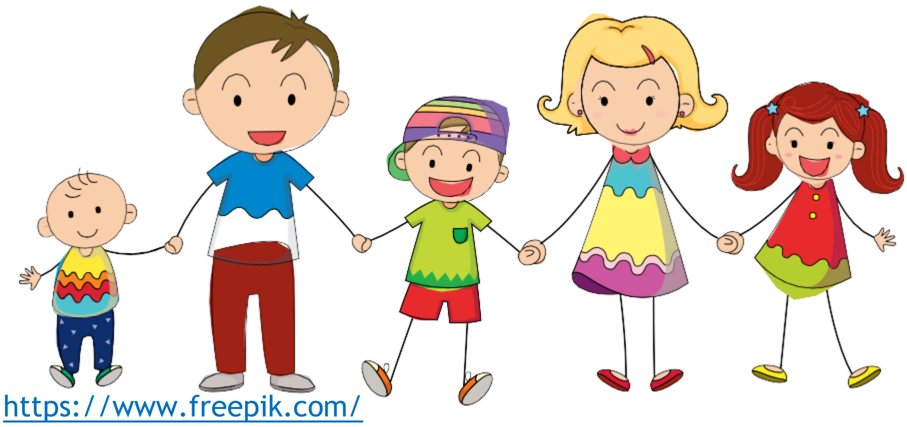

https://www.freepik.com/

προαναφερθείσες στρατηγικές πρόληψης, δίδεται στο παιδί και ένα εξατομικευμένο διατροφικό πλάνο και η παρακολούθηση του παιδιού είναι πιο συστηματική (στάδιο 2 ή structured weight management). Οι στόχοι για την απώλεια βάρους και σε αυτό το στάδιο παραμένουν οι ίδιοι, δηλαδή απώλεια βάρους έως 0,5 - 1 κιλό το μήνα.

Στο ιατρείο ακολουθούμε τις παραπάνω συστάσεις για σταδιακή απώλεια βάρους και μόνο σε εξαιρετικές περιπτώσεις, κατόπιν παιδοενδοκρινολογικής σύστασης (π.χ. σε παιδιά ή εφήβους με επιπλοκές που σχετίζονται με την παχυσαρκία όπως προδιαβήτης ή υπέρταση) συστήνουμε μια ταχύτερη απώλεια βάρους μέσω τήρησης αυστηρού διαιτολογίου και υπό την προϋπόθεση ότι η οικογένεια του παιδιού είναι κινητοποιημένη και το παιδί ή ο έφηβος θα παρακολουθείται ανά βδομάδα στο ιατρείο για όσο χρονικό διάστημα βρίσκεται σε υποθερμιδική δίαιτα (συνήθης διάρκεια παρέμβασης 8 βδομάδες).

**Πίνακας 1:** Στόχοι του σταδίου I (Prevention plus) για την αντιμετώπιση της παιδικής υπερβαρότητας σύμφωνα με τις οδηγίες της Αμερικάνικης Παιδιατρικής Εταιρείας
≥ 5 μερίδες φρούτα και λαχανικά καθημερινά
Περιορισμός σακχαρούχων αναψυκτικών
Μείωση της τηλεθέασης ≤ 2 ώρες καθημερινά
Αύξηση της φυσικής δραστηριότητας ≥ 1 ώρα καθημερινά
Γεύματα στο σπίτι όσο πιο συχνά γίνεται
Κατανάλωση γευμάτων από όλη την οικογένεια μαζί
Καθημερινή κατανάλωση υγιεινού πρωινού
Συμμετοχή ολόκληρης της οικογένειας στις αλλαγές του τρόπου ζωής
Αυτορρύθμιση της ποσότητας στα γεύματα από το παιδί και αποφυγή περιοριστικής συμπεριφοράς από τους γονείς του
Εξατομίκευση και προσαρμογή των συστάσεων βάσει της τοπικής κουλτούρας και των χαρακτηριστικών της οικογένειας

ΤΑ ΓΕΥΜΑΤΑ ΜΟΥ: ΠΟΤΕ, ΠΟΣΟ ΚΑΙ ΤΙ

Στην ενότητα που ακολουθεί θα μάθουμε τα κυριότερα θέματα που αφορούν στη Μεσογειακή Διατροφή για παιδιά και εφήβους, έτσι όπως περιγράφονται αναλυτικά στον Εθνικό Διατροφικό Οδηγό για παιδιά και εφήβους του Υπουργείου Υγείας και Πρόνοιας. Η **συχνότητα** των γευμάτων, οι **μερίδες** των τροφίμων και η **ποιότητα** των τροφών είναι όλα πολύ σημαντικά για την υγεία, την ευεξία και το σωματικό βάρος των παιδιών μας.

Ο «ΔΕΚΑΛΟΓΟΣ» ΤΗΣ ΥΓΙΕΙΝΗΣ ΔΙΑΤΡΟΦΗΣ

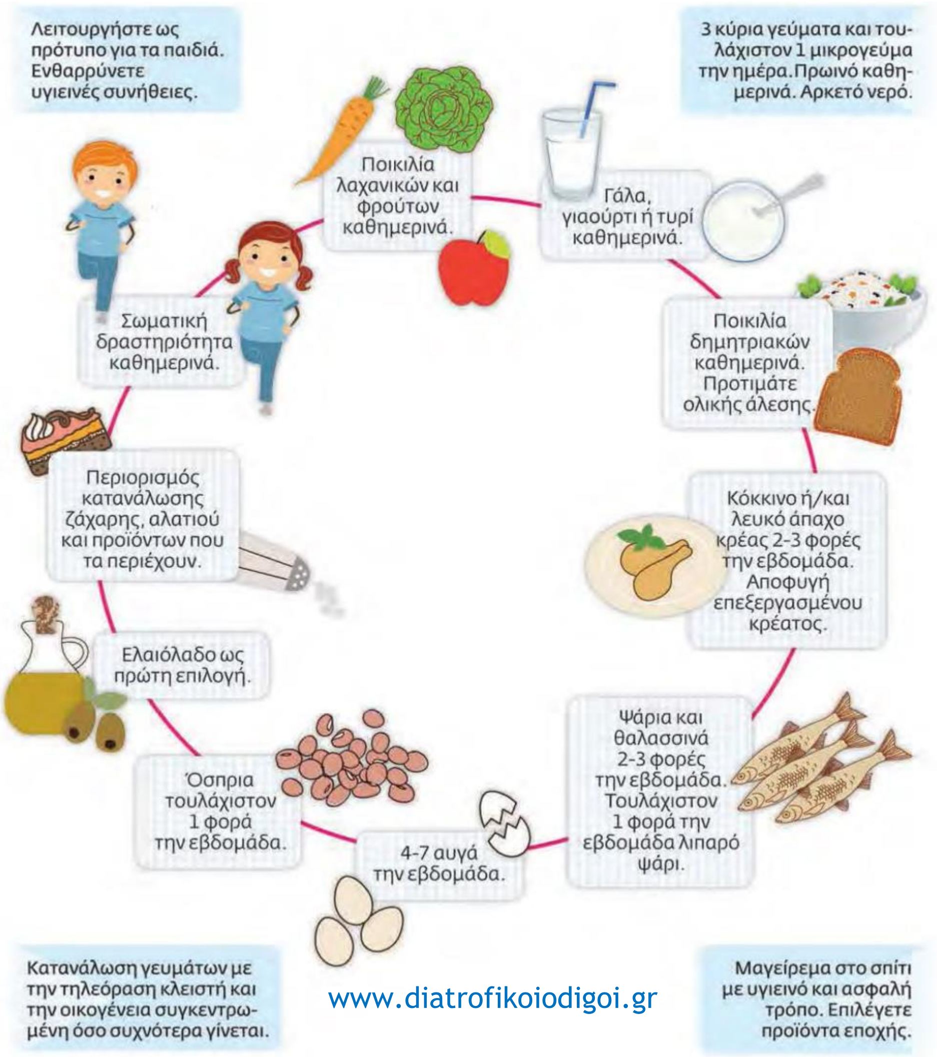

Ο «Δεκάλογος» της Υγιεινής Διατροφής
(σελ. 44 του Εθνικού Διατροφικού Οδηγού για Βρέφη, Παιδιά και Εφήβους)
Ινστιτούτο Προληπτικής Περιβαλλοντικής και Εργασιακής Ιατρικής, Prolepsis

«Πότε;» Συχνότητα και είδος γευμάτων

Καθημερινά τα παιδιά άνω των δύο ετών μπορούν να καταναλώνουν **3 κύρια γεύματα** και τουλάχιστον ένα **ενδιάμεσο μικρογεύμα (σνακ) ως δεκατιανό ή / και απογευματινό** κατά προτίμηση σε περίπου σταθερές ώρες.

ΚΥΡΙΑ ΓΕΥΜΑΤΑ

Τα **κύρια γεύματα** (πρωινό, μεσημεριανό, βραδινό) είναι σημαντικά για την αυτορρύθμιση του παιδιού ή του εφήβου σε σχέση με την πείνα και τον κορεσμό. Σε αυτά τα γεύματα, οι γονείς ή οι φροντιστές έχουν την κύρια ευθύνη για την ποιότητα του γεύματος και την μερίδα που βρίσκεται στο τραπέζι και το παιδί ή ο έφηβος μπορούν ελεύθερα να ρυθμίσουν εάν και πόσο θέλουν να φάνε. Είναι σημαντικό σε αυτά τα γεύματα τα παιδιά / έφηβοι να χορταίνουν ώστε να μην αναζητούν έπειτα να καλύψουν την ανάγκη για φαγητό με θερμιδικά σνακ. Οι γονείς ή οι φροντιστές λειτουργούν ως πρότυπα επηρεάζοντας με το παράδειγμά τους τα παιδιά ως προς τη διατροφική τους συμπεριφορά.

Πρωινό: Το πρωινό είναι ένα σημαντικό γεύμα της ημέρας. Ενθαρρύνουμε τα παιδιά να καταναλώνουν πρωινό χωρίς να τα πιέζουμε. Είναι σημαντικό να επιλέγουμε πρωινό που έχουμε φτιάξει στο σπίτι (π.χ. γάλα + ψωμί ολικής άλεσης + ταχίνι και μέλι, γάλα + βρώμη + φρούτο, μικρό ποτήρι φρέσκο στυμμένο χυμό + σπιτική τυρόπιτα με ελαιόλαδο κ.ά.). Σε περίπτωση που επιλέξουμε ένα συσκευασμένο προϊόν, φροντίζουμε να ελέγξουμε την ετικέτα για να βεβαιωθούμε πως είναι χωρίς πολλά σάκχαρα και υδρογονωμένα λιπαρά (βλέπε κεφ.: Τι να προσέξω στις ετικέτες;).

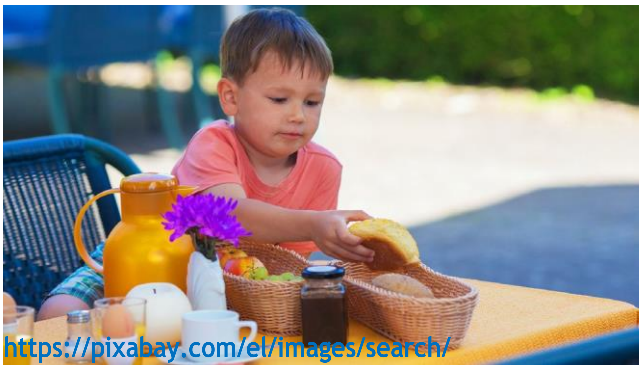
https://pixabay.com/el/images/search/

Μεσημεριανό: Προτιμήστε το σπιτικό μαγειρευτό φαγητό. Μαγειρέψτε μεσογειακά, μόνο με ελαιόλαδο, προσθέστε όπου μπορείτε στις συνταγές σας λαχανικά. Το πιάτο του παιδιού σας χρειάζεται να είναι ισορροπημένο περιλαμβάνοντας τροφές από διάφορες ομάδες τροφίμων όπως στο παρακάτω παράδειγμα.

ΤΟ ΚΥΡΙΟ ΠΙΑΤΟ ΜΟΥ

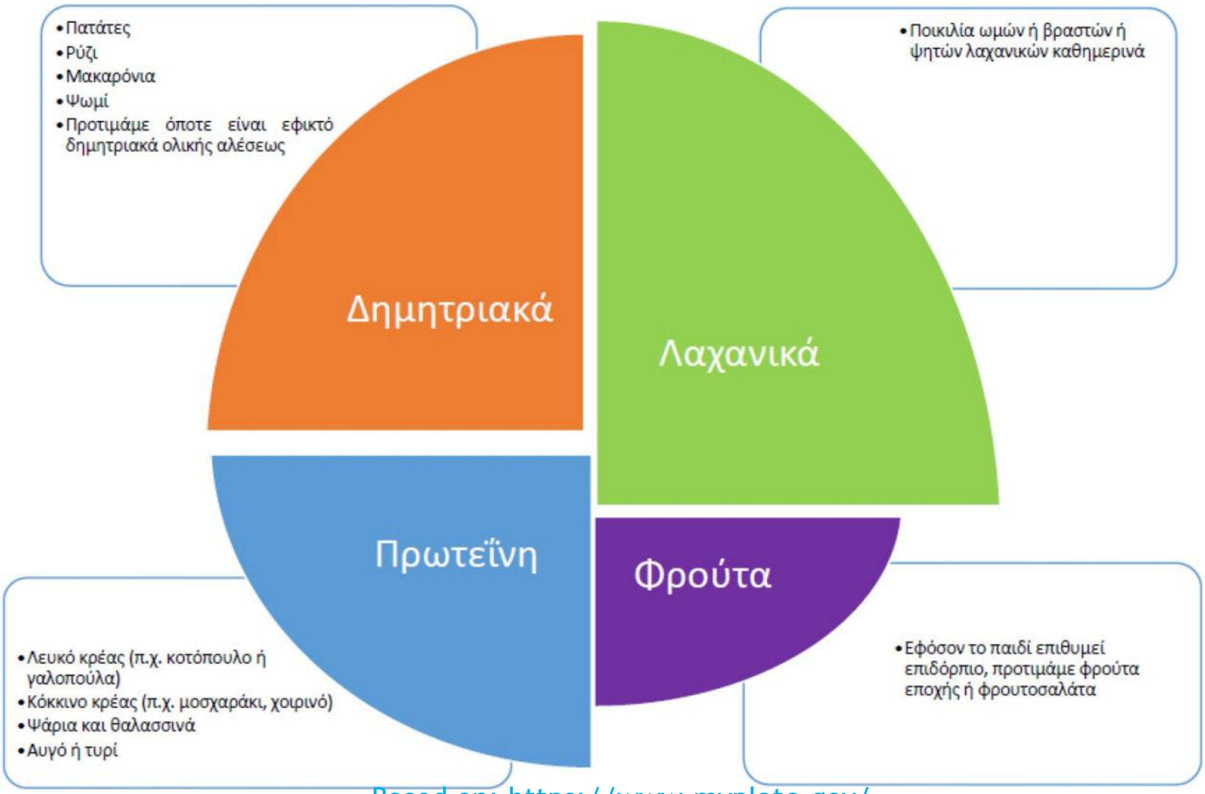

Based on: https://www.myplate.gov/

Όταν το κύριο πιάτο μου είναι λαδερό ή όσπριο, μπορώ να συμπληρώσω το γεύμα με ένα κομμάτι τυρί και μια φέτα ψωμί ή ένα κομμάτι σπιτική πίτα ή ένα αυγό όπως φαίνεται ενδεικτικά στην παρακάτω εικόνα (από τη σελ. 95 του Εθνικού Διατροφικού Οδηγού για Βρέφη, Παιδιά και Εφήβους):

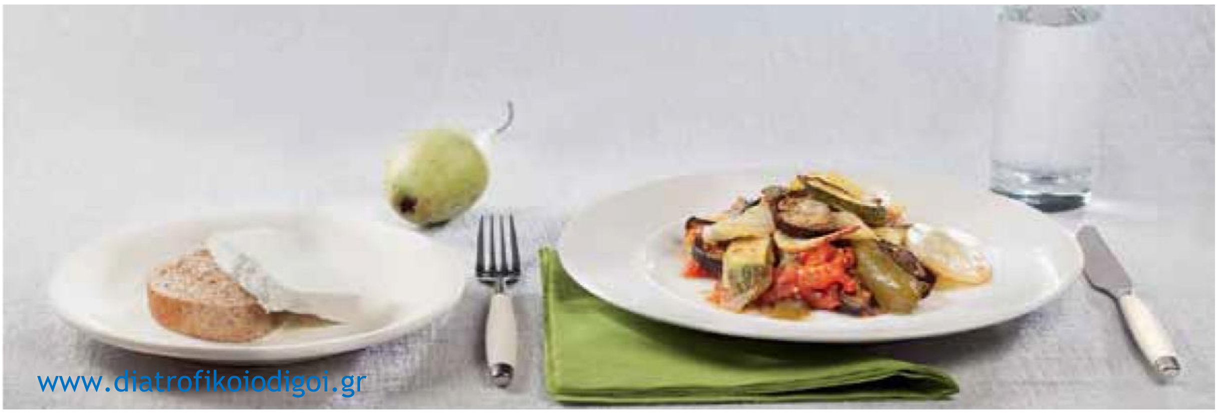

Βραδινό: Για βραδινό γεύμα, προτείνεται η κατανάλωση μαγειρεμένου φαγητού είτε από το μεσημέρι είτε με τη χρήση σπιτικών σύντομων σε χρόνο συνταγών όπως ομελέτα, σπιτικές κοτομπουκιές, σπιτικό σουβλάκι, μπιφτέκι, ντάκος, τονοσαλάτα κ.α. Συνοδέψτε με παξιμάδι ολικής άλεσης ή κρίθινο, με ψωμί ολικής άλεσης ή με προζυμένιο ψωμί. Αποφύγετε την συστηματική κατανάλωση τοστ και σάντουιτς ή δημητριακών με γάλα.

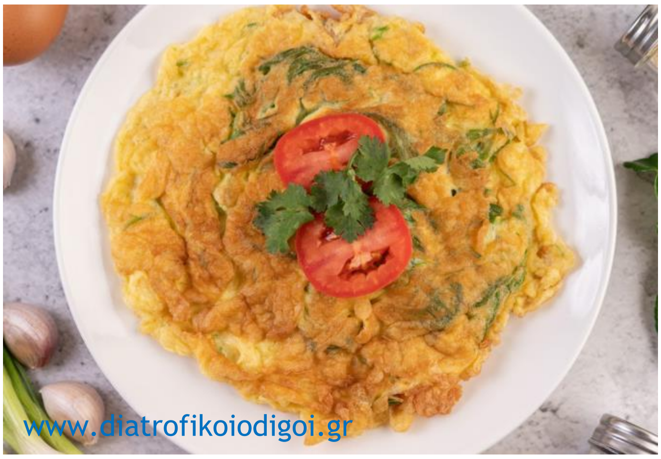

ΤΑ ΕΝΔΙΑΜΕΣΑ ΜΙΚΡΟΓΕΥΜΑΤΑ ή ΣΝΑΚ

Τα μικρογεύματα ή σνακ των παιδιών μας μπορούν να συμβάλουν σημαντικά στη ρύθμιση του βάρους τους καθώς ανάλογα με τις επιλογές μπορούν να προσφέρουν κορεσμό, ενέργεια και πολύτιμα θρεπτικά συστατικά (καλά λιπαρά, πρωτεΐνες, βιταμίνες, μέταλλα, φυτικές ίνες) για την ανάπτυξη των παιδιών ή άδειες θερμίδες. Προτιμάτε σπιτικά γλυκά για τα παιδιά όπως κέικ με ελαιόλαδο και αλεύρι ολικής άλεσης, προσθέτοντας τη μισή από την προτεινόμενη ζάχαρη. Προτιμήστε ελληνικά παραδοσιακά γλυκίσματα που περιέχουν ελαιόλαδο, ξηρούς καρπούς, φρούτα ή μέλι (π.χ. σιμιγδαλένιο χαλβά, καρυδόπιτα, παστέλι, μηλόπιτα). Το μέλι περιέχει γλυκόζη και φρουκτόζη, όμως σε αντίθεση με τη ζάχαρη περιέχει και άλλα ευεργετικά για την υγεία συστατικά με αντιοξειδωτι- κή και αντιμικροβιακή δράση, ενώ η μαύρη σοκολάτα με υψηλή περιεκτι- κότητα σε κακάο, περιέχει συστατικά με ευεργετική επίδραση στην υγεία, όπως τα φλαβονοειδή, και για αυτό το λόγο αποτελεί μια καλή επιλογή γλυκίσματος, όταν καταναλώνεται με μέτρο. Για αλμυρό σνακ, προτιμήστε σπιτικά ποπ κορν με ελαιόλαδο και λίγο αλάτι.

Αποφεύγετε ιδιαίτερα την κατανάλωση αναψυκτικών, ενεργειακών ποτών ή χυμών που περιέχουν προστιθέμενα σάκχαρα. Ωστόσο δεν απαγορεύουμε κανένα τρόφιμο από τη διατροφή των παιδιών, αλλά τα καταναλώνουμε όλα υπό συνθήκες, όπως φαίνεται παραστατικά στο παρακάτω σχήμα:

Στην εκπαίδευση για τη συχνότητα και τους τύπους των γευμάτων, συχνά μιλάμε για τον καταμερισμό των ευθυνών στα γεύματα και τα σνακ του παιδιού, δηλαδή ότι το «τι» και το «πότε» είναι ευθύνη του γονέα ή του φροντιστή, και το «εάν» και το «πόσο» είναι ευθύνη του παιδιού, ξεκινώντας από τις μικρές ηλικίες ως την εφηβεία. Όμως τι ακριβώς σημαίνει αυτό; Το «τι» αναφέρεται στην ποιότητα της τροφής και στην πρόταση της μερίδας πάνω στο τραπέζι, το «πότε» στην ευθύνη των γονέων να προσφέρουν γεύματα ή σνακ σε περίπου σταθερές ώρες, ενώ το «εάν» και το «πόσο» στην δυνατότητα αυτορρύθμισης του παιδιού ή του εφήβου στην ποσότητα του φαγητού που έχει ανάγκη να καταναλώσει προκειμένου να ικανοποιήσει το αίσθημα της πείνας. Ο γονέας χρειάζεται να μπορεί να επιτρέψει στο παιδί του να απορρίψει κάποια τροφή ή να καταναλώσει την ποσότητα της τροφής που έχει ανάγκη. Είναι όμως πολύ σημαντικό, να επιτραπεί στο παιδί να καταναλώσει ελεύθερα, να έχει προσφερθεί από το γονέα η σωστή ποιότητα τροφής με την μερίδα που αναλογεί στο παιδί του. Πώς μπορούν οι γονείς να γνωρίζουν πόσο και τι φαγητό να προσφέρουν στα παιδιά; Ας δούμε τις μερίδες.

Τα σνακ μου

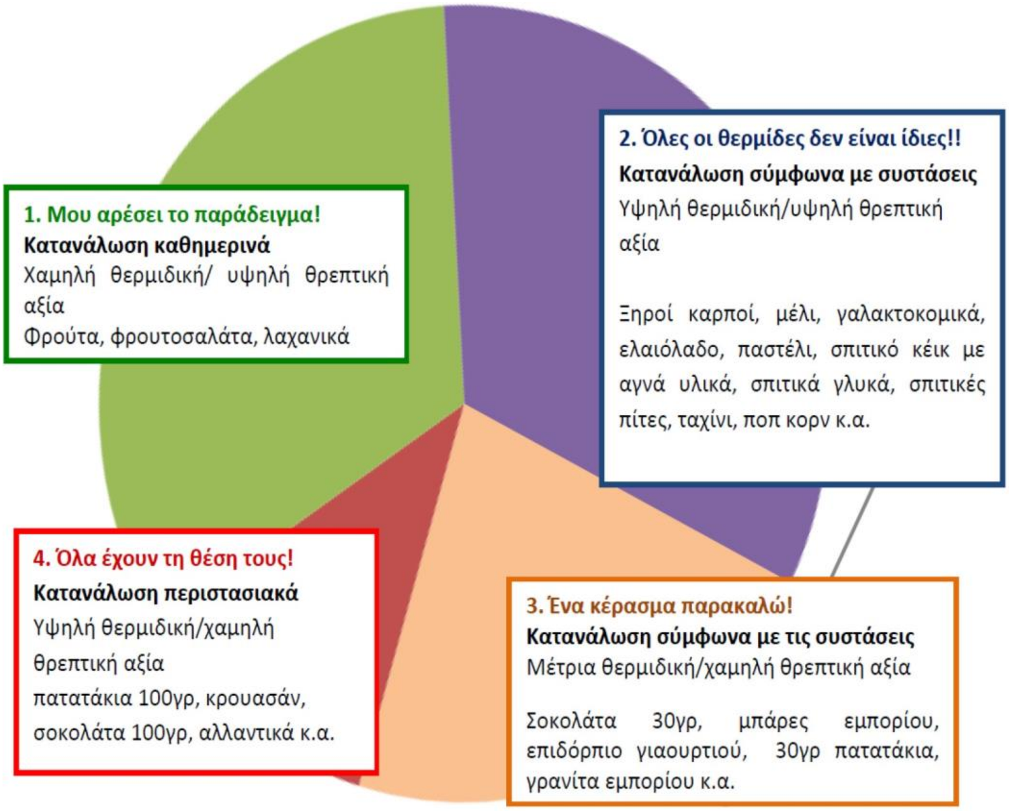

«Πόσο;» Ενδεικτικές μερίδες ανάλογα με την ηλικία για παιδιά με καθιστική ζωή (με βάση τις μερίδες που συστήνονται από τον Εθνικό Διατροφικό Οδηγό Βρεφών, Παιδιών και Εφήβων)

Φρούτα και Λαχανικά

Τα φρούτα και τα λαχανικά είναι πλούσια σε βιταμίνες και θρεπτικά συστατικά όπως βιταμίνη C, βιταμίνες του συμπλέγματος B, βιταμίνη K, φλαβονοειδή, καροτενοειδή, φυλλικό οξύ, ασβέ- στιο, κάλιο, φυτικές ίνες και άλλα συστατικά, πολύτιμοι σύμμαχοι για την υγεία την δική σας και του παιδιού σας.

Είναι σημαντικό να καταναλώνει το παιδί σας ποικιλία φρούτων και λαχανικών καθημερινά!

Προσπαθήστε να συμπεριλάβετε λαχανικά σε κάθε κύριο γεύμα του. Η κατανάλωση λαχανικών σε ποσότητα μεγαλύτερη από την προτεινόμενη είναι επιθυμητή.

Ενδεικτικές μερίδες λαχανικών ανάλογα με την ηλικία
(σελ. 48 του Εθνικού Διατροφικού Οδηγού για Βρέφη, Παιδιά και Εφήβους)

1-2 ετών	2-3 ετών	4-8 ετών	9-13 ετών	14-18 ετών
1 μερίδα / ημέρα	1 μερίδα / ημέρα	1-2 μερίδες / ημέρα	2-3 μερίδες / ημέρα	3-4 μερίδες / ημέρα

Σημείωση: 1 μερίδα ισοδυναμεί με περίπου 150-200 γραμμάρια ωμά ή μαγειρεμένα λαχανικά.

ΠΑΡΑΔΕΙΓΜΑΤΑ ΜΕΡΙΔΑΣ

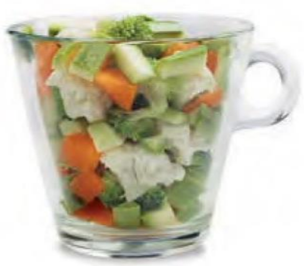

1 φλιτζάνι των 240 ml μαγειρεμένα ή ψιλοκομμένα ωμά λαχανικά

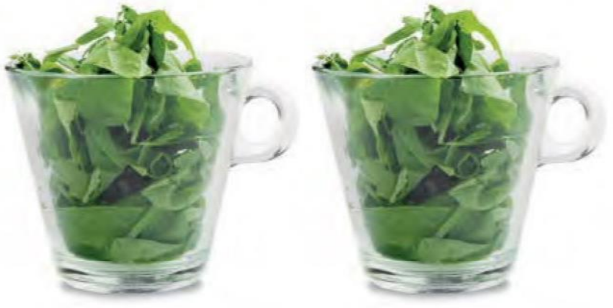

2 φλιτζάνια των 240 ml ωμά πράσινα φυλλώδη λαχανικά, π.χ., μαρούλι, ρόκα κ.ά.

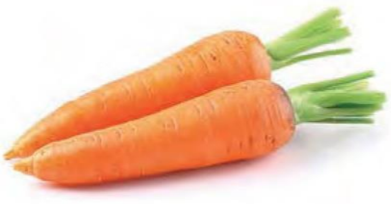

2 μέτρια καρότα

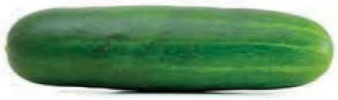

1 μέτριο αγγούρι

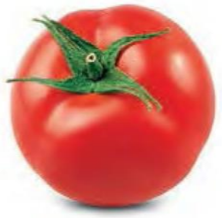

1 μεγάλη ντομάτα ή 1 φλιτζάνι των 240 ml τριμμένη ντομάτα

www.diatrofikoiodigoi.gr

(σελ. 48 του Εθνικού Διατροφικού Οδηγού για Βρέφη, Παιδιά και Εφήβους)

Δώστε φρούτο στο παιδί σας ενδιαμέσως των γευμάτων ή για επιδόρπιο εάν αυτό είναι αποδεκτό ή όταν το παιδί σας δυσκολεύεται να φάει λαχανικά με το γεύμα του.

Ενδεικτικές μερίδες φρούτων ανάλογα με την ηλικία
(σελ. 51 του Εθνικού Διατροφικού Οδηγού για Βρέφη, Παιδιά και Εφήβους)

1-2 ετών	2-3 ετών	4-8 ετών	9-13 ετών	14-18 ετών
1 μερίδα / ημέρα	1 μερίδα / ημέρα	1-2 μερίδες / ημέρα	2-3 μερίδες / ημέρα	3 μερίδες / ημέρα

Σημείωση: 1 μερίδα ισοδυναμεί με περίπου 120-200 γραμμάρια φρούτου.

ΠΑΡΑΔΕΙΓΜΑΤΑ ΜΕΡΙΔΑΣ

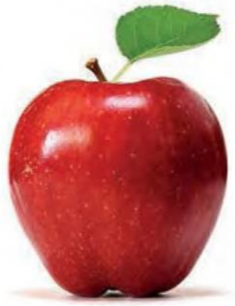

1 μέτριου μεγέθους φρούτο, π.χ., μήλο, πορτοκάλι, ροδάκινο, αχλάδι, μπανάνα

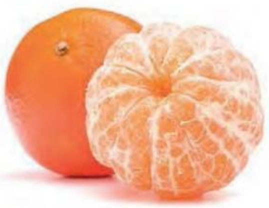

2 μικρά φρούτα, π.χ., μανταρίνια, βανίλιες, ακτινίδια

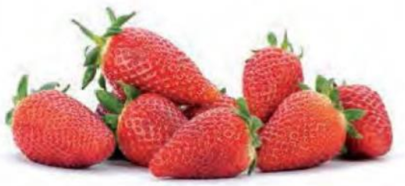

Περίπου 8 φράουλες

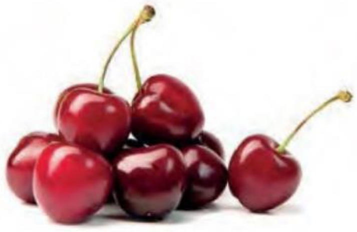

Περίπου 15 κεράσια

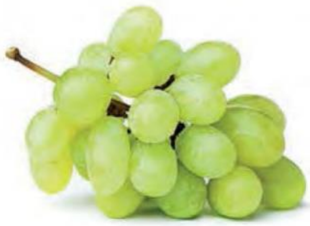

Περίπου 30 ρώγες σταφυλιού (μεγέθους σουλτανίνας)

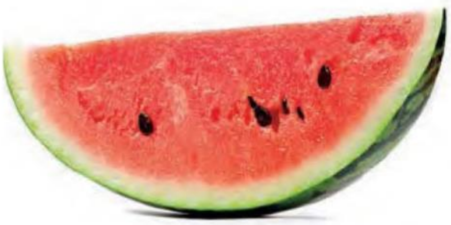

1 φέτα καρπούζι ή πεπόνι

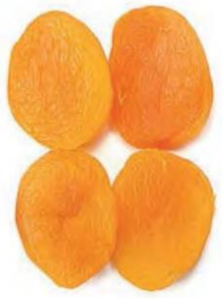

4 αποξηραμένα φρούτα, π.χ., βερίκοκα, δαμάσκηνα

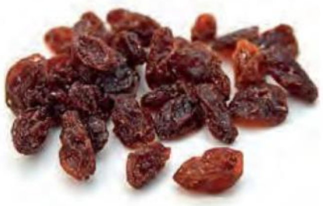

1½ κουταλιά της σούπας σταφίδες

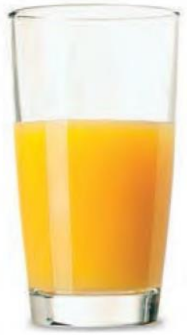

½ ποτήρι φυσικό χυμό (125 ml)

www.diatrofikoiodigoi.gr

(σελ. 52 του Εθνικού Διατροφικού Οδηγού για Βρέφη, Παιδιά και Εφήβους)

Γάλα και γαλακτοκομικά προϊόντα

Το γάλα και τα προϊόντα του παρέχουν πρωτεΐνες υψηλής βιολογικής αξίας, αποτελούν μία από τις πλουσιότερες πηγές ασβεστίου για τον οργανισμό και περιέχουν βιταμίνες όπως B2, B12, A και D και ανόργανα στοιχεία όπως κάλιο, φώσφορο, μαγνήσιο, ψευδάργυρο. Φροντίστε ώστε το παιδί να καταναλώνει γάλα ή / και γαλακτοκομικά προϊόντα στις συνιστώμενες μερίδες, ενώ θυμηθείτε πως δεν χρειάζονται υπερβολές.

Ενδεικτικά, ανάλογα με την ηλικία, η κατανάλωση γάλακτος και γαλακτοκομικών προϊόντων μπορεί να είναι:

(σελ. 58 του Εθνικού Διατροφικού Οδηγού για Βρέφη, Παιδιά και Εφήβους)

1-2 ετών	2-3 ετών	4-8 ετών	9-13 ετών	14-18 ετών
2 μερίδες / ημέρα	2 μερίδες / ημέρα	2-3 μερίδες / ημέρα	3-4 μερίδες / ημέρα	3-4 μερίδες / ημέρα

1 ΜΕΡΙΔΑ ΙΣΟΔΥΝΑΜΕΙ ΜΕ:

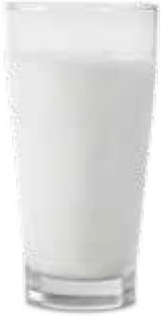

1 ποτήρι γάλα (250 ml)

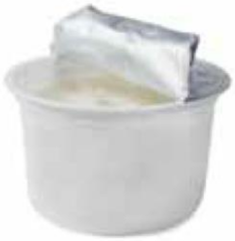

1 κεσεδάκι γιαούρτι (200 γραμμάρια)

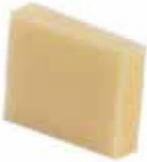

1 κομμάτι σκληρό τυρί μεγέθους σπιρτόκουτου (30 γραμμάρια, π.χ., φέτα, γραβιέρα)

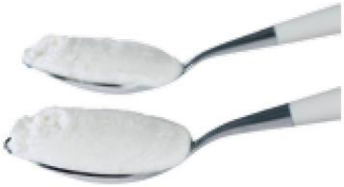

2 κουταλιές της σούπας μαλακό τυρί (60 γραμμάρια, π.χ., ανθότυρο, κατίκι, μυζήθρα)

1 φέτα τυρί για τοστ (30 γραμμάρια, π.χ., κασέρι)

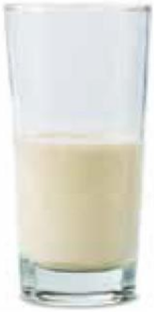

½ ποτήρι γάλα (125 ml) συμπυκνωμένο (εβαπορέ)

www.diatrofikoiodigoi.gr

(σελ. 58 του Εθνικού Διατροφικού Οδηγού για Βρέφη, Παιδιά και Εφήβους)

Δημητριακά και Πατάτες

Τα δημητριακά και οι πατάτες είναι βασική πηγή υδατανθράκων, προσφέρουν ενέργεια στον οργανισμό και πρέπει να καταναλώνονται από τα παιδιά καθημερινά. Τα δημητριακά ολικής άλεσης περιέχουν φυτικές ίνες, βιταμίνες του συμπλέγματος Β, ανόργανα στοιχεία, ενώ η πατάτα βιταμίνη C, βιταμίνη Β6, κάλιο.

Αποφύγετε στην καθημερινότητά σας τις επεξεργασμένες πηγές υδατανθράκων (μπισκότα εμπορίου, κρουασάν κ.ά.) και προτιμήστε λιγότερο επεξεργασμένα προϊόντα (ψωμί ολικής αλέσεως από το φούρνο, ψωμί με προζύμι, παξιμάδια ολικής ή κρίθινα κ.ά.).

Ενδεικτικά, ανάλογα με την ηλικία, η κατανάλωση δημητριακών και πατάτας μπορεί να είναι:

(σελ. 65 του Εθνικού Διατροφικού Οδηγού για Βρέφη, Παιδιά και Εφήβους)

1-2 ετών	2-3 ετών	4-8 ετών	9-13 ετών	14-18 ετών
2 μερίδες / ημέρα	3 μερίδες / ημέρα	4-5 μερίδες / ημέρα	5-6 μερίδες / ημέρα	6-8 μερίδες / ημέρα

1 ΜΕΡΙΔΑ ΙΣΟΔΥΝΑΜΕΙ ΜΕ:

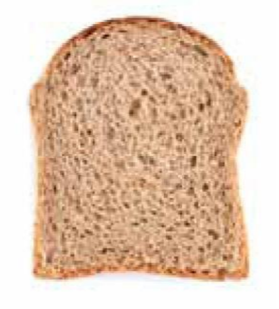

1 φέτα ψωμί (30 γραμμάρια)

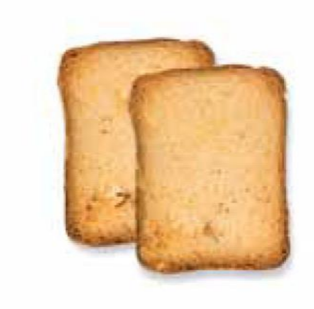

2 φρυγανιές ή 1 παξιμάδι (μεσαίου μεγέθους)

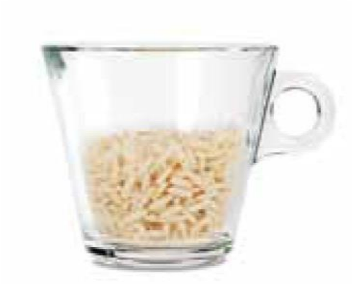

½ φλιτζάνι των 240 ml μαγειρεμένα ζυμαρικά ή ρύζι ή πλιγούρι (70-90 γραμμάρια)

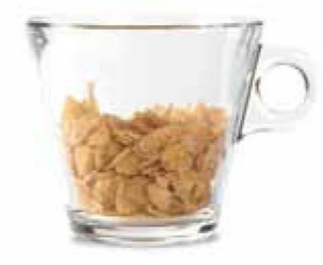

½ φλιτζάνι των 240 ml δημητριακά πρωινού (30 γραμμάρια)

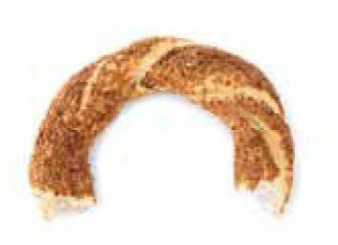

½ κουλούρι Θεσσαλονίκης

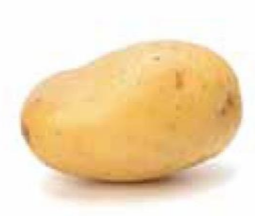

1 πατάτα μέτριου μεγέθους (120-150 γραμμάρια μαγειρεμένη)

www.diatrofikoiodigoi.gr

(σελ. 65 του Εθνικού Διατροφικού Οδηγού για Βρέφη, Παιδιά και Εφήβους)

Όσπρια

Τα όσπρια αξίζουν ξεχωριστή αναφορά διότι είναι τρόφιμα υψηλής θρεπτικής αξίας, πλούσια σε πλήθος θρεπτικών συστατικών και πρωτεΐνης. Φροντίστε ώστε το παιδί να καταναλώνει όσπρια τουλάχιστον μία φορά την εβδομάδα.

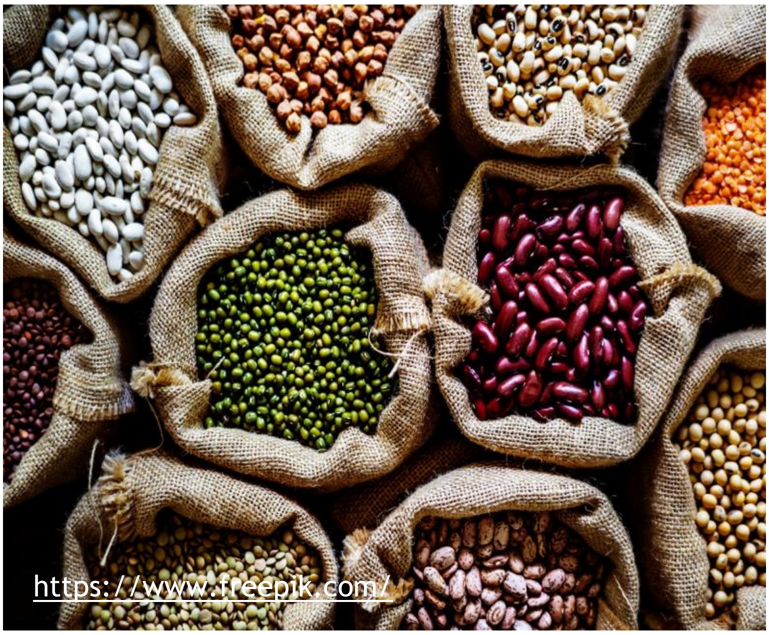

Ενδεικτικά, ανάλογα με την ηλικία, η κατανάλωση οσπρίων μπορεί να είναι:
(σελ. 68 του Εθνικού Διατροφικού Οδηγού για Βρέφη, Παιδιά και Εφήβους)

	1-2 ετών	2-3 ετών	4-8 ετών	9-13 ετών	14-18 ετών
**Αριθμός μερίδων**	1-2 μερίδες / εβδομάδα	έως 3 μερίδες / εβδομάδα	3 μερίδες / εβδομάδα	τουλάχιστον 3 μερίδες / εβδομάδα	τουλάχιστον 3 μερίδες / εβδομάδα
**Ορισμός μερίδας***	40 - 60 γραμμάρια	60 - 90 γραμμάρια	90 - 120 γραμμάρια	120 - 150 γραμμάρια	150 - 200 γραμμάρια

*Η ποσότητα αναφέρεται σε μαγειρεμένα στραγγισμένα όσπρια.

Κόκκινο (μοσχάρι, χοιρινό, αρνί, κατσίκι) και λευκό κρέας (κοτόπουλο, κουνέλι)

Φροντίστε ώστε τα παιδιά άνω των 2 ετών να καταναλώνουν 2-3 φορές την εβδομάδα κόκκινο ή / και λευκό άπαχο κρέας.

Αποφεύγετε το επεξεργασμένο κρέας: αλλαντικά (από χοιρινό, μοσχάρι, γαλοπούλα, κοτόπουλο κ.ά.) και προπαρασκευασμένα προϊόντα κρέατος (μπιφτέκια, παναρισμένα κομμάτια κοτόπουλου κ.ά.).

Ενδεικτικά, ανάλογα με την ηλικία, η κατανάλωση κρέατος μπορεί να είναι:
(σελ. 71 του Εθνικού Διατροφικού Οδηγού για Βρέφη, Παιδιά και Εφήβους)

	1-2 ετών	2-3 ετών	4-8 ετών	9-13 ετών	14-18 ετών
**Αριθμός μερίδων**	3-4 μερίδες / εβδομάδα	2-3 μερίδες / εβδομάδα	2-3 μερίδες / εβδομάδα	2-3μερίδες / εβδομάδα	2-3μερίδες / εβδομάδα
**Ορισμός μερίδας***	40 - 60 γραμμάρια	60 γραμμάρια	60 - 90 γραμμάρια	90 - 120 γραμμάρια	120 - 150 γραμμάρια

*Η ποσότητα αναφέρεται στο βάρος του μαγειρεμένου κρέατος χωρίς κόκαλα.

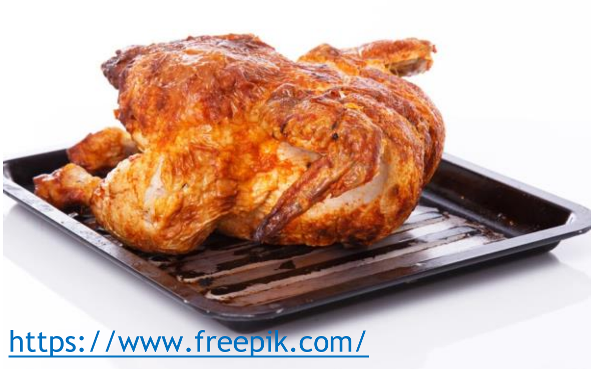

Αυγά

Τα αυγά αποτελούν σημαντική πηγή πρωτεΐνης υψηλής βιολογικής αξίας και περιέχουν πολ- λές βιταμίνες, καροτενοειδή, σελήνιο και χολίνη. Ανήκουν στα βασικά τρόφιμα της διατροφής των παιδιών.

Φροντίστε τα παιδιά να καταναλώνουν 4 - 7 αυγά την εβδομάδα! Για τα παιδιά με υπερλιπιδαιμία συμβουλευτείτε τον γιατρό σας σχετικά με την κατανάλωση αυγών.

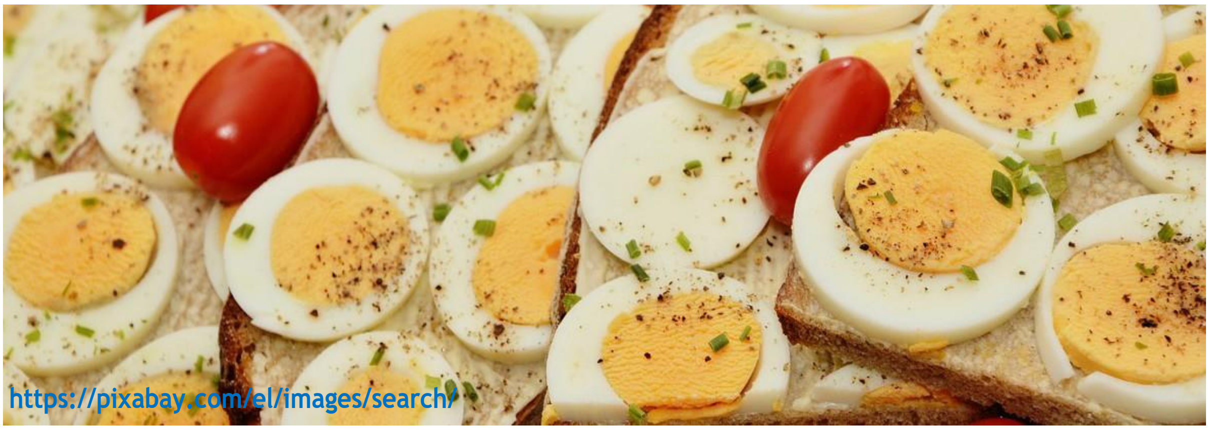

Ψάρια και Θαλασσινά

Φροντίστε ώστε τα παιδιά να καταναλώνουν 2-3 μερίδες από ποικιλία ψαριών και θαλασσινών την εβδομάδα. Τουλάχιστον η 1 μερίδα την εβδομάδα να είναι λιπαρό ψάρι (π.χ., σαρδέλα, γαύρος, σαφρίδι, κολιός, ζαργάνα), τα οποία έχουν υψηλή περιεκτικότητα σε ω-3 λιπαρά οξέα (βλέπε πίνακα).

Ενδεικτικά, ανάλογα με την ηλικία, η κατανάλωση ψαριών μπορεί να είναι:

(σελ. 75 του Εθνικού Διατροφικού Οδηγού για Βρέφη, Παιδιά και Εφήβους)

	1-2 ετών	2-3 ετών	4-8 ετών	9-13 ετών	14-18 ετών
**Αριθμός μερίδων**	2 μερίδες / εβδομάδα	2 μερίδες / εβδομάδα	2-3 μερίδες / εβδομάδα	2-3μερίδες / εβδομάδα	2-3μερίδες / εβδομάδα
**Ορισμός μερίδας***	60 γραμμάρια	60 - 90 γραμμάρια	90 - 120 γραμμάρια	120 - 150 γραμμάρια	150 γραμμάρια

*Η ποσότητα αναφέρεται στο βάρος του μαγειρεμένου ψαριού χωρίς κόκαλα.

Κατάταξη ψαριών & θαλασσινών, ανάλογα με την περιεκτικότητά τους σε ω-3 λιπαρά οξέα

(σελ. 77 του Εθνικού Διατροφικού Οδηγού για Βρέφη, Παιδιά και Εφήβους)

ΨΑΡΙΑ – ΘΑΛΑΣΣΙΝΑ	ω-3 ΛΙΠΑΡΑ (ΓΡΑΜΜΑΡΙΑ) / 100 ΓΡΑΜΜΑΡΙΑ
Σαρδέλα	1,41
Καπόνι	1,35
Κολιός – Σκουμπρί	1,34
Σολομός (φρέσκος)	1,03
Μελανούρι	0,96
Γόπα	0,86
Γαλέος	0,84
Γαύρος	0,8
Ξιφίας	0,75
Πέστροφα	0,73
Γοβιός	0,68
Μαρίδα – Αθερίνα	0,61
Λαβράκι	0,59
Καλαμάρι	0,48
Τσιπούρα – Σπάρος – Σαργός	0,48
Σολομός καπνιστός	0,45
Κέφαλος	0,32
Καβούρι	0,32
Σαφρίδι	0,26
Παλαμίδα	0,26
Ροφός	0,25
Γλώσσα	0,25
Τόνος (κονσέρβας)	0,24
Γαρίδα	0,24
Λυθρίνι	0,22
Βακαλάος φρέσκος	0,18
Σάλπα	0,17
Χταπόδι	0,16
Καραβίδα	0,14
Χέλι	0,12
Αστακός	0,11
Μύδια	0,11
Τόνος	0,10

Πηγές: Zlatanos S. & Sagredos AN. (1993). The fatty acid composition of some important Mediterranean fish species. *Fett/Lipid*, 95 (2), 66-69.
Στεργίου ΙΚ., Καραχλέ ΠΚ., Τσικλήρας Α. & Μαμαλάκης Η. (2011). *Κραυγή ιχθύος – Ψάρια των ελληνικών θαλασσών – Βιολογία, Αλιεία, Διαχείριση*. Αθήνα: Εκδόσεις Πατάκη.
USDA (2013). USDA National Nutrient Database for Standard Reference.

Λίπη, έλαια, ελιές και ξηροί καρποί

Τα λίπη και τα έλαια είναι πολύ σημαντικά για την υγεία του οργανισμού μας. Αποτελούν συστατικά των μεμβρανών των κυττάρων και των ορμονών, συμβάλλουν στην απορρόφηση λιποδιαλυτών βιταμινών και αποτελούν τη μεγαλύτερη αποθήκη ενέργειας του οργανισμού (λιπώδης ιστός). Τα λιπίδια είτε περιέχονται στα τρόφιμα ως φυσικό συστατικό τους (π.χ. κρέας, γαλακτοκομικά προϊόντα, αυγό) είτε προστίθενται σε αυτά (π.χ. ελαιόλαδο κατά το μαγείρεμα). Σημασία για την υγεία μας έχει τόσο η ποσότητα όσο και η ποιότητα των λιπιδίων που καταναλώνουμε (π.χ. το ελαιόλαδο, οι ξηροί καρποί και το λίπος των ψαριών έχουν ευεργετικές επιδράσεις στην υγεία, βλέπε πίνακα με κατηγορίες λιπαρών οξέων).

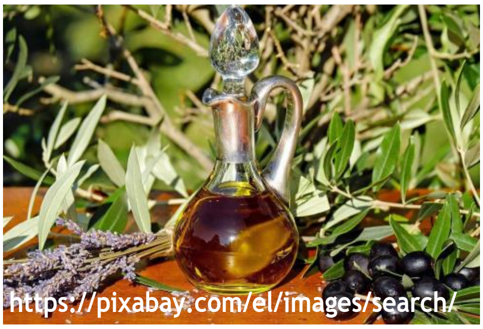

Προτιμήστε το ελαιόλαδο ως πρώτη επιλογή προστιθέμενου ελαίου στη διατροφή του παιδιού, τόσο στο μαγείρεμα όσο και στη σαλάτα.

Περιορίστε την κατανάλωση προστιθέμενων λιπών ζωικής προέλευσης, όπως το βούτυρο, και αντικαταστήστε τα με ελαιόλαδο, όπου είναι εφικτό.

Αποφεύγετε την κατανάλωση υδρογονωμένων λιπαρών (trans λιπαρά), τα οποία μπορεί να περιέχονται κυρίως σε βιομηχανοποιημένα προϊόντα, προϊόντα ζαχαροπλαστικής ή προϊόντα ταχυφαγείου. Ελέγξτε τις ετικέτες στις συσκευασίες των τροφίμων πριν τα αγοράσετε!

Ενδεικτικά ανάλογα με την ηλικία, η κατανάλωση λιπών και ελαίων μπορεί να είναι:
(σελ. 80 του Εθνικού Διατροφικού Οδηγού για Βρέφη, Παιδιά και Εφήβους)

1-2 ετών	2-3 ετών	4-8 ετών	9-13 ετών	14-18 ετών
1 μερίδα / ημέρα	1-2 μερίδες / ημέρα	2-3 μερίδες / ημέρα	3-4 μερίδες / ημέρα	4-5 μερίδες / ημέρα

1 ΜΕΡΙΔΑ ΙΣΟΔΥΝΑΜΕΙ ΜΕ:

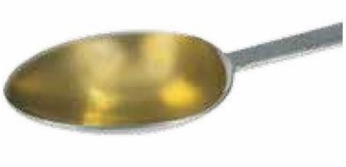

1 κουταλιά της σούπας (των 15 ml) ελαιόλαδο

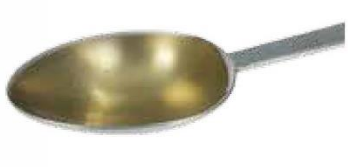

1 κουταλιά της σούπας (των 15 ml) άλλα φυτικά έλαια

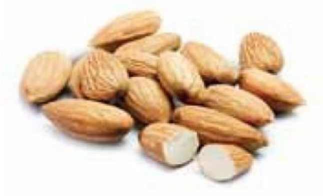

1 χούφτα ξηρών καρπών (π.χ., 18 αμύγδαλα, 6 ολόκληρα καρύδια, 3 κουταλιές της σούπας ηλιόσπορους)

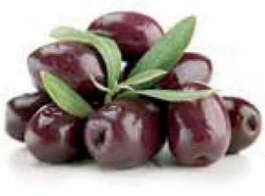

10-12 ελιές

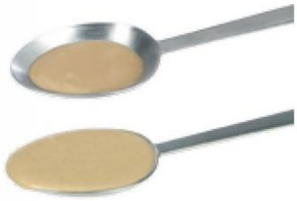

1½ κουταλιά της σούπας (των 15ml) ταχίνι (25 γραμμάρια)

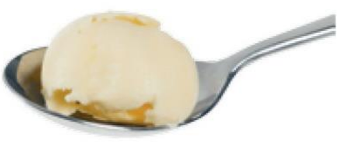

1 κουταλιά της σούπας (των 15 ml) βούτυρο ή μαργαρίνη

www.diatrofikoiodigoi.gr

(σελ. 81 του Εθνικού Διατροφικού Οδηγού για Βρέφη, Παιδιά και Εφήβους)

ΚΑΤΗΓΟΡΙΕΣ ΛΙΠΑΡΩΝ ΟΞΕΩΝ, ΠΗΓΕΣ ΠΡΟΣΛΗΨΗΣ ΚΑΙ Η ΣΧΕΣΗ ΤΟΥΣ ΜΕ ΤΗΝ ΥΓΕΙΑ

(σελ. 82 του Εθνικού Διατροφικού Οδηγού για Βρέφη, Παιδιά και Εφήβους)

ΑΚΟΡΕΣΤΑ ΛΙΠΑΡΑ (ΜΟΝΟΑΚΟΡΕΣΤΑ & ΠΟΛΥΑΚΟΡΕΣΤΑ)	ΚΟΡΕΣΜΕΝΑ ΛΙΠΑΡΑ	ΥΔΡΟΓΟΝΩΜΕΝΑ ΛΙΠΑΡΑ (ΤΡΑΝΣ)
**Πηγές**<br>Ελαιόλαδο, ελιές, ξηροί καρποί, ψάρια & θαλασσινά κ.ά.	**Πηγές**<br>Κρέας, αλλαντικά, βούτυρο, γαλακτοκομικά κ.ά.	**Πιθανές πηγές***<br>Μαργαρίνες, μπισκότα, κέικ, γλυκά, προϊόντα σφολιάτας, άλλα βιομηχανοποιημένα προϊόντα, προϊόντα ταχυφαγείου κ.ά.<br>*Ελέγξτε τις ετικέτες της συσκευασίας των τροφίμων.
**Ωφέλιμα για την υγεία**<br>✓ Κατανάλωση σύμφωνα με τις συστάσεις	**Επιβλαβή για την υγεία όταν καταναλώνονται σε ποσότητες μεγαλύτερες των συστάσεων**<br>✓ Μείωση κατανάλωσης<br>✓ Αντικατάσταση από ακόρεστα λιπαρά (μονο-ακόρεστα & πολυακόρεστα)	**Πολύ επιβλαβή για την υγεία**<br>✓ Αποφυγή κατανάλωσης

www.diatrofikoiodigoi.gr

Νερό και αφεψήματα

Φροντίστε ώστε τα παιδιά να πίνουν αρκετό νερό κατά τη διάρκεια της ημέρας. Επίσης φροντίστε τα παιδιά να πίνουν νερό αντί για χυμούς ή αναψυκτικά. Το νερό συμμετέχει σε όλες σχεδόν τις λειτουργίες του οργανισμού μας. Η ανεπαρκής πρόσληψη υγρών μπορεί να οδηγήσει σε αφυδάτωση με αρνητικές συνέπειες τόσο στη φυσική αντοχή αλλά και στη νοητική λειτουργία των παιδιών και των εφήβων.

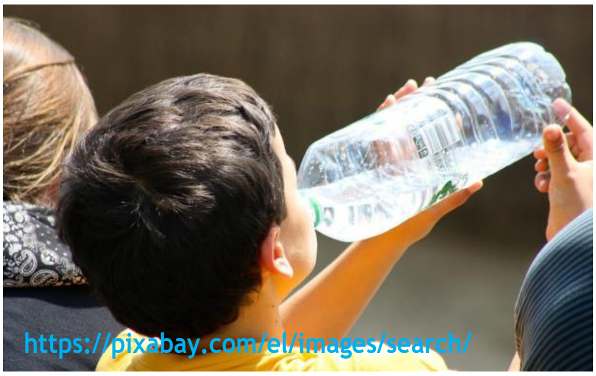

Ενδεικτικά, ανάλογα με την ηλικία, η κατανάλωση υγρών μπορεί να είναι:

(σελ. 91 του Εθνικού Διατροφικού Οδηγού για Βρέφη, Παιδιά και Εφήβους)

	1-2 ετών	2-3 ετών	4-8 ετών	9-13 ετών	14-18 ετών
**Υγρά* (νερό, γάλα, ροφήματα, χυμοί, σούπες κ.ά.)**	5 ποτήρια / ημέρα	5 ποτήρια / ημέρα	6-7 ποτήρια / ημέρα	8-10 ποτήρια / ημέρα	10-12 ποτήρια / ημέρα
**Νερό**	3-4 ποτήρια / ημέρα	3-4 ποτήρια / ημέρα	4-5 ποτήρια / ημέρα	6-8 ποτήρια / ημέρα	8-10 ποτήρια / ημέρα

www.diatrofikoiodigoi.gr

*Θα πρέπει να δίνεται έμφαση στην κατανάλωση νερού. Το νερό συμπεριλαμβάνεται στην πρόσληψη υγρών.

Να θυμάστε ότι αν το παιδί ή ο έφηβος κάνει έντονη σωματική δραστηριότητα χρειάζεται να καταναλώνει περισσότερο νερό.

«Τι;» Ποιότητα, προετοιμασία και μαγείρεμα τροφίμων

Ποιότητα τροφών και ψώνια

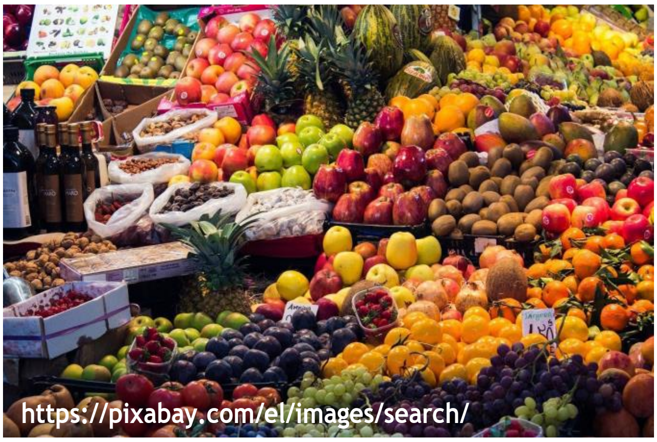

Η ελληνική παραδοσιακή διατροφή χαρακτηρίζεται από πολλές υγιεινές επιλογές τροφών, οι περισσότερες των οποίων είναι οικονομικές (π.χ. φρούτα, λαχανικά, όσπρια, δημητριακά, αυγά, μικρά ψάρια της Μεσογείου όπως σαρδέλες, γαύρος, μαρίδα). Ο σωστός προγραμματισμός, απαιτώντας λίγο χρόνο και σκέψη, βοηθά στο να τρεφόμαστε σωστά και οικονομικά (π.χ. λίστα για ψώνια, έρευνα αγοράς, προγραμματισμός γευμάτων της εβδομάδας). Προτιμήστε τα εποχιακά λαχανικά τα οποία είναι μια πιο υγιεινή και οικονομική επιλογή, αλλά μη ξεχνάτε πως και τα κατεψυγμένα λαχανικά είναι μια ασφαλής επιλογή. Η χαμηλή θερμοκρασία διατηρεί τα θρεπτικά τους συστατικά εφόσον τα λαχανικά έχουν καταψυχθεί αμέσως μετά την συγκομιδή!

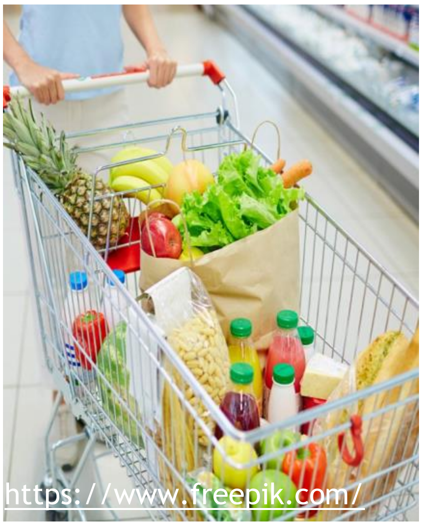

Αποφεύγετε τη συχνή αγορά επεξεργασμένων τροφίμων (π.χ. μπισκότα, πατατάκια, αλλαντικά), τα οποία είναι συνήθως ακριβότερα και όταν καταναλώνονται συχνά, έχουν αρνητικές επιδράσεις στη υγεία, και αποφεύγετε να κάνετε τις αγορές σας όταν βιάζεστε ή πεινάτε. Είναι πιο εύκολο να παρασυρθείτε και να αγοράσετε προϊόντα που πιθανώς δεν χρειάζεστε!

Τι να προσέξω στις ετικέτες;

Είναι καλό πάντα να ελέγχουμε τις ετικέτες των συσκευασμένων τροφίμων που καταναλώνουν τα παιδιά. Προτιμήστε τα συστατικά που αναγράφονται να είναι όσο το δυνατόν πιο φυσικά. Ποια όμως συστατικά αξίζει να ελέγξουμε;

Αλάτι: Καθώς η υπερβολική κατανάλωση νατρίου ήδη από την παιδική ηλικία, αυξάνει μακροπρόθεσμα τον κίνδυνο εμφάνισης υπέρτασης και καρδιαγγειακών νοσημάτων κατά την ενή- λικη ζωή συνιστάται η συνολική ημερήσια κατανάλωση αλατιού να περιορίζεται στα 2 γραμ- μάρια / ημέρα στην ηλικία των 1 - 3 ετών, στα 3 γραμμάρια / ημέρα στην ηλικία των 4 - 6 ετών και στα 5 γραμμάρια / ημέρα στην ηλικία των 7 - 18 ετών (όπως και στους ενήλικες). Τα 5 γραμμάρια αλατιού ισοδυναμούν με 1 κουταλάκι του γλυκού. Ελέγχετε τις ετικέτες ώστε ένα τρόφιμο να μην περιέχει περισσότερο από 0,6 γρ νατρίου ή 1,5γρ αλατιού ανά 100γρ τροφίμου καθώς τότε χαρακτηρίζεται υψηλό σε Νάτριο.

Ζάχαρη και γλυκαντικές ύλες (γλυκόζη, αμυλοσιρόπιο, φρουκτόζη, μαλτόζη, μαλτοδεξτρίνη, μελάσα, πετιμέζι): Τα προστιθέμενα σάκχαρα, με κύριο εκπρόσωπο τη ζάχαρη, προσφέρουν ενέργεια στον οργανισμό, χωρίς όμως να του παρέχουν άλλα θρεπτικά συστατικά, προσδίδοντας κατά βάση «κενές θερμίδες». Ελέγχετε τις ετικέτες ώστε ένα τρόφιμο να μην περιέχει περισσότερο από 22,5 γρ ζάχαρης ανά 100γρ τροφίμου καθώς τότε χαρακτηρίζεται υψηλό σε ζάχαρη.

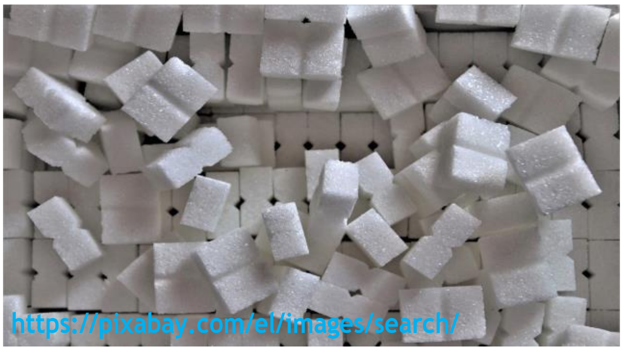

Η κατανάλωση γλυκαντικών υλών που δεν αποδίδουν θερμίδες, τεχνητές (π.χ. ασπαρτάμη, σουκραλόζη κ.α.) ή φυσικές (π.χ. στέβια) δεν έχει βρεθεί ότι σχετίζεται με προβλήματα υγείας, **ΟΜΩΣ** ιδιαίτερα στην περίπτωση των παιδιών και των εφήβων, η χρήση τους πρέπει να γίνεται με μέτρο και να αποφεύγεται η υπερκατανάλωσή τους, κυρίως διότι περιέχουν φωσφορικά άλατα, τα οποία μειώνουν την απορρόφηση του ασβεστίου, όπως ακριβώς και τα αναψυκτικά με ζάχαρη.

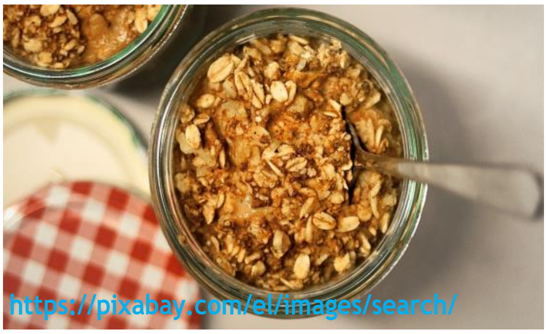

Φυτικές Ίνες: Ελέγχετε τις ετικέτες ώστε ένα τρόφιμο να περιέχει κατά προτίμηση περισσότερο από 6γρ φυτικές ίνες στα 100γρ προϊόντος καθώς τότε χαρακτηρίζεται ως υψηλής περιεκτικότητας σε φυτικές ίνες.

Υδρογονωμένα (trans) λιπαρά: Ελέγχετε τις ετικέτες για να μην περιέχουν τα συσκευασμένα τρόφιμα υδρογονωμένα λιπαρά. Ένα τρόφιμο που αναγράφει πως δεν περιέχει υδρογονωμένα λιπαρά, περιέχει λιγότερο από 2 γρ ανά 100γρ λί- πους στο τρόφιμο.

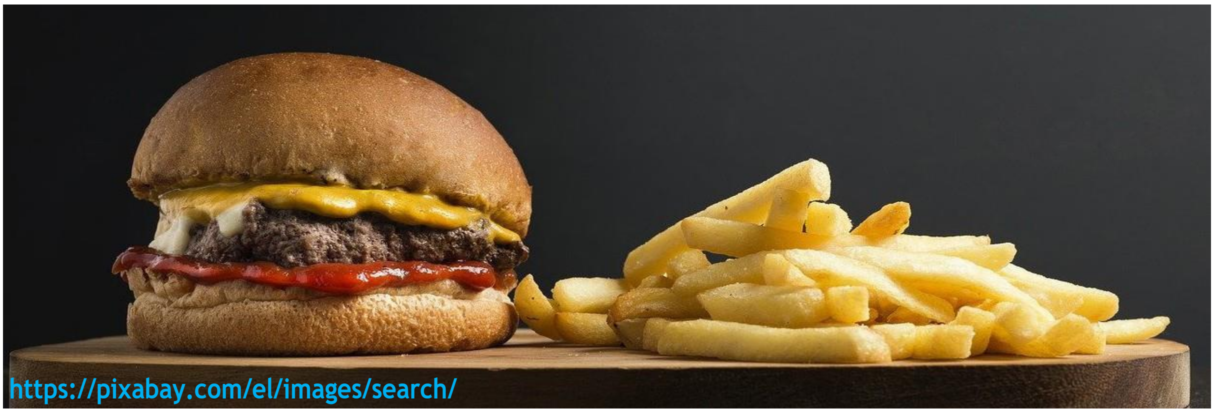

Προετοιμασια Και Μαγειρεμα Τροφιμων

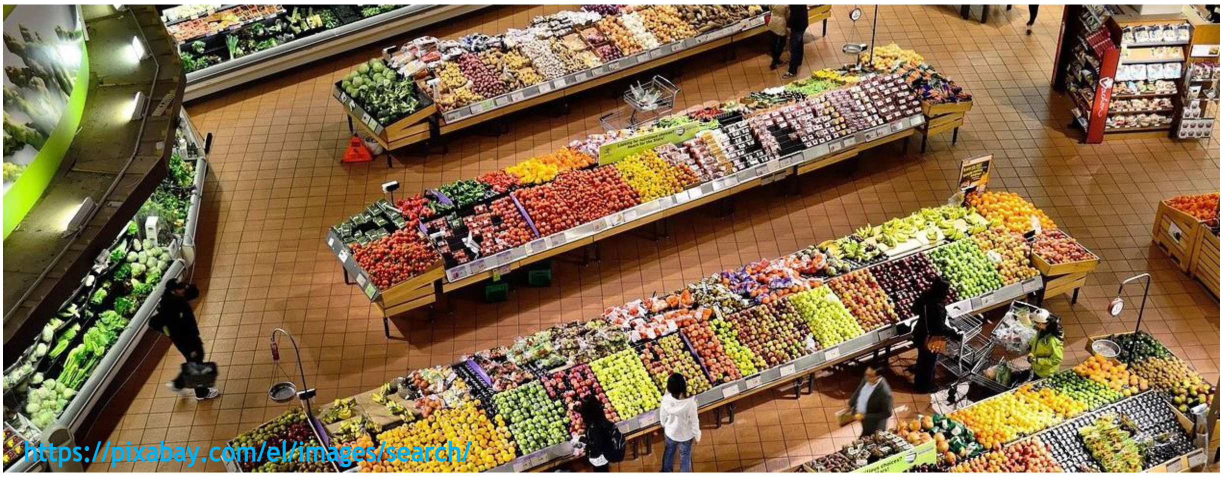

Ο **σωστός προγραμματισμός** και η οργάνωση των σπιτικών γευμάτων και τροφών που εί- ναι διαθέσιμα στο σπίτι, σε συνεργασία με το παιδί, προάγει την αυτονομία του παιδιού σας και βοηθά στην αυτορρύθμιση του βάρους του.

Έχοντας το παραπάνω στο μυαλό σας, φροντίστε στον προγραμματισμό των γευμάτων της εβδομάδας να ρωτήσετε τα παιδιά σας τις προτιμήσεις τους για το μενού, να κάνετε πολλές υγιεινές προτάσεις γευμάτων και να τα αφήσετε να διαλέξουν ποιά προτιμούν. Συνδυάστε τα φαγητά με τέτοιο τρόπο που να ενισχύετε την κατανάλωση των λιγότερο εύληπτων φαγητών, καθώς έχει φανεί πως ο συνδυασμός ενός αγαπητού τροφίμου (πχ η αγαπημένη σως) με ενός λιγότερου αγαπητού (λαχανικά), ενθαρρύνει την κατανάλωση!

Φροντίστε στον προγραμματισμό σας να συμπεριλάβετε επιλογές για θρεπτικά σπιτικά σνακ. Δώστε υγιεινές επιλογές και αφήστε τα παιδιά σας να βοηθήσουν στην προετοιμασία.

Μην ξεχνάτε να διαπραγματευτείτε με τα παιδιά σας τους όρους κατανάλωσης των επεξεργασμένων τροφών, ώστε να εντάξετε τρόφιμα που τυχόν επιθυμούν στο εβδομαδιαίο τους πρόγραμμα, στις συνιστώμενες μερίδες (π.χ. τα αγαπημένο παγωτό πριν το παιχνίδι ή στην εκδρομή). Κάντε μαζί με τα παιδιά σας τη λίστα για τα ψώνια. Πηγαίνετε μαζί στην αγορά και αφήστε τα να επιλέξουν λαχανικά. Ενθαρρύνετε τα παιδιά να συμμετέχουν στο μαγείρεμα ή επιλέξτε εσείς το χρόνο (π.χ. μια φορά τη βδομάδα) που θα φτιάχνετε μαζί ένα γεύμα ή ένα σνακ!

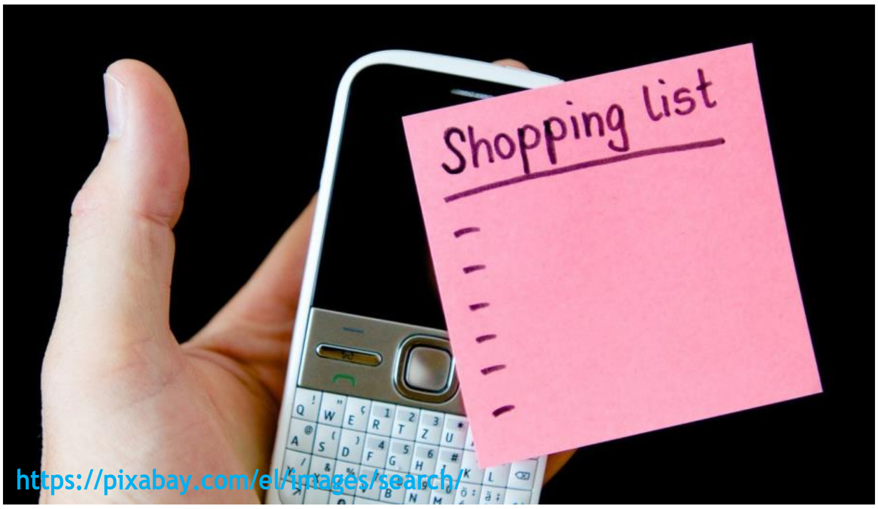

Τροποι Μαγειρεματος

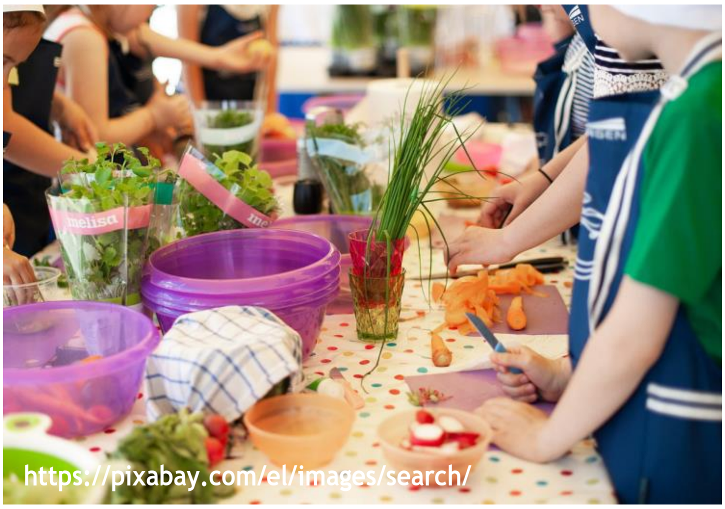

Μαγειρεύουμε με τον ελληνικό παραδοσιακό τρόπο, για όλη την οικογένεια, χρησιμοποιώντας πάντα ελαιόλαδο.

Η ελληνική παραδοσιακή διατροφή έχει αποδεδειγμένες ευεργετικές επιδράσεις στην υγεία οι οποίες οφείλονται στο σύνολο των τροφίμων (στη συνολική τήρηση του προτύπου), στην ποσότητα αλλά και στον τρόπο με τον οποίο καταναλώνονται τα τρόφιμα. Το πρότυπο της ελληνικής παραδοσιακής διατροφής σχετίζεται με πρόληψη χρόνιων νοσημάτων στην ενήλικη ζωή, με αύξηση του προσδόκιμου ζωής, με τη διατήρηση του φυσιολογικού βάρους και με πρόληψη της εμφάνισης της παιδικής παχυσαρκίας. Ακολουθούμε τις παρακάτω οδηγίες ώστε να μειώνουμε την απώλεια θρεπτικών συστατικών και να ετοιμάζουμε πιο υγιεινά φαγητά:

Βράσιμο: Χρησιμοποιήστε όσο το δυνατό λιγότερο νερό, διότι τα θρεπτικά συστατικά μεταφέρονται από τα τρόφιμα στα υγρά που χρησιμοποιούνται κατά το μαγείρεμα. Κόβουμε τα λαχανικά σε μεγάλα κομμάτια και τα μαγειρεύουμε σύντομα μετά τον τεμαχισμό. Μπορείτε να χρησιμοποιήσετε το υγρό μέσα στο οποίο βράσατε τα λαχανικά, σε άλλες συνταγές (π.χ. ρύζι). Μία καλή πρόταση για την καλύτερη διατήρηση των θρεπτικών συστατικών των λαχανικών είναι οι σούπες (π.χ. χορτόσουπα, ψαρόσουπα, κοτόσουπα).

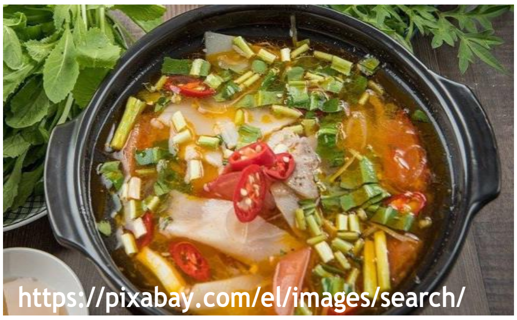

Στον ατμό: Το μαγείρεμα στον ατμό διατηρεί καλύτερα τη γεύση και τα θρεπτικά συστατικά των τροφίμων. Για μαγείρεμα στον ατμό, αρκεί ένα τρυπητό μεταλλικό σκεύος (π.χ. σουρωτήρι) πάνω από μία κατσαρόλα που βράζει!

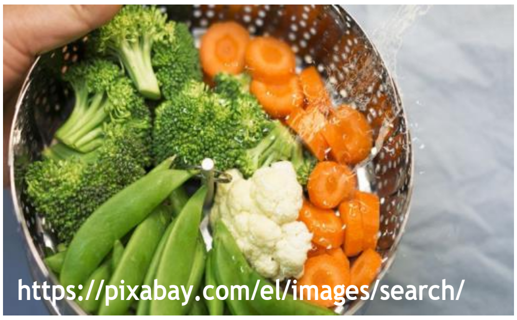

Τηγάνισμα: Καλό είναι να αποφεύγετε το συχνό τηγάνισμα των τροφίμων καθώς συμβάλλει στην πρόσληψη μεγάλης ποσότητας λιπών και κατά συνέπεια θερμίδων, ενώ η πολύ υψηλή θερμοκρασία υποβαθμίζει την ποιότητα των τροφών. Ωστόσο, εάν

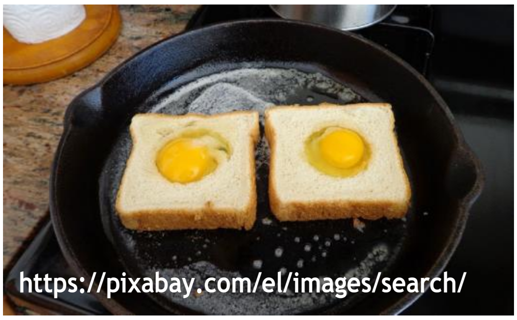

επιθυμείτε να τηγανίσετε π.χ. ψάρι, το ελαιόλαδο είναι η καλύτερη επιλογή. Επιπλέον, το τηγάνισμα τύπου «γουόκ» (τηγάνισμα χωρίς βύθιση στο έλαιο σε χαμηλότερη θερμοκρασία, για λιγότερη ώρα και με λιγότερο ελαιόλαδο), είναι ένας αποδεκτός εναλλακτικός τρόπος μαγειρέματος λαχανικών και προετοιμασίας φαγητού. Μην ξεχνάτε τη χρήση της λαδόκολλας: με λίγο λάδι στον αέρα, φτιάχνετε υπέροχες πατάτες σαν τηγανιτές!

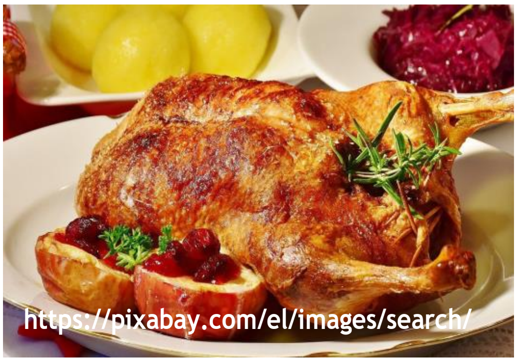

Ψήσιμο στον φούρνο: Όταν ψήνετε κρέας, κοτόπουλο ή ψάρι σε ταψί, αποφύγετε την προσθήκη λαδιού καθώς βγάζουν τα δικά τους υγρά και ψήνονται μέσα σε αυτά. Επιπλέον η χρήση γάστρας στο φούρνο αποτελεί μέρος της παραδοσιακής ελληνικής μαγειρικής και είναι μια υγιεινή επιλογή μαγειρέματος με ελάχιστα υγρά, σε χαμηλή θερμοκρασία.

Ψήσιμο στα κάρβουνα: Το μαρινάρισμα του κρέατος πριν το ψήσιμο, η χαμηλότερη θερμοκρασία, ο έλεγχος των ξεσπασμάτων της φλόγας, και το να μην «παραψήνετε» το κρέας, κάνουν το ψήσιμο στα κάρβουνα πιο ασφαλές καθώς ελαχιστοποιείται η παραγωγή δυνητικά καρκινογόνων ουσιών λόγω των υψηλών θερμοκρασιών ή του καπνού που παράγεται όταν το λίπος στάζει στα κάρβουνα.

Για την **ενίσχυση της γεύσης** των φαγητών, μπορείτε να χρησιμοποιείτε μυρωδικά, όπως άνηθο, ρίγανη, δεντρολίβανο, βασιλικό, δυόσμο, δάφνη, θυμάρι, καθώς και ξίδι, λεμόνι, σκόρ- δο, κρεμμύδι κ.ά. ώστε να μειώσετε την ποσότητα αλατιού που βάζετε στο φαγητό. **Λειτουργή- στε ως πρότυπο** για τα παιδιά σας και μην αλατίζετε το φαγητό σας στο τραπέζι.

Διατροφική Συμπεριφορά Του Παιδιού

Όλα τα παιδιά δεν έχουν την ίδια διατροφική συμπεριφορά. Η διατροφική μας συμπεριφορά έχει γενετική βάση, αλλά διαμορφώνεται σε μεγάλο βαθμό από το περιβάλλον. Σύμφωνα με τις μελέτες των Wardle και συνεργατών, καθώς και άλλων ερευνητών, οι παρακάτω διατροφικές συμπεριφορές έχουν συσχετιστεί στατιστικά με δυσκολίες στη ρύθμιση του σωματικού βάρους σε παιδιά και εφήβους.

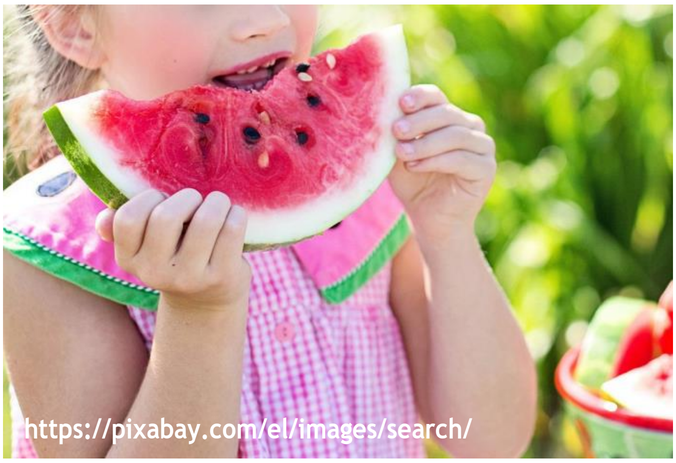
https://pixabay.com/el/images/search/

Χαμηλός βαθμός ανταπόκρισης στο αίσθημα κορεσμού (low satiety responsiveness)

Κάποια παιδιά κληρονομούν μια τάση να μη χορταίνουν εύκολα (π.χ. τρώνε ποσότητες φαγητού μεγαλύτερες από παιδιά ανάλογης ηλικίας και σωματότυπου, ζητάνε συχνά δεύτερο πιά- το φαγητού ή τρώνε γρήγορα το φαγητό που τους έχουμε σερβίρει, χωρίς όμως να αισθάνονται ότι έχουν χορτάσει).

Ως γονέας μπορώ να βοηθήσω με τα παρακάτω:

1. Στα κύρια γεύματα (πρωινό, μεσημεριανό, βραδινό) σερβίρω την κατάλληλη μερίδα για την ηλικία και το βάρος του παιδιού, αλλά την τελική ποσότητα κατανάλωσης την καθορίζει το παιδί (αυτορρύθμιση ποσότητας φαγητού).
2. Ισορροπημένο πιάτο στα κύρια γεύματα, δηλαδή τρόφιμα από όλες τις βασικές κατηγορίες τροφίμων σε κάθε κύριο γεύμα, έτσι ώστε να κινητοποιηθούν στο μέγιστο δυνατό βαθμό οι ορμόνες του κορεσμού, οι οποίες εκκρίνονται από το πεπτικό μας σύστημα κατά τη διάρκεια ενός γεύματος.

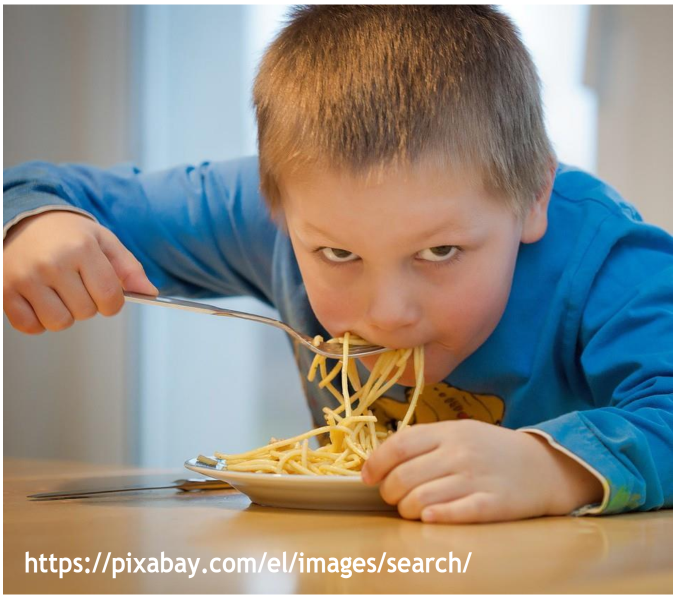
https://pixabay.com/el/images/search/

3. Αργός ρυθμός σίτισης (κατά τη διάρκεια ενός κύριου γεύματος ο ρυθμός σίτισης κανονικά επιβραδύνεται, αν αυτό δε συμβαίνει στο παιδί μου, προσπαθώ να το ενθαρρύνω με ήπιο τρόπο να φάει πιο αργά).
4. Για ορισμένα παιδιά που δε χορταίνουν εύκολα στα κύρια γεύματα, μπορεί να βοηθήσει αν τα ενδιάμεσα μικρογεύματα (σνακ) είναι υψηλής θρεπτικής και θερμιδικής αξίας (π.χ. σπιτικό κέικ με ελαιόλαδο, σπιτική τυρόπιτα).

Υψηλός βαθμός ανταπόκρισης στην έκθεση ορισμένων τροφίμων (food responsiveness)

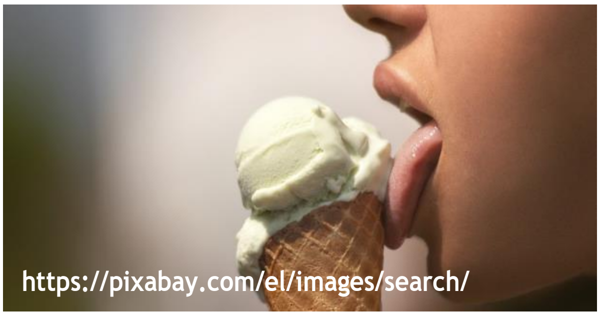
https://pixabay.com/el/images/search/

Αρκετά παιδιά αλλά και ενήλικες αισθάνονται έντονη επιθυμία για κατανάλωση συγκεκριμένων εύγευστων τροφίμων (π.χ. σοκολάτα, πατατάκια) όταν εκτεθούν στη θέα, οσμή ή / και γεύση αυτών των τροφίμων, ανεξάρτητα από την αίσθηση της πείνας. Επειδή τα περισσότερα από αυτά τα τρόφιμα έχουν πολλές θερμίδες, αλλά είναι χαμηλής διατροφικής αξίας μπορώ ως γονέας να βοηθήσω με τα παρακάτω :

1. Στο σπίτι περιορίζω τη διαθεσιμότητα των συγκεκριμένων τροφίμων χωρίς όμως να απαγορεύω την κατανάλωσή τους (προμήθεια σε ειδικές περιστάσεις π.χ. γιορτές, γενέθλια) και αυξάνω τη διαθεσιμότητα των υγιεινών τροφίμων.
2. Προσπαθώ να λειτουργώ ως πρότυπο για το παιδί μου κάνοντας υγιεινές επιλογές σνακ και για τον εαυτό μου.
3. Με τα μεγαλύτερα παιδιά διαπραγματεύομαι τους κανόνες που υπάρχουν σχετικά με την κατανάλωση των εύγευστων, υπερθερμιδικών σνακ λαμβάνοντας υπόψιν τις επιθυμίες όλων των μελών της οικογένειας.
4. Ενθαρρύνω το παιδί μου να συμμετέχει ενεργά στην προετοιμασία όχι μόνο των γευμάτων αλλά και των σνακ όπως σπιτικά πιο υγιεινά γλυκά π.χ. κέικ με ελαιόλαδο, αλεύρι ολικής άλεσης και τη μισή από την προτεινόμενη ζάχαρη.

Μεγάλη επιλεκτικότητα στο φαγητό (food fussiness)

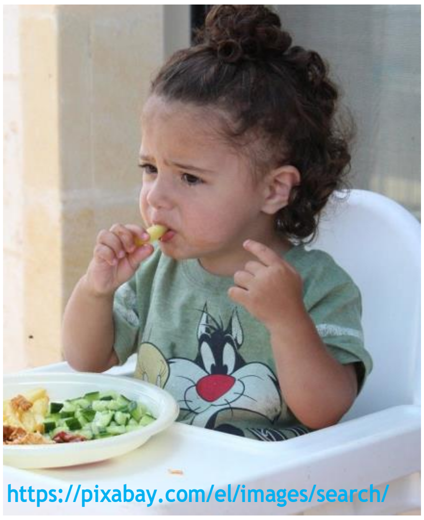
https://pixabay.com/el/images/search/

Ενός βαθμού επιλεκτικότητα στο φαγητό είναι αναμενόμενη στα παιδιά και στους εφήβους. Η προτίμηση των παιδιών για γλυκιές και αλμυρές τροφές σε σύγκριση με τις ξινές και κυρίως τις πικρές τροφές όπως κάποια λαχανικά, οφείλεται εν μέρει σε γενετικούς παράγοντες π.χ. οι γενετικοί πολυμορφισμοί (παραλλαγές) του γονιδίου PROP για την πικρή γεύση. Η ευαισθησία όσον αφορά στο πικρό μειώνεται με την πρόοδο της ηλικίας και την επαναλαμβανόμενη έκθεση των παιδιών σε ποικιλία τροφίμων. Όμως ορισμένα παιδιά έχουν πολύ περιορισμένες διατροφικές επιλογές, οι οποίες ενδέχεται να δημιουργούν πρόβλημα στην υιοθέτηση υγιεινών διατροφικών συνηθειών. Ως γονέας μπορώ να βοηθήσω με τα παρακάτω:

1. Επαναλαμβανόμενες εκθέσεις του παιδιού στα «δύσκολα» τρόφιμα χωρίς πίεση για κατανάλωση, αλλά με ήπια ενθάρρυνση για δοκιμή.
2. Επίσης βοηθά ο συνδυασμός γεύσεων (π.χ. «δύσκολο» φαγητό μαζί με τρόφιμα που συνήθως τρώνε τα παιδιά, π.χ. ψάρι με πατάτες στο φούρνο).
3. Η επιβράβευση βοηθά εφόσον είναι μικρή, λεκτική (όχι υλική) και αφορά περισσότερο την ποιότητα (π.χ. να δοκιμάσει το τρόφιμο) και όχι την ποσότητα (π.χ. να το καταναλώσει όλο).
4. Ευκαιρίες για ενεργή συμμετοχή των παιδιών στην προετοιμασία των γευμάτων (π.χ. μαγείρεμα, οικογενειακή λίστα για ψώνια).

Γονεϊκές Πρακτικές Για Ρύθμιση Βάρους Των Παιδιών

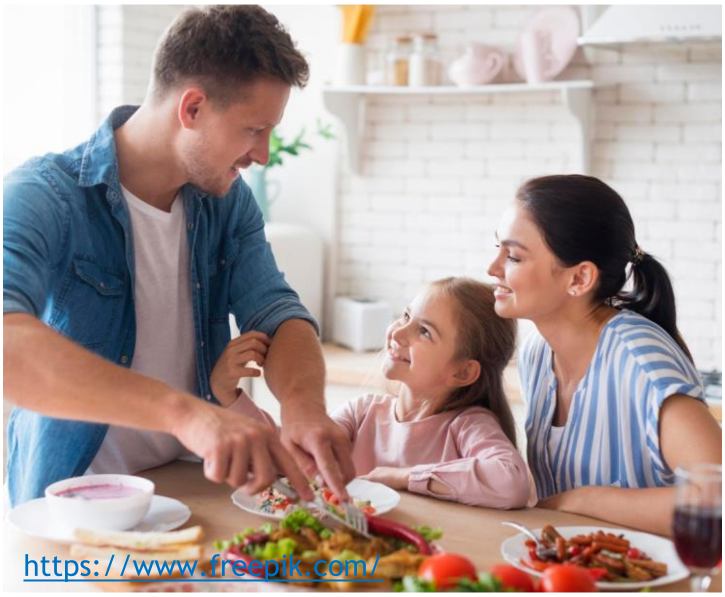
https://www.freepik.com/

Οι γονεϊκές πρακτικές σίτισης αποτελούν ειδικές συμπεριφορές που χρησιμοποιούν οι γονείς για να επηρεάσουν την ποσότητα ή / και την ποιότητα της τροφής που τρώει το παιδί. Πολλές μελέτες καταλήγουν στο συμπέρασμα ότι υπάρχει συσχέτιση ανάμεσα σε γονεϊκά χαρακτηριστικά και γονεϊκές πρακτικές με τις συνήθειες των παιδιών που επηρεάζουν το βάρος τους. Έμφαση δίνεται στη θετική γονεϊκότητα και στη χρήση υποστηρικτικών πρακτικών σίτισης με λιγότερο αυστηρό έλεγχο, ώστε τα παιδιά να αποκτήσουν υγιεινές συνήθειες και την ικανότητα για αυτορρύθμιση του βάρους τους.

Στο σημερινό παχυσαρκιογενές περιβάλλον με τις πολλές φθηνές, νόστιμες και υπερθερμιδικές τροφικές επιλογές, ο ρόλος των γονέων στην υιοθέτηση υγιεινών διατροφικών συνηθειών των παιδιών τους δυσχεραίνεται αρκετά. Οι τρεις διαστάσεις της γονεϊκότητας, δηλαδή η θετική γονεϊκή συμμετοχή, η προαγωγή της αυτονομίας και η παροχή δομής, όπως τονίζεται από τη θεωρία του Αυτοπροσδιορισμού (Self Determination Theory) των Ryan και Deci, διευκολύνουν τη διαδικασία της υιοθέτησης των γονεϊκών αξιών όσον αφορά στις υγιεινές διατροφικές συνήθειες. Παρακάτω περιγράφονται οι κύριες πρακτικές σίτισης που συμβάλλουν στην ικανότητα των παιδιών να κάνουν υγιεινές διατροφικές επιλογές με βάση πρόσφατη κατηγοριοποίηση από τους ερευνητές Di Pascuale και Rivolta με βάση τη θεωρία του Αυτοπροσδιορισμού:

Γονεϊκή παροχή δομής και πρακτικές σίτισης που προάγουν την ικανότητα

Η παροχή δομής στη σίτιση από την πλευρά των γονέων είναι πολύ σημαντική για την ικανό- τητα του παιδιού να υιοθετήσει τις γονεϊκές αξίες όσον αφορά στη διατροφή, αλλά και την ικανό- τητά του για αυτορρύθμιση της διατροφικής του συμπεριφοράς. Στην κατηγορία αυτή ανήκουν οι παρακάτω πρακτικές σίτισης:

Κανόνες και όρια

Οι κανόνες και τα όρια αναφέρονται στις προσδοκίες των γονέων σε σχέση με τι, πότε και πόσο φαγητό θα έπρεπε να καταναλώνουν τα παιδιά τους. Περιλαμβάνουν ποικιλία κανόνων σχετικούς με την ποιότητα των φαγητών που επιτρέπεται να καταναλώνουν τα παιδιά, το πότε επιτρέπεται η κατανάλωση των λιγότερο υγιεινών τροφίμων (π.χ. διαφορά ανάμεσα σε γεύματα ή σνακ τις καθημερινές ή το Σαββατοκύριακο ή σε ειδικές περιπτώσεις), αλλά και με την ποσότητα των λιγότερο υγιεινών, υπερθερμιδικών τροφίμων που επιτρέπεται να καταναλώνουν τα

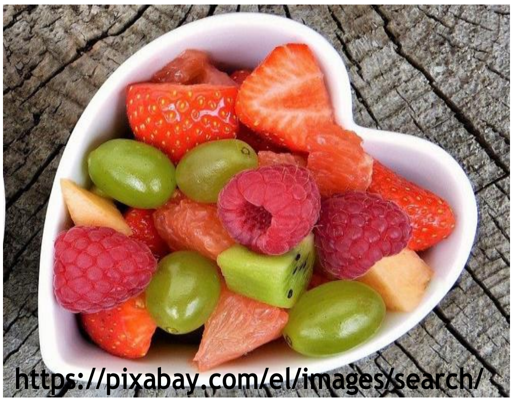

παιδιά. Η παρουσία ξεκάθαρων κανόνων και ορίων, οι οποίοι είναι αποδεκτοί από όλη την οικογένεια βοηθούν στη μείωση της χρήσης περιοριστικών πρακτικών σίτισης από τους γονείς.

Διαθεσιμότητα και προσβασιμότητα τροφίμων

Η διαθεσιμότητα τροφίμων ορίζεται ως η παρουσία ή απουσία συγκεκριμένων τροφίμων στο σπίτι, ενώ η προσβασιμότητα αναφέρεται στις πρακτικές που χρησιμοποιούν οι γονείς για να ελέγξουν πόσο εύκολο ή δύσκολο είναι για το παιδί να έχει πρόσβαση σε συγκεκριμένα τρόφιμα μόνο του ή με μικρή βοήθεια. Τέτοιες πρακτικές περιλαμβάνουν τη φύλαξη των λιγότερο υγιεινών τροφίμων σε σημείο που δεν μπορούν να τα φτάσουν τα παιδιά ή το να έχουν υγιεινά τρόφιμα σε εμφανές μέρος π.χ. φρούτα σε μπωλ στο τραπέζι. Η διαθεσιμότητα και η προσβασιμότητα τροφίμων καθορίζουν σε μεγάλο βαθμό το οικογενειακό περιβάλλον σε σχέση με τη σίτιση του παιδιού.

Γονείς ως πρότυπα υγιεινής διατροφής

Οι γονείς αποτελούν πολύ σημαντικά πρότυπα συμπεριφοράς για τα παιδιά τους. Πολλές έρευνες καταλήγουν στο συμπέρασμα ότι τόσο οι υγιεινές όσο και οι ανθυγιεινές συνήθειες των παιδιών εξαρ- τώνται σημαντικά από τις αντίστοιχες συνήθειες των γονέων τους.

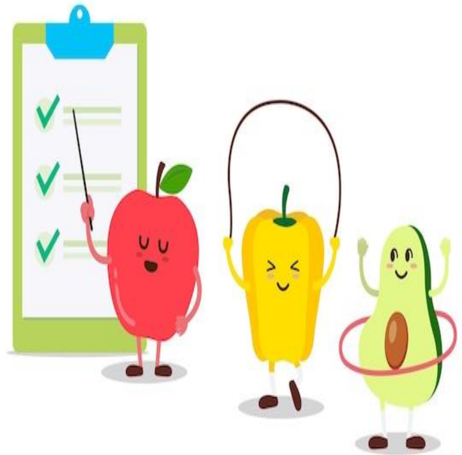

https://www.freepik.com/

Διατροφική εκπαίδευση

Αναφέρεται στην προσπάθεια των γονέων να μεταφέρουν πληροφορίες στα παιδιά με κατάλληλο τρόπο για την ηλικία και το αναπτυξιακό τους στάδιο, με σκοπό τα ίδια τα παιδιά σταδιακά να αισθανθούν ικανά να κάνουν τις σωστές επιλογές όσον αφορά στα τρόφιμα που τρώνε. Το παρόν εκπαιδευτικό υλικό έχει το σκοπό να διευκολύνει αυτή τη διαδικασία της μετάδοσης γνώσεων αναφορικά με τη μεσογειακή διατροφή και τα πολλαπλά οφέλη που έχει για τα παιδιά αλλά και τους γονείς τους.

Ενθάρρυνση

Αναφέρεται στους τρόπους με τους οποίους οι γονείς προσπαθούν με υποστηρικτικό, θετικό και ήπιο τρόπο να κάνουν τα παιδιά τους να υιοθετήσουν υγιεινές διατροφικές συνήθειες και να αποφύγουν λιγότερο υγιεινές επιλογές όπως π.χ. όταν ζητάνε με ευγενικό και ήπιο τρόπο από

το παιδί να δοκιμάσει ένα καινούργιο φαγητό σε αντίθεση με την πίεση για φαγητό, όπου ο γονέας απαιτεί από το παιδί να δοκιμάσει το φαγητό.

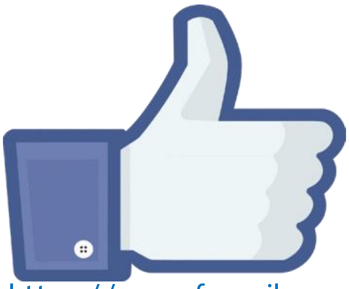

https://www.freepik.com/

Επιβράβευση

Η επιβράβευση αποτελεί είδος θετικής ενίσχυσης, κατά την οποία ο γονέας δίνει ανατροφοδότηση στο παιδί όσον αφορά στη συμπεριφορά του. Ιδίως για τα μικρότερα παιδιά είναι αποτελεσματική η χρήση του επαίνου (κυρίως του λεκτικού) όταν προσπαθούν να βελτιώσουν τις διατροφικές τους συνήθειες (π.χ. όταν δοκιμάζουν ένα καινούργιο φαγητό).

Θετική γονεϊκή συμμετοχή και πρακτικές σίτισης που προάγουν την συντροφικότητα / σχετικότητα

Εκτός από την παροχή δομής στη σίτιση των παιδιών, η θετική γονεϊκή συμμετοχή θεωρείται πολύ σημαντική για την αποτελεσματική εσωτερίκευση των υγιεινών διατροφικών συνηθειών εκ μέρους των παιδιών και περιλαμβάνει πρακτικές όπως τα οικογενειακά γεύματα και η συμμετοχή του παιδιού στην προετοιμασία των γευμάτων.

Τα **οικογενειακά γεύματα** και η **συμμετοχή του παιδιού στην προετοιμασία των γευμάτων** αποτελούν πρακτικές μέσω των οποίων οι γονείς μπορούν να διευκολύνουν την αυτορρύθμιση του παιδιού και την εσωτερίκευση των υγιεινών διατροφικών συνηθειών που επιθυμούν οι γονείς να υιοθετήσουν τα παιδιά τους, μέσω του ενδιαφέροντος και της φροντίδας του παιδιού στον τομέα της διατροφής. Όταν τα παιδιά έχουν τη αίσθηση της θετικής συσχέτισης με τους γονείς τους, η οποία χτίζεται πάνω στις κοινές θετι- κές εμπειρίες στον τομέα της διατροφής (π.χ. συχνά οικο- γενειακά γεύματα με ευχάρι- στη ατμόσφαιρα, από κοινού σχεδιασμός και προετοιμασία γευμάτων), αυξάνονται οι πι- θανότητες να αποδεχτούν τις προσπάθειες των γονέων για υιοθέτηση των επιθυμητών διατροφικών συμπεριφορών.

Γονεϊκή υποστήριξη της αυτονομίας και πρακτικές σίτισης που προάγουν την αυτονομία

Εκτός από την ανάγκη των παιδιών για ικανότητα και συντροφικότητα, σημαντική για την επίτευξη αποτελεσματικής αυτορρύθμισης είναι και η υποστήριξη της αυτονομίας του παιδιού. Δηλαδή να αισθάνονται τα παιδιά ότι η συμπεριφορά τους πηγάζει εκ των έσω και δεν συμμορφώνονται απλά με τις υποδείξεις των γονέων τους.

Οι παρακάτω πρακτικές θεωρούνται ότι βοηθούν στην αυτονόμηση των παιδιών σε θέματα διατροφής:

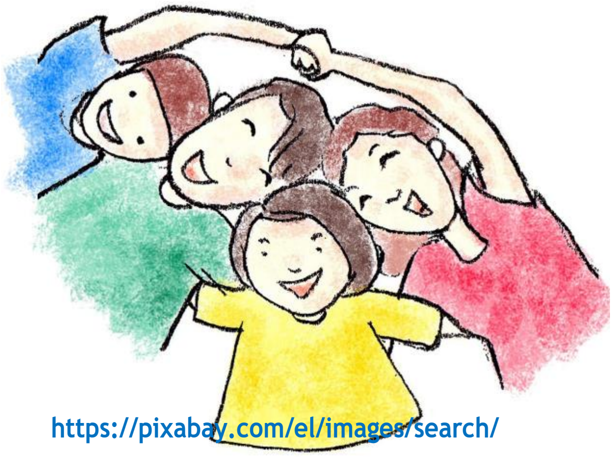

https://pixabay.com/el/images/search/

Συζήτηση και διαπραγμάτευση των κανόνων που σχετίζονται με τη διατροφή

Για τα μεγαλύτερα παιδιά είναι σημαντικό να γίνεται επεξήγηση των κανόνων που σχετίζονται με τη διατροφή και να συμμετέχουν και τα ίδια στην δημιουργία των κανόνων που αφορούν όλη την οικογένεια. Οι κανόνες δεν πρέπει να είναι άκαμπτοι, αλλά ευέλικτοι και οι γονείς διαθέσιμοι να τους διαπραγματευτούν λαμβάνοντας υπόψιν τις επιθυμίες των παιδιών, αλλά και τις συνήθειες όλων των μελών της οικογένειας.

Περιορισμένες επιλογές

Τα παιδιά έχουν την ανάγκη να επιλέγουν ελεύθερα τι θα φάνε και αν θα φάνε τα τρόφιμα που τους προσφέρουν οι γονείς τους. Οι γονείς μέσω των περιορισμένων ή κατευθυνόμενων επιλογών, οι οποίες λαμβάνουν υπόψιν τις γευστικές προτιμήσεις του παιδιού, βοηθούν στην αυτονόμηση του παιδιού σε θέματα διατροφής. Για παράδειγμα το παιδί επιλέγει τι και πόσο θα φάει στο γεύμα από τα υγιεινά τρόφιμα που έχει επιλέξει ο γονιός ως κατάλληλες επιλογές για την ηλικία του.

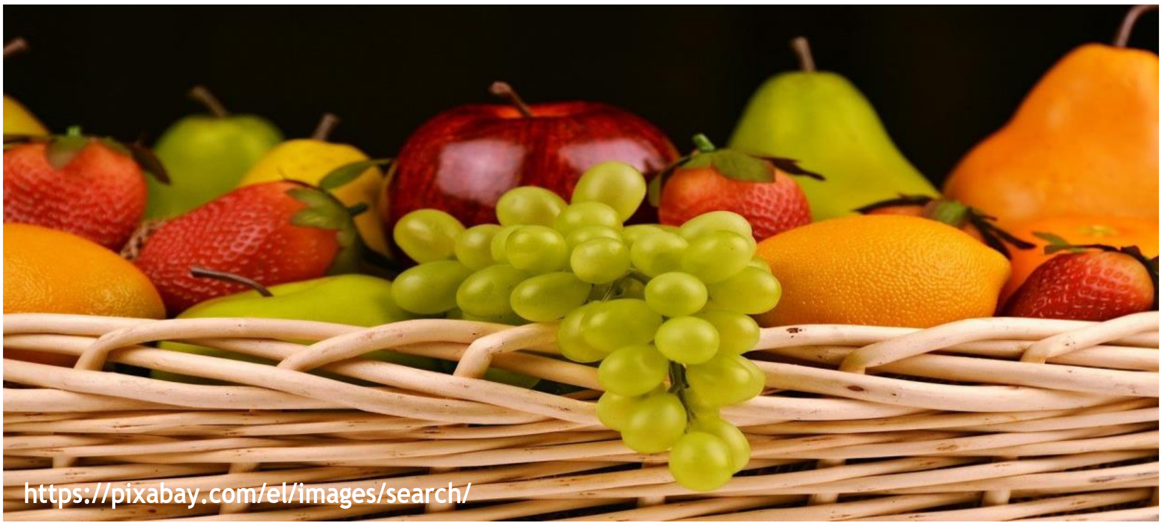

https://pixabay.com/el/images/search/

Λίγα λόγια για τη θεωρία του Αυτοπροσδιορισμού των Ryan και Deci (Self Determination Theory)

Η θεωρία του Αυτοπροσδιορισμού ανήκει στις θεωρίες των κινήτρων με εφαρμογές στον τομέα της εκπαίδευσης, της γονεϊκότητας, αλλά και της υγείας. Σύμφωνα με τη θεωρία αυτή όλοι οι άνθρωποι έχουν τρεις έμφυτες βασικές ανάγκες που πρέπει να πληρούνται για να επιτευχθεί η αυτορρύθμιση και η προσωπική ευημερία. Η πρώτη αφορά την ανάγκη για ανάπτυξη δεξιοτήτων / ικανοτήτων (competence), η οποία ικανοποιείται στα παιδιά μέσω της καθοδήγησης από σημαντικούς άλλους ανθρώπους-πρότυπα (π.χ. γονείς, δασκάλους) και αφορά στο αίσθημα αποτελεσματικότητας και επιτυχίας ενώ το άτομο αλληλεπιδρά με το περιβάλλον. Η δεύτερη αφορά την ανάγκη για συντροφικότητα / σχετικότητα (relatedness), η οποία ικανοποιείται από τη δημιουργία κοινωνικών σχέσεων και την άντληση αισθημάτων ασφάλειας από κατάλλη- λες διαπροσωπικές σχέσεις (π.χ. οικογένεια, σχολείο, φίλοι). Η τρίτη αφορά την ανάγκη για αυτονομία (autonomy), η οποία ικανοποιείται από τη δυνατότητα των παιδιών να λαμβάνουν ελεύθερα αποφάσεις, να κάνουν επιλογές και να αισθάνονται ότι η συμπεριφορά τους είναι αυτοκαθοριζόμενη. Στον τομέα της διατροφής, η εσωτερίκευση των γονεϊκών αξιών σε σχέση με τις διατροφικές συνήθειες των παιδιών τους διευκολύνεται εφόσον οι γονείς χρησιμοποιούν όσο πιο συχνά γίνεται πρακτικές σίτισης που ικανοποιούν και τις τρεις αυτές ανάγκες, όπως φαίνεται παραστατικά στο παρακάτω σχήμα:

Σχηματική αναπαράσταση της σχέσης της θεωρίας του Αυτοπροσδιορισμού των Ryan & Deci με τις γονεϊκές πρακτικές σίτισης με βάση την κατάταξη των Di Pascuale & Rivolta:

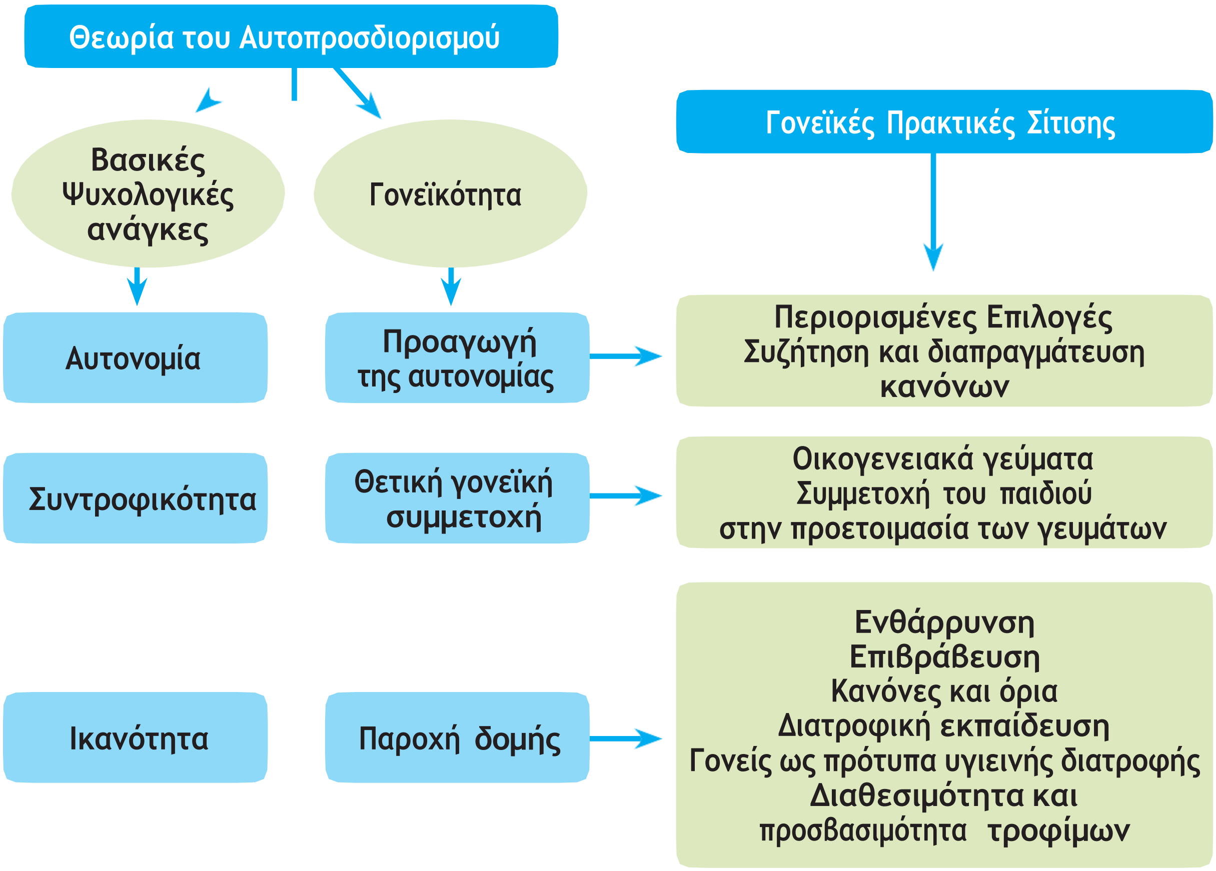

Συνοψίζοντας, θέλουμε να τονίσουμε το γεγονός ότι στο ιατρείο μας σύμβουλοι, παιδιά και γονείς γινόμαστε μια ομάδα, η οποία δεν έχει ως στόχο την απώλεια βάρους μέσω τήρησης ενός αυστηρού διαιτολογίου, αλλά θέτει ως κύριο στόχο την αυτορρύθμιση των παιδιών σε θέματα βάρους και συμπεριφορών υγείας, έτσι ώστε να έχουμε τα καλύτερα δυνατά μακροχρόνια αποτελέσματα. Για να το πετύχουμε αυτό χρειάζεται ομαδική δουλειά, εκπαίδευση με κατάλληλο τρόπο για την ηλικία και το αναπτυξιακό στάδιο των παιδιών, σεβασμό στις αξίες και στην κουλτούρα της κάθε οικογένειας, εξατομίκευση των στόχων και συχνές συνεδρίες, έτσι ώστε να μπορούμε να θέτουμε μικρούς διαδοχικούς στόχους, ξεκινώντας από τους πιο εύκολους και σταδιακά αυξάνοντας τη δυσκολία, με απώτερο στόχο την μακροχρόνια ρύθμιση του βάρους των παιδιών εντός φυσιολογικών ορίων.
