# Supplementary material for: The ENDORSE Feasibility Study: Exploring the Use of M-Health, Artificial Intelligence and Serious Games for the Management of Childhood Obesity
Source: Nutrients. 2023 Mar 17;15(6):1451. doi: 10.3390/nu15061451 (PMC10057317; doi:10.3390/nu15061451)
Supplement: Supplementary file 1 [file nutrients-15-01451-s001.zip › Health behaviors questionnaire.pdf]

HEALTH BEHAVIORS QUESTIONNAIRE

Food habits

1. How many servings of fruit does your child consume (average)?

   (1 serving of fruit = 120-200g. of fruit, e.g. 1 medium-sized fruit e.g. apple, pear, orange, peach, banana, 2 small fruits (e.g. tangerines, plums, kiwis, about 8 strawberries , about 15 cherries, about 30 grapes, 1 slice of watermelon, 4 dried fruits, 1 ½ tablespoon raisins)

   Never /rarely ☐ 1-3 / month ☐ 1-2 / week ☐ 3-4 / week ☐

   5-7 / week ☐ 1-2 / day ☐ 3-4 / day ☐ ≥ 5 / day ☐

2. How many servings of vegetables (raw or boiled or steamed or grilled) does your child consume (average)?

   (1 serving = 150-200g raw or cooked vegetables, e.g. 1 medium cucumber, 2 medium carrots, 1 large tomato or 1 cup of 240 ml grated tomato, 1 cup of 240 ml cooked or chopped raw vegetables, 2 cups of 240 ml raw green leafy vegetables, e.g. lettuce, arugula, etc.)

   Never /rarely ☐ 1-3 / month ☐ 1-2 / week ☐ 3-4 / week ☐

   5-7 / week ☐ 1-2 / day ☐ 3-4 / day ☐ ≥ 5 / day ☐

3. How many servings of dairy products (a 250 ml glass of milk , a 200g cup of yogurt, a slice of cheese) does your child consume (average)?

   Never /rarely ☐ 1-3 / month ☐ 1-2 / week ☐ 3-4 / week ☐

   5-7 / week ☐ 1-2 / day ☐ 3-4 / day ☐ ≥ 5 / day ☐

4. How many servings of cereal does your child consume (average)?

   (1 serving = 1 slice of bread -30gr, 2 rusks, 1 medium-sized round rusk, ½ 240ml cup of cooked pasta or rice or groats - 70-90gr, 240 240ml cup of breakfast cereal - 30gr, koulouri, 1 medium-sized potato - 120-150g cooked)

   1-2 servings / day ☐ 3-4 servings / day ☐ 5-6 servings / day ☐

   7 - 8 servings / day ☐ ≥ 9 servings / day ☐

5. From the above portions of cereals that your child usually consumes, how many contain whole grains such as whole grain toast, whole grain bread or multigrain, whole grain rusks, whole grain pasta, pop corn , breakfast cereals with fiber content> 3g / 100g product:

   None ☐ 1-2 servings / day ☐ 3-4 servings / day ☐

   5-6 servings / day ☐ 7 - 8 servings / day ☐ ≥ 9 servings / day ☐

6. How often does your child eat white meat (chicken, turkey, duck, rabbit)?

Never /rarely ☐ 1-3 / month ☐ 1-2 / week ☐ 3-4 / week ☐
5-7 / week ☐ 1-2 / day ☐ 3-4 / day ☐ ≥ 5 / day ☐

7. How often does your child eat red meat (beef, pork, goat, lamb)?

Never /rarely ☐ 1-3 / month ☐ 1-2 / week ☐ 3-4 / week ☐
5-7 / week ☐ 1-2 / day ☐ 3-4 / day ☐ ≥ 5 / day ☐

8. How often does your child eat processed meat (e.g. a slice of ham, a slice of turkey, a slice of salami, a medium-sized sausage)

Never /rarely ☐ 1-3 / month ☐ 1-2 / week ☐ 3-4 / week ☐
5-7 / week ☐ 1-2 / day ☐ 3-4 / day ☐ ≥ 5 / day ☐

9. If your child eats processed meat, specify the type:

Slice of ham ☐ Slice of turkey ☐
Slice of salami ☐ Sausage ☐ Other: ……………………………… ..

10. How often does your child eat fish or seafood?

Never /rarely ☐ 1-3 / month ☐ 1-2 / week ☐ 3-4 / week ☐
5-7 / week ☐ 1-2 / day ☐ 3-4 / day ☐ ≥ 5 / day ☐

11. If your child ate fish or seafood in the previous month, specify the kind:

Sardines ☐ Anchovy ☐ Picarel ☐ Sea bream ☐ Cod ☐
Salmon ☐ Octopus ☐ Squid ☐ Shrimp ☐
Frozen fishsticks ☐ Other: ………………………….

12. How often does your child eat legumes?

Never /rarely ☐ 1-3 / month ☐ 1-2 / week ☐ 3-4 / week ☐
5-7 / week ☐ 1-2 / day ☐ 3-4 / day ☐ ≥ 5 / day ☐

13. How often do you use olive oil when cooking?

Never /rarely ☐ 1-3 / month ☐ 1-2 / week ☐ 3-4 / week ☐
5-7 / week ☐ 1-2 / day ☐ 3-4 / day ☐ ≥ 5 / day ☐

14. How often do you use other vegetable oils (e.g. sunflower oil, corn oil, soybean oil) when cooking?

Never /rarely ☐	1-3 / month ☐	1-2 / week ☐	3-4 / week ☐
5-7 / week ☐	1-2 / day ☐	3-4 / day ☐	≥ 5 / day ☐

15. How often do you use butter?

Never /rarely ☐	1-3 / month ☐	1-2 / week ☐	3-4 / week ☐
5-7 / week ☐	1-2 / day ☐	3-4 / day ☐	≥ 5 / day ☐

16. How often do you use margarine?

Never /rarely ☐	1-3 / month ☐	1-2 / week ☐	3-4 / week ☐
5-7 / week ☐	1-2 / day ☐	3-4 / day ☐	≥ 5 / day ☐

17. How often does your child eat nuts (1 serving = a handful of nuts (e.g. 18 almonds, 6 whole walnuts, 3 tablespoons of sunflower seeds) or their products such as tahini, peanut butter etc. (one serving = 1½ tablespoon of 15ml or 25g, e.g. tahini)

Never /rarely ☐	1-3 / month ☐	1-2 / week ☐	3-4 / week ☐
5-7 / week ☐	1-2 / day ☐	3-4 / day ☐	≥ 5 / day ☐

18. How often does your child eat “ladero” (mainly vegetarian dish cooked in olive oil e.g. green beans, peas)?

Never /rarely ☐	1-3 / month ☐	1-2 / week ☐	3-4 / week ☐
5-7 / week ☐	1-2 / day ☐	3-4 / day ☐	≥ 5 / day ☐

19. How often does your child eat eggs (in any form, boiled, fried, scrambled eggs, in recipes)?

Never /rarely ☐	1-3 / month ☐	1-2 / week ☐	3-4 / week ☐
5-7 / week ☐	1-2 / day ☐	3-4 / day ☐	≥ 5 / day ☐

20. How often does your child eat fast food (eg gyros, pizza, pre-cooked meals)?

Never /rarely ☐	1-3 / month ☐	1-2 / week ☐	3-4 / week ☐
5-7 / week ☐	1-2 / day ☐	3-4 / day ☐	≥ 5 / day ☐

21. How often does your child eat high-calorie packaged products (i.e.> 250 kcal per 100g of the product) and high in simple sugars (i.e. simple sugars> 22.5g per 100g of the product) or high in salt (i.e.> 1.5g. of salt or 0.6g of sodium per 100g of the product) (e.g. 1 bag of chips 70g , 1 croissant 100g, 1 donut 100g, 1 chocolate 100g, ≥ 3 stuffed cookies)

Never /rarely	☐	1-3 / month	☐	1-2 / week	☐	3-4 / week	☐
5-7 / week	☐	1-2 / day	☐	3-4 / day	☐	≥ 5 / day	☐

22. How often does your child drink soft drinks like coca -cola or fanta (330 ml) or packaged juices e.g. amita (1 glass of 250 ml) ;

Never /rarely	☐	1-3 / month	☐	1-2 / week	☐	3-4 / week	☐
5-7 / week	☐	1-2 / day	☐	3-4 / day	☐	≥ 5 / day	☐

CHILD EATING BEHAVIOR

CEBQ by Wardle J et al

(1 = never, 2 = rarely, 3 = sometimes, 4 = often, 5 = always)

FOOD RESPONSIVENESS : My child is always asking for food: Options 1 to 5
Given the choice, my child would eat most of the time: Options 1 to 5
Even if my child is full up she/he finds room to eat her/his favorite food: Options 1 to 5

SATIETY RESPONSIVENESS : My child has a big appetite: Options 1 to 5 (reversed item)
My child leaves food on his/her plate at the end of a meal: Options 1 to 5
My child gets full up easily: Options 1 to 5
My child cannot eat a meal if he/she had a snack just before: Options 1 to 5

FOOD FUSSINESS : My child refuses new food at first: Options 1 to 5
My child is difficult to please with meals: Options 1 to 5
My child enjoys tasting new foods: Options 1 to 5 (reversed item)

Additional nutritional behavior questions:
EATING RATE: SLOW OR FAST (more or less than 15 minutes)

FAMILY MEALS (TIMES / WEEK):

NUMBER OF MAIN MEALS:

NUMBER OF SMALL MEALS / SNACKS:

MEAL FREQUENCY: REGULAR: IRREGULAR:

CHILD FEEDING CAREGIVERS (quality and quantity): MOM, DAD, GRANDMOTHER, GRANDFATHER, OTHER

EXERCISE

What kind of exercise does your child do?

None	
Dance / ballet / gymnastics	
Basketball	
Football	
Martial arts	
Swimming	
Polo	
Other (Record it)	

How many times per week does your child exercise (organized sports)?

Not at all ☐ 1-2 times/week ☐ 3-5 times/week ☐

6-7 times / week ☐

Approximately how many minutes per day is your child physically active (walking, cycling, skating, games with friends that require physical activity, gardening, cooking, housework)

Not at all ☐ <30 minutes ☐ 30 - 60 minutes ☐

60 - 120 minutes ☐ > 120 minutes ☐

SEDENTARY BEHAVIOUR

Approximately how many hours per day (weekdays) does your child spend in front of screens? (e.g. TV, tablet , mobile phone)

<1 hour / day ☐ 1-2 hours / day ☐ 2 - 3 hours / day ☐

3 - 4 hours / day ☐ > 4 hours / day ☐

Approximately how many hours per day (weekends or holidays) does your child spend in front of screens? (e.g. TV, tablet , mobile phone)

<1 hour / day ☐ 1-2 hours / day ☐ 2 - 3 hours / day ☐

3 - 4 hours / day ☐ > 4 hours / day ☐

Approximately how many hours per day does your child play video games on weekdays?

<1 hour / day ☐ 1-2 hours / day ☐ 2 - 3 hours / day ☐

3 - 4 hours / day ☐ > 4 hours / day ☐

Approximately how many hours per day does your child play video games on the weekends?

<1 hour / day ☐ 1-2 hours / day ☐ 2 - 3 hours / day ☐

3 - 4 hours / day ☐ > 4 hours / day ☐

SLEEP

Approximately how many hours per day does your child sleep (average)?

9 - 11 hours ☐ 7 - 8 hours ☐ <7 hours ☐ > 11 hours ☐

How many times a week does your child have difficulty sleeping (e.g. insomnia, difficulty falling asleep, frequent awakenings, teeth grinding)?

Not at all ☐ 1-2 times/week ☐ 3-4 times/week ☐ 5-7 times/week ☐

If there were any difficulties, specify the type:

Insomnia ☐ Difficulty falling asleep ☐ Frequent awakenings☐

Teeth grinding ☐ Waking up very early in the morning ☐

Noisy breathing during sleep ☐ Other: ……………………………… ..
