# Supplementary material for: The ENDORSE Feasibility Study: Exploring the Use of M-Health, Artificial Intelligence and Serious Games for the Management of Childhood Obesity
Source: Nutrients. 2023 Mar 17;15(6):1451. doi: 10.3390/nu15061451 (PMC10057317; doi:10.3390/nu15061451)
Supplement: Supplementary file 1 [file nutrients-15-01451-s001.zip › Post intervention feasibility questionnaire.pdf]

POST INTERVENTION FEASIBILITY QUESTIONNAIRE

GAME

How useful did you find the action missions to increase the child's physical activity?

Not at all ☐ not so ☐ somewhat ☐ very ☐ extremely ☐

How useful did you find the mini game “lunch box "to familiarize the child with the main foods-food groups of the Mediterranean diet?

Not at all ☐ not so ☐ somewhat ☐ very ☐ extremely ☐

How useful did you find the mini game “lunch box ” to familiarize the child with the food proportions of a balanced plate?

Not at all ☐ not so ☐ somewhat ☐ very ☐ extremely ☐

How useful did you find the mini game “balance beam” to familiarize the child with the amounts of food that make up a portion?

Not at all ☐ not so ☐ somewhat ☐ very ☐ extremely ☐

How useful did you find the mini game "fruit ninja" to familiarize the child with categorizing snacks based on their nutritional and caloric value?

Not at all ☐ not so ☐ somewhat ☐ very ☐ extremely ☐

How useful did you find the minigame "traffic light" to familiarize the child with the categorization of fatty acids (fats) according to their quality? *(question for group 3 only)*

Not at all ☐ not so ☐ somewhat ☐ very ☐ extremely ☐

How useful did you find the game’s ability to feature educational messages?

Not at all ☐ not so ☐ somewhat ☐ very ☐ extremely ☐

How relevant to your child’s needs did you find the messages featured through the game?

Not at all ☐ not so ☐ somewhat ☐ very ☐ extremely ☐

How easy was it for your child to use the game in general?

Not at all ☐ not so ☐ somewhat ☐ very ☐ extremely ☐

How easy did you find the action mini games for your child?

Not at all ☐ not so ☐ somewhat ☐ very ☐ extremely ☐

How easy did you find the mini game “lunch box ' for your child?

Not at all ☐ not so ☐ somewhat ☐ very ☐ extremely ☐

How easy did you find the mini game “balance beam” for the child?

Not at all ☐ not so ☐ somewhat ☐ very ☐ extremely ☐

How easy did you find the mini game “fruit ninja” for your child ?

Not at all ☐ not so ☐ somewhat ☐ very ☐ extremely ☐

How easy did you find the mini game “traffic light” for your child ? *(question for group 3 only)*

Not at all ☐ not so ☐ somewhat ☐ very ☐ extremely ☐

How helpful did you find the game in regulating your child's weight?
Not at all ☐ not so ☐ somewhat ☐ very ☐ extremely ☐

How helpful did you find the game in improving the quality of your child’s overall diet?

Not at all ☐ not so ☐ somewhat ☐ very ☐ extremely ☐

How helpful did you find the game in increasing your child's physical activity?

Not at all ☐ not so ☐ somewhat ☐ very ☐ extremely ☐

MOBILE APP

How useful did you find the daily messages the program sent you to achieve your goals?

Not at all ☐ not so ☐ somewhat ☐ very ☐ extremely ☐

How relevant to your child’s needs did you find the messages featured through the mobile app?

Not at all ☐ not so ☐ somewhat ☐ very ☐ extremely ☐

How useful did you find the weekly messages the clinical team sent you through the app to achieve your goals? *(question for group 1 only)*

How useful did you find the function of daily goal recording in the app to achieve your goals?

Not at all ☐ not so ☐ somewhat ☐ very ☐ extremely ☐

How useful did you find the ability to enter the child's weight weekly in the application?

Not at all ☐ not so ☐ somewhat ☐ very ☐ extremely ☐

How useful did you find the presence of your child's nutrition plan in the application?

Not at all ☐ not so ☐ somewhat ☐ very ☐ extremely ☐

How useful did you find the presence of the shopping list in the app?

Not at all ☐ not so ☐ somewhat ☐ very ☐ extremely ☐

How useful did you find the presence of the recipes in the application?

Not at all ☐ not so ☐ somewhat ☐ very ☐ extremely ☐

How useful did you find the presence of the educational material in the application?

Not at all ☐ not so ☐ somewhat ☐ very ☐ extremely ☐

How easy did you find the app to access?

Not at all ☐ not so ☐ somewhat ☐ very ☐ extremely ☐

How easy did you find the app to use in general?

Not at all ☐ not so ☐ somewhat ☐ very ☐ extremely ☐

How easy did you find the function of daily goal recording in the application?

Not at all ☐ not so ☐ somewhat ☐ very ☐ extremely ☐

How easy did you find making entries of your child's weight in the application?

Not at all ☐ not so ☐ somewhat ☐ very ☐ extremely ☐

How helpful did you find the app to regulate your child's weight?
Not at all ☐ a little ☐ moderate ☐ very ☐ very much ☐

How helpful did you find the app in improving the quality of your child's overall diet?

Not at all ☐ not so ☐ somewhat ☐ very ☐ extremely ☐

How helpful did you find the app in increasing your child's physical activity?

Not at all ☐ not so ☐ somewhat ☐ very ☐ extremely ☐

FITBIT ACTIVITY TRACKERS

How easy to use was the activity tracker?

Not at all ☐ not so ☐ somewhat ☐ very ☐ extremely ☐

How useful did you find the activity tracker to monitor the child's physical activity?

Not at all ☐ not so ☐ somewhat ☐ very ☐ extremely ☐

How helpful did you find the activity tracker to increase the child's physical activity?

Not at all ☐ not so ☐ somewhat ☐ very ☐ extremely ☐

GENERAL QUESTIONS

Do you think your child enjoyed the game in general?

Not at all ☐ not so ☐ somewhat ☐ very ☐ extremely ☐

Do you think the game helped your child understand the educational material?

Not at all ☐ not so ☐ somewhat ☐ very ☐ extremely ☐

Do you think the app helped you understand the educational material?

Not at all ☐ not so ☐ somewhat ☐ very ☐ extremely ☐

Would you recommend this game to another parent who wants to help their child manage their weight?
Yes No

Would you recommend the mobile app to another parent who wants to help their child manage their weight?
Yes No

What do you think we could do to improve the app?

………
………

What do you think we could do to improve the game?
………
………
………
